# The *Cardamine hirsuta* genome offers insight into the evolution of morphological diversity

**DOI:** 10.1038/nplants.2016.167

**Published:** 2016-10-31

**Authors:** Xiangchao Gan, Angela Hay, Michiel Kwantes, Georg Haberer, Asis Hallab, Raffaele Dello Ioio, Hugo Hofhuis, Bjorn Pieper, Maria Cartolano, Ulla Neumann, Lachezar A. Nikolov, Baoxing Song, Mohsen Hajheidari, Roman Briskine, Evangelia Kougioumoutzi, Daniela Vlad, Suvi Broholm, Jotun Hein, Khalid Meksem, David Lightfoot, Kentaro K. Shimizu, Rie Shimizu-Inatsugi, Martha Imprialou, David Kudrna, Rod Wing, Shusei Sato, Peter Huijser, Dmitry Filatov, Klaus F. X. Mayer, Richard Mott, Miltos Tsiantis

**Affiliations:** 1 Max Planck Institute for Plant Breeding Research, Carl-von-Linné-Weg 10, 50829 Köln, Germany; 2 Plant Genome and Systems Biology, Helmholtz Zentrum Munich, Ingolstädter Landstrasse 1, 85764 Neuherberg, Germany; 3 Department of Evolutionary Biology and Environmental Studies, University of Zurich, Winterthurerstrasse 190, CH-8057 Zurich, Switzerland; 4 Department of Plant Sciences, University of Oxford, South Parks Road, OX1 3RB Oxford UK; 5 Department of Statistics, University of Oxford, 1 South Parks Road, OX1 3TG Oxford UK; 6 Department of Plant, Soil and Agricultural Systems, Southern Illinois University, Carbondale, 62901 Illinois USA; 7 Arizona Genomics Institute, School of Plant Sciences and BIO5 Institute for Collaborative Research, University of Arizona, 1657 East Helen Street, Tucson, 85721 Arizona USA; 8 UCL Genetics Institute, University College London, Gower Street, WC1E 6BT London UK; 9 Present Address: †Present address: Department of Biology and Biotechnology, Università La Sapienza, P.le Aldo Moro, 5, 00185 Rome, Italy (R.D.I.). The Global Food Security, BBSRC, Polaris House, North Star Avenue, Swindon SN2 1UH, UK (E.K.). Institute of Biotechnology, Viikinkaari 1, 00014 University of Helsinki, Finland (S.B.), ,

**Keywords:** Comparative genomics, Genome duplication

## Abstract

Finding causal relationships between genotypic and phenotypic variation is a key focus of evolutionary biology, human genetics and plant breeding. To identify genome-wide patterns underlying trait diversity, we assembled a high-quality reference genome of *Cardamine hirsuta*, a close relative of the model plant *Arabidopsis thaliana*. We combined comparative genome and transcriptome analyses with the experimental tools available in *C. hirsuta* to investigate gene function and phenotypic diversification. Our findings highlight the prevalent role of transcription factors and tandem gene duplications in morphological evolution. We identified a specific role for the transcriptional regulators *PLETHORA5*/*7* in shaping leaf diversity and link tandem gene duplication with differential gene expression in the explosive seed pod of *C. hirsuta.* Our work highlights the value of comparative approaches in genetically tractable species to understand the genetic basis for evolutionary change.

**Supplementary information:**

The online version of this article (doi:10.1038/nplants.2016.167) contains supplementary material, which is available to authorized users.

## Abstract (web summary)

By assembling the genome of *Cardamine hirsuta* and conducting comparative genomic and transcriptomic analyses, a study reveals the prevalent role of transcription factors and gene duplication in morphological evolution.

**Supplementary information:**

The online version of this article (doi:10.1038/nplants.2016.167) contains supplementary material, which is available to authorized users.

Parallel genetic studies in *C. hirsuta* and the related model *A. thaliana* have provided a powerful platform to identify the molecular causes of trait diversity between these species at a gene-by-gene level^1–4^. In particular, leaf shape differences have provided an attractive model to investigate the genetic basis for morphological evolution^1–5^. To extend this approach to a genome-wide level and broaden its scope, we constructed a high-quality reference genome of the *C*. *hirsuta* strain ‘Oxford’^2^ for comparison with *A. thaliana*^6^. *C. hirsuta* and *A. thaliana* belong to lineage I in the Brassicaceae family^7,8^, together with *A. lyrata*^9^ and *Capsella rubella*^10^, all of which have fully assembled genomes (Fig. 1a). Complete or partial genome sequences are available for a number of other Brassicaceae species^6,9–14^, including *Aethionema arabicum*^11^ in the earliest diverging lineage of Brassicaceae (Fig. 1a). This allows comparisons between *C. hirsuta* and *A. thaliana* to gain additional context from comparative analyses within lineage I and the Brassicaceae family as a whole.

**Figure 1:**
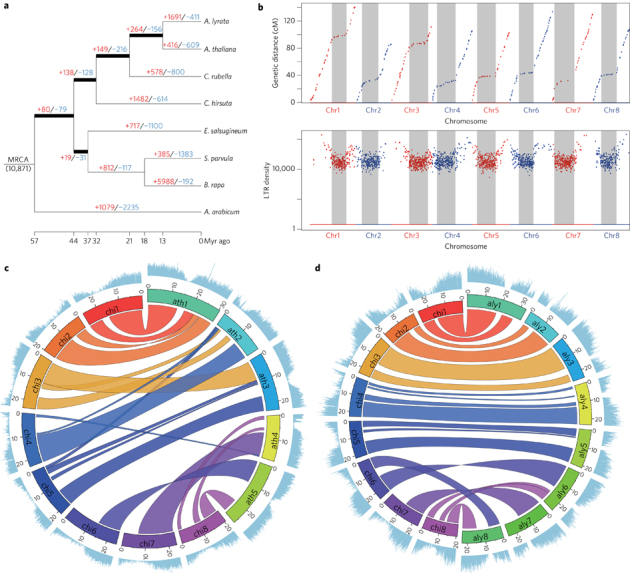
*C. hirsuta* genome. **a**, Phylogenetic tree for *A. thaliana*, *A. lyrata*, *C. rubella*, *C. hirsuta*, *E. salsugineum*, *S. parvula*, *B. rapa* and *A. arabicum* constructed using 10,111 orthologous genes within these eight species. Bold branches have maximum confidence^35^. The number of gene families expanded (red) or contracted (blue) compared with the most recent ancestor common ancestor (MRCA) are indicated along each branch. **b**, The upper panel shows the position of genetic markers mapped to the *C. hirsuta* genome assembly; the *y*-axis shows the genetic distance; shaded regions are inferred centromeric or pericentromeric heterochromatic regions that show very rare recombinations. The lower panel shows a rainforest plot of long terminal repeat (LTR) genes in the *C*. *hirsuta* genome; the *y*-axis shows the minimum distance of each LTR gene to its neighbours in a logarithmic scale. Chromosomes are indicated on the *x*-axis. **c**,**d**, Circos plots showing synteny between the genomes of *A. thaliana* (ath) and *C. hirsuta* (chi) (**c**), and *A. lyrata* (aly) and *C. hirsuta* (chi) (**d**); the outer circle shows the gene density distribution with a window size of 100 kbp.

To sequence *C. hirsuta*, we used a shotgun sequencing strategy, combining paired end reads (197× assembled sequence coverage) and mate pair reads (66× assembled) from Illumina HiSeq (a total of 52 Gbp raw reads; see Supplementary Table 1). The short reads were first assembled with SOAPdenovo^15^ to generate contigs, which were further linked into superscaffolds using a custom Bayesian framework-based algorithm, which is a unified platform for genome assembly utilizing the mapping quality of the paired reads (BAMLINK; see Supplementary Methods). The superscaffolds were anchored and oriented with a further 8,249 bacterial artificial chromosome paired end sequences (6 Mbp physical coverage) and a genetic map with 328 markers, to obtain eight pseudomolecules of a total length of 183 Mbp (92.2% of the assembly, corresponding to the *C. hirsuta* chromosomes) and 614 unanchored fragments. The final assembly encompasses 198 Mbp, which is comparable to a previous flow cytometry estimate of 225 Mbp^16^. We demonstrated the high quality of the assembly using three independent datasets: by perfectly aligning 358 randomly selected, Sanger-sequenced regions (size 500–600 bp) to our assembly (Supplementary Table 2); by mapping a total of 1 Mbp of sequence derived from eight 454 shotgun sequence-assembled fragments and two Sanger-sequenced fragments with 99.98% identity to our assembly (Supplementary Fig. 1); and finally, we confirmed the co-linearity between the physical and genetic position of 328 genetic markers and 36 additional markers which were not used in the assembly procedure (Supplementary Methods; Fig. 1b, top, and Supplementary Fig. 2). Our results demonstrate that BAMLINK provides an efficient and accurate method to merge local information from different sequencing platforms with broad scale information from a genetic map.

We annotated the genome by a combination of *ab initio* gene prediction using Illumina transcriptome data collected from a range of tissues and heterologous homology evidence. A total of 29,458 protein-coding genes with 37,997 transcripts and 579 nuclear encoded tRNA were predicted in the *C. hirsuta* genome (Supplementary Table 3). We built a phylogeny based on the complete set of protein-coding genes for *C. hirsuta*, *C. rubella*, *A. thaliana*, *A. lyrata*, *A. arabicum*, *Brassica rapa*^12^, *Schrenkiella parvula*^13^ and *Eutrema salsugineum*^14^. This dates the divergence of *C. hirsuta* and *A. thaliana* to around 32 Myr ago, which is within the range of previous estimates^17^ (Fig. 1a). The *C. hirsuta* genome is largely syntenic to the genomes of *A. thaliana* and *A*. *lyrata* (Fig. 1c,d and Supplementary Fig. 3). Gene-rich regions are mostly confined to the chromosome arms and all chromosomes have long centromeric and pericentromeric regions, which collectively account for approximately 40% of the genome (78.9 Mbp). We assembled these typically challenging chromosome regions using BAMLINK and found that they are highly enriched in long terminal repeat (LTR) retrotransposons and exhibit very low recombination frequencies (Fig. 1b). In contrast, the centromeric regions account for only 14 Mbp of the *A. thaliana* genome, thereby explaining the inflated genome size of *C. hirsuta* compared with *A. thaliana.* A recent expansion of LTR retrotransposons also contributed to the increased genome size of *A*. *lyrata* compared to *A. thaliana*^9^ (Supplementary Fig. 4). However, whereas *A*. *lyrata* LTR retrotransposons are relatively young (∼0.8 Myr) and broadly distributed throughout the genome, *C*. *hirsuta* LTR retrotransposons are older (median age ∼4.8 Myr) (Supplementary Fig. 4). The centromeres of other sequenced Brassicaceae are similarly large^9,10^, indicating that the *C*. *hirsuta* genome retains more ancestral features, including karyotype^18^ and genome size, than *A. thaliana*. Predominant selfing in *C. hirsuta*^18^ is associated with loss of gene function at the self-incompatibility *S* locus (Supplementary Figs 5 and 6). The *S* locus in *C. hirsuta* is syntenic with other Brassicaceae genomes, but the distinct *S* locus that evolved secondarily in the closely related genus *Leavenworthia*^19^ did not exist in *C. hirsuta* (Supplementary Fig. 7). Self-compatibility probably evolved recently in *C. hirsuta* as the *S* locus maintains hallmarks of functional *S* haplotypes despite disruptive mutations in the *SRK* and *SCR* genes^20^ (Supplementary Fig. 5).

To identify species-specific gene families that might contribute to trait diversification, we clustered the annotated protein-coding genes of *C. hirsuta*, *C. rubella*, *A. thaliana*, *A. lyrata*, *A. arabicum*, *B. rapa*, *S. parvula* and *E. salsugineum*. We identified 10,871 core gene families comprising at least one gene from each species, and determined expansion and contraction of gene families in different evolutionary lineages (Fig. 1a and Supplementary Methods). A five-way comparison of four lineage I species, *C. hirsuta*, *C. rubella*, *A. thaliana* and *A. lyrata*, with additional species distributed across the Brassicaceae, *E. salsugineum*, *S. parvula*, *B. rapa* and *A. arabicum*, shows that *C. hirsuta* has 694 unique gene families (Fig. 2a). We identified 5,560 genes in 2,067 families in *C. hirsuta* as tandem duplicates, and 16 of these families were specific to *C. hirsuta* (Supplementary Fig. 8). Among the total number of gene families in common to all eight species, 53 were identified as significantly expanded in *C. hirsuta* based on a phylogenetically informed test^21^ (*P *≤ 0.05) (Fig. 2b and Supplementary Fig. 9). Analysis of these expanded or unique gene families in *C. hirsuta* revealed an overrepresentation of transcription factor function (adjusted *P* = 2 × 10^−5^ for GO:0010468) (see Supplementary Tables 4–6 for enriched InterPro terms). Previous genetic studies have shown that transcription factors and tandem gene duplication contribute to morphological differences between *C. hirsuta* and *A. thaliana* leaves^1–3^. To test the significance of this observation genome wide, in an unbiased way, we identified differentially expressed genes (DEGs) between *C. hirsuta* and *A. thaliana* during early leaf development. We found a significant overrepresentation of both transcription factors (*P* = 1.9 × 10^−4^) and tandemly duplicated genes (*P* = 2.07 × 10^−46^) among these DEGs, indicating that these gene types are prevalent in the species-specific leaf transcriptomes.

**Figure 2:**
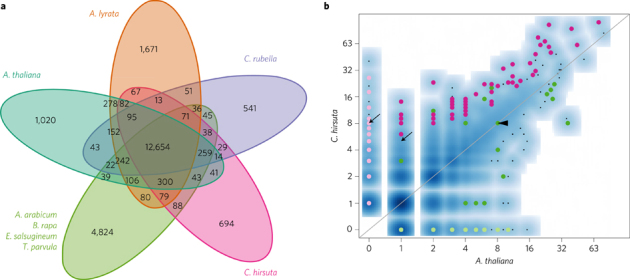
Species-specific expansion and contraction of gene families. **a**, Venn diagram comparing the number of gene families shared between four lineage I species, *A. lyrata*, *C. hirsuta*, *A*. *thaliana* and *C*. *rubella*, and four additional species distributed across the Brassicaceae, *E. salsugineum*, *S. parvula*, *B. rapa* and *A. arabicum*. **b**, Logarithmically scaled smooth scatterplot of gene families showing the number of species-specific members in *A*. *thaliana* (*x*-axis) and *C*. *hirsuta* (y-axis). Dots above the grey line represent gene families that are significantly expanded in *C. hirsuta* (pink) or contracted in *A*. *thaliana* (green), and dots below the grey line represent gene families that are expanded in *A*. *thaliana* (green), based on Hahn's test with eight species; pale pink dots represent families that are unique to *C. hirsuta* and pale green dots represent families that are unique to *A*. *thaliana*. The arrows indicate two families containing pectin methylesterase (PME) and PME inhibitor genes; one gene family has no members in *A. thaliana*. The arrowhead indicates the gene family that contains *PLETHORA*5/7 transcription factors (note this family does not correspond to the green dot and is not significantly expanded or contracted).

We used these transcriptome data to investigate the molecular causes of leaf shape diversity between *C. hirsuta* and *A. thaliana* in more depth. Following the premise that co-option of gene networks active in the shoot apical meristem contributes to leaf shape diversity^1^, we found 278 meristem genes^22^ upregulated in *C. hirsuta* relative to *A. thaliana* during early leaf development (fold change ≥2.0 times greater in *C. hirsuta* than in *A. thaliana*). Transcription factors were significantly enriched (*P* ≤ 0.05) among these upregulated meristem genes and comprised 44 genes including *SHOOT MERISTEMLESS*, *BREVIPEDICELLUS* and *CUP-SHAPED COTYLEDON1*, which were previously implicated in dissected leaf development^2,3^ (Fig. 3a, Supplementary Tables 7 and 8). These enriched transcription factors included the *C. hirsuta* orthologues of *PLETHORA5* (*PLT5*) and *PLT7*, which are involved in meristem stem cell specification but have not been previously implicated in leaf diversity^23^. *ChPLT5* and *ChPLT7* are upregulated in *C. hirsuta* leaves relative to *A. thaliana* and their transcripts accumulate at the sites of emerging leaflets (Fig. 3b,c and Supplementary Fig. 10). We reduced *ChPLT*5/7 expression in *C. hirsuta* leaves by means of an artificial miRNA that targeted both genes and found a pronounced reduction in the number of leaflets formed per leaf (Fig. 3d–h and Supplementary Fig. 10). Moreover, expressing *ChPLT7* in the simple leaf margin of *A. thaliana* under the *CUC2* promoter was sufficient to cause ectopic leaflet formation (Fig. 3i,j). Therefore, *ChPLT5/7* are necessary and ChPLT7 is sufficient for leaflet formation. Since *PLT7* coding sequences from both *C. hirsuta* and *A. thaliana* were sufficient to cause leaflet production in *A. thaliana* (Fig. 3i,j and Supplementary Fig. 10), it is likely that regulatory sequence differences in *PLT7* contributed to leaf shape divergence between these species.

**Figure 3:**
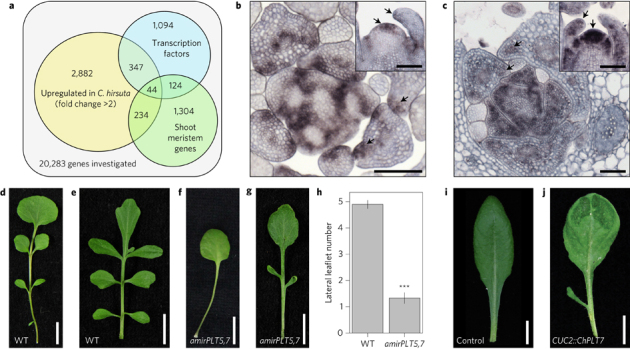
PLT5/7 transcription factors control species-specific leaf shape. **a**, Venn diagram showing the intersection of shoot meristem-expressed genes and transcription factor-encoding genes that are expressed more than twofold higher in *C. hirsuta* than in *A. thaliana* leaves. **b**,**c**, *In situ* hybridization of *ChPLT5* (**b**) and *ChPLT7* (**c**) expression in transverse and longitudinal (shown in inset) sections through *C. hirsuta* shoot apices. Arrows indicate signal in the shoot apical meristem and leaflets. Scale bars, 100 µm. **d**–**g**, Rosette leaf 5 (**d**,**f**) and cauline leaves (**e**,**g**) of *C. hirsuta* wild type (WT) (**d**,**e**) and *35S*::*amirChPLT5,7* (**f**,**g**). **h**, Quantification of leaflet number in rosette leaf 5 of wild type and *35S*::*amirChPLT5,7.* Error bars show standard error of leaflet number. ***, regression of leaflet number on genotype (*P* < 0.001). **i**,**j**, Representative rosette leaves of *A. thaliana* vector-only control (**i**) and *CUC2*::*ChPLT7* (**j**). *N* = 20/20 (**i**) and 4/27 (**j**) independent transgenic lines with similar phenotypes. Scale bars, 1 cm.

To exploit comparisons between *C. hirsuta* and *A. thaliana* more broadly, we determined DEGs during seed pod development in each species. Seeds are dispersed by explosive pod shatter in *C. hirsuta*; a trait that readily distinguishes it from *A. thaliana* and other species in the Brassicaceae family^24^. We found a significant overrepresentation of transcription factors (*P* = 2.6 × 10^−8^ for *C*. *hirsuta* and *P* = 1.2 × 10^−4^ for *A. thaliana*) and tandemly duplicated genes (*P* = 8.6 × 10^−4^ for *C. hirsuta* and *P* = 1.0 × 10^−15^ for *A. thaliana*) in both species. Among 319 orthologous genes that were differentially expressed only in *C. hirsuta* (adjusted *P* < 0.05 in *C. hirsuta*, adjusted *P* > 0.3 in *A. thaliana*), we found six highly enriched gene ontology (GO) terms related to cell wall and pectinesterase activity (Fig. 4a). The six GO term enrichments were largely attributed to ten genes encoding pectin methylesterases (PMEs) and PME inhibitors (PMEIs) (Supplementary Table 9). To investigate whether species-specific *PME/I* genes were differentially expressed in *C. hirsuta* seed pods, we identified two expanded *PME/I* families, which together contained eight upregulated genes (Fig. 2b, Supplementary Fig. 11 and Supplementary Table 9). A total of five DEGs within these expanded *PME/I* families were tandem duplicates present in *C. hirsuta* but not in *A. thaliana* or other sequenced genomes that we analysed in the Brassicaceae (Fig. 4b–d, Supplementary Figs 11 and 12). Three upregulated *PMEI* genes were highly expressed in *C. hirsuta* seeds, which had lower PME enzymatic activity per unit protein than *A. thaliana* seeds, and accumulated pectin with a high degree of methyl-esterification in asymmetrically thickened cell walls in the seed coat (Fig. 4e–h and Supplementary Fig. 13). Thus, our results provide an avenue to explore cell wall properties that distinguish the seeds and pods of *C. hirsuta* from *A. thaliana*.

**Figure 4:**
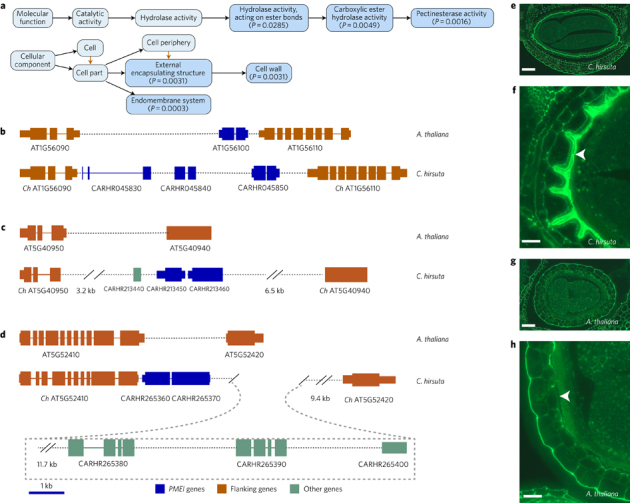
Tandemly duplicated genes contribute to gene expression differences that distinguish the explosive seed pod of *C. hirsuta*. **a**, GO terms that are enriched (dark blue) in the set of 319 DEGs specific to *C*. *hirsuta* and not *A. thaliana* seed pod development, and their parental terms (light blue). *P* values were obtained from exact Fisher tests after correcting for multiple hypothesis testing. Black arrows indicate an ‘is a’ and orange arrows indicate a ‘part of’ relationship between respective terms in the GO graph. **b**–**d**, Syntenic blocks in *A. thaliana* and *C. hirsuta* indicate that the *C. hirsuta* genes CARHR045830, CARHR045840 and CARHR045850 (**b**), CARHR213450 and CARHR213460 (**c**), and CARHR265360 and CARHR265370 (**d**) were derived by species-specific tandem duplication. **e**–**h**, Fluorescent signal of LM20 antibody labelling in seeds of *C. hirsuta* (**e**,**f**) and *A. thaliana* (**g**,**h**). Intense signal shows the asymmetrically thickened cell walls of the seed coat sub-epidermal layer in *C. hirsuta* (**e**,**f**), and the outer cell wall of the seed coat epidermis in *A. thaliana* (**g**,**h**); arrowheads indicate the difference in cell wall thickening of the sub-epidermal layer between *A. thaliana* and *C. hirsuta*. Scale bars, 100 µm (**e**), 20 µm (**f**,**h**), 50 µm (**g**).

Individual case studies have previously identified changes in transcription factors and tandemly duplicated genes as causes of morphological diversity in multicellular organisms^1,25–29^. Our results indicate that these are not isolated examples but rather that evolutionary changes in the expression of transcription factors and tandem gene duplicates may provide privileged molecular paths for the generation of diversity. For example, we identified previously unsuspected roles for PLT5/7 transcription factors and tandemly duplicated *PMEI* genes in divergent leaf and seed dispersal traits between *C. hirsuta* and *A. thaliana*. Notably, tandemly duplicated genes (including *PMEI*s) and transcription factors (including *PLT*s), as well as genes differentially expressed in a species-specific manner during fruit development and in young developing leaves, were enriched within gene families that showed evidence for positive selection (Supplementary Fig. 14), suggesting that some of these genes may have evolved non-neutrally to contribute to trait diversity. We found that these gene families under positive selection, together with expanded gene families and tandemly duplicated genes, have increased functional diversity as measured by the Shannon entropy of domain architecture (Supplementary Figs 15 and 16). However, tandemly duplicated genes show more domain conservation than expanded gene families (Kolmogorov–Smirnov test, *P* < 2.2 × 10^−16^). Taken together, these findings suggest that tandem gene duplication contributes to trait diversity while retaining stronger domain conservation than expanded gene families. This difference might reflect stronger evolutionary constraints^30^, gene conversion^31^ or simply younger age. Our study underscores how the comparison of high quality, annotated genomes and developmentally targeted transcriptomes between closely related species with high genetic tractability can establish causal links between genotypic and phenotypic variation above the species level^29^. This approach provides a valuable complement to linkage-based methods that rely on genetic crosses or association mapping^32,33^.

## Methods

### Plant material

#### DNA

*C*. *hirsuta* of the reference accession Oxford (Ox) (specimen voucher Hay 1 (OXF)^4^ was self-pollinated in the greenhouse for seven generations before being used for next generation sequence library preparation.

#### RNA

Leaf and fruit tissue was harvested from *A. thaliana* Col-0 and *C. hirsuta* Ox grown on soil in either a growth chamber under short day conditions (8 h light (20 °C) and 16 h dark (18 °C)) for leaves, or a greenhouse under long day conditions (16 h light (20 °C) and 8 h dark (16 °C)) for fruit. Total RNA from three biological replicates of microdissected young leaves (L5 and L6), or two biological replicates of whole fruits at two developmental stages (9 and 16), was isolated from each species using the RNeasy Plus Micro Kit (Qiagen) and reverse transcription carried out using the Superscript VILO cDNA Synthesis Kit (Life technologies).

### Genome assembly and annotation

The Illumina short reads were first assembled with SOAPdenovo^15^ to generate contigs. These contigs were further linked into superscaffolds using BAMLINK, which is a unified platform for genome assembly utilizing the paired reads and genetic map information (see Supplementary Methods). Initial gene models were derived as statistically combined consensus models from both *ab initio* gene predictions and homologous evidence (see Supplementary Methods). These predictions were adjusted by aligning *C. hirsuta* RNA-Seq data from seedling, leaf, floral and fruit tissues, using the cufflinks suite^34^, to retrieve alternative splicing models. Gene models were annotated for Interpro domains, GO terms and a description line using the AHRD pipeline (https://github.com/groupschoof/AHRD/), and gene models with transposon signatures were removed.

### Phylogenetic analysis

An ultrametric species tree of eight crucifers, *A. thaliana*, *A. lyrata*, *C. hirsuta*, *C. rubella*, *A. arabicum*, *B. rapa*, *E. salsugineum* and *S. parvula*, was generated from 10,111 concatenated multiple sequence alignments (MSA) of orthologous genes. This MSA was submitted to maximum likelihood phylogenetic reconstruction with FastTree v2.1.7^35^. The maximum likelihood tree was then rescaled into an ultrametric tree using a penalized likelihood approach.

### Quantification of gene expression

Paired-end reads were aligned to the reference genome (tair10 for *A. thaliana* and CHIV1 for *C. hirsuta*) using tophat with default parameters. Raw read counts per gene were quantified with HTSeq v0.5.4p1 (http://www-huber.embl.de/users/anders/HTSeq/) using the ‘–stranded = no –type = CDS’ option. To facilitate cross-species comparisons, reads within UTR regions were ignored since UTR regions are generally more divergent than CDS regions. Differential expression between samples from the same species was determined using DESeq. We found the most sensitive parameter settings for the function *estimateDispersions* were method = ‘blind’, and sharingMode = ‘fit-only’.

### Data availability

The assembled genome sequence and annotation, the raw Illumina genomic DNA reads and the Illumina RNA-seq reads are available from GenBank (Biosample: SAMN02183597; Bioproject: PRJNA293154) and from our website http://chi.mpipz.mpg.de/assembly. Source code of BAMLINK is available at http://chi.mpipz.mpg.de/software. The data that support the findings of this study are also available from the corresponding author on request.

## Supplementary information

Supplementary Information Supplementary Methods, Supplementary Tables 1-13 and Figures 1-18. (PDF 4305 kb)
