# Supplementary material for: The Cardamine hirsuta genome offers insight into the evolution of morphological diversity
Source: Nat Plants. 2016 Oct 31;2(11):16167. doi: 10.1038/nplants.2016.167 (PMC8826541; doi:10.1038/nplants.2016.167)
Supplement: Supplementary Information — Supplementary Methods, Supplementary Tables 1-13 and Figures 1-18. (PDF 4305 kb) [file 41477_2016_BFnplants2016167_MOESM277_ESM.pdf]

nature plants

SUPPLEMENTARY INFORMATION

ARTICLE NUMBER: 16167 | DOI: 10.1038/NPLANTS.2016.167

The *Cardamine hirsuta* genome offers insight into the evolution of morphological diversity

Xiangchao Gan[1*¶], Angela Hay[1*], Michiel Kwantes[1*], Georg Haberer[2], Asis Hallab[1], Raffaele Dello Ioio[1], Hugo Hofhuis[1], Bjorn Pieper[1], Maria Cartolano[1], Ulla Neumann[1], Lachezar A. Nikolov[1], Baoxing Song[1], Mohsen Hajheidari[1], Roman Briskine[3], Evangelia Kougioumoutzi[4], Daniela Vlad[4], Suvi Broholm[4], Jotun Hein[5], Khalid Meksem[6], David Lightfoot[6], Kentaro K. Shimizu[3], Rie Shimizu-Inatsugi[3], Martha Imprialou[5], David Kudrna[7], Rod Wing[7], Shusei Sato[4], Peter Huijser[1], Dmitry Filatov[4], Klaus F. X. Mayer[2], Richard Mott[8], and Miltos Tsiantis[1†]

[1] *Max Planck Institute for Plant Breeding Research, Carl-von-Linné-Weg 10, 50829 Köln, Germany*

[2] *Plant Genome and Systems Biology, Helmholtz Zentrum Munich, Ingolstädter Landstrasse 1, 85764 Neuherberg, Germany*

[3] *Department of Evolutionary Biology and Environmental Studies, University of Zurich, Winterthurerstrasse190, CH-8057 Zurich, Switzerland*

[4] *Department of Plant Sciences, University of Oxford, South Parks Rd, Oxford OX1 3RB, UK*

[5] *Department of Statistics, University of Oxford, 1 South Parks Road, Oxford, OX1 3TG, UK*

[6] *Department of Plant, Soil and Agricultural Systems, Southern Illinois University, Carbondale, Illinois 62901, USA*

[7] *Arizona Genomics Institute, School of Plant Sciences and BIO5 Institute for Collaborative Research, University of Arizona, 1657 East Helen Street, Tucson, AZ 85721*

[8] *UCL Genetics Institute, University College London, Gower Street, London WC1E 6BT, UK*

[*] *These authors contributed equally to the work*

[¶] Current address: The Global Food Security, BBSRC, Polaris House, North Star Avenue, Swindon, SN2 1UH, UK

[†] Authors for correspondence: e-mail address (gan@mpipz.mpg.de; tsiantis@mpipz.mpg.de) phone: +49 (221) 5062105, fax: +49 (221) 5062674

© 2016 Macmillan Publishers Limited, part of Springer Nature. All rights reserved.

1 PORTAL FOR ACCESSING THE DATA AND ANALYSES

Web site: The assembled genome sequence and annotation, the raw Illumina genomic DNA reads and the Illumina RNA-seq reads are available from GenBank (Biosample:SAMN02183597; Bioproject:PRJNA293154) and from our web site http://chi.mpipz.mpg.de/assembly. The aligned BAM files of genomic and transcript reads are available from our web site.

GBROWSE genome browser We created a public GBROWSE tool http://chi.mpipz.mpg.de/gbrowse containing all the assembled genome sequence, mapped RNA-seq data and annotations generated by this project and some previous projects.

2 GENOME SEQUENCING OF *C. HIRSUTA* GENOME

2.1 Choice of biological material

C. hirsuta of the reference accession Oxford (Ox) (specimen voucher Hay 1 (OXF)[4] was self-pollinated in the greenhouse for 7 generations prior to being used for next generation sequence library preparation.

2.2 Illumina shotgun sequencing

We shotgun sequenced the genome of *C. hirsuta* reference line Ox to 291× coverage using a combination of platforms (Supplementary Table 1). A paired-end library of 450bp and 2 mate pair libraries were prepared using Illumina's paired-end and mate-pair kits respectively. 5ug of DNA was sheared with a Covaris S2 ultrasonicator for the paired-end library and 10ug of DNA was sheared with a Hydroshear for the mate-pair libraries. The paired-end library was sequenced on two 100bp runs on a Hiseq 2000 sequencer, yielding approximately 39Gb of sequence data. The two mate-pair libraries were barcoded separately and sequenced as 90bp reads on one lane of a flow cell, yielding ~6.9Gb and ~6Gb of sequence data respectively.

2.3 Sanger sequencing of BAC clone ends

A bacterial artificial chromosome (BAC) library was constructed by the Arizona Genomics Institute, University of Arizona, Tucson, AZ 85721, USA, with large genomic DNA inserts of *C. hirsuta* Ox in the vector pAGIBAC1, which is a modification of pIndigoBAC5 by the addition of a SwaI site, according to the following protocol[34]. The library was plated in 384-well plates and clones were Sanger sequenced at the Kazuza DNA Research Institute, Japan, to produce ~500bp of double stranded sequence from each clone end.

2.4 Genetic map construction by whole genome profiling

A previously published genetic map containing 288 markers[1] was used as a guide for this new map design. A pilot run using our scaffolding algorithm with these markers showed that 29 large scaffolds (>100kbp) in our test assembly failed to map to the 8 linkage groups of the previous genetic map and thus can't be properly anchored to a chromosome with this set of markers. We visually inspected the genome for the presence of polymorphisms between the reference line Ox and a polymorphic accession Washington (Wa)[18] using the Integrative

Genomics Viewer[35]. In total, 226 SNPs were selected in the 5' or 3' ends of the scaffolds (between 4 and 8 SNPs per scaffold) such that the physical distance between them was maximized with respect to the total length of the scaffold. Several iterations of Sequenom assay designs were performed with the selected SNPs (Welcome Trust Center for Human Genetics, High Throughput Genomics, Oxford, UK) until 2 multiplexes (72 markers) were selected where each scaffold was represented by at least 1 SNP. The markers were then used in Sequenom assays to genotype 178 F8 Recombinant Inbred Lines (RILs) derived from a cross between the Ox and Wa, together with both founder accessions.

Genetic maps were constructed with joinmap v4.0 using genotyping data from the 72 new markers in 178 RILs. During several rounds of map optimization, isolated markers under strong segregation distortion and markers for which the nearest neighbour fit indicated inaccurate mapping were excluded. Where multiple markers mapped to the same genetic position on the same scaffold, only the marker with the highest quality genotyping data was retained. 54 markers survived this stringent selection and were located on the 29 previously unmapped scaffolds, with 25 scaffolds represented by at least 2 markers. Together with the previously reported 288 markers, a total of 328 markers were used for scaffolding. The final genetic map used for this study is shown in Supplementary Fig. 2.

3 DE NOVO GENOME ASSEMBLY AND MAPPING OF *C. HIRSUTA*

3.1 Overview

To assemble the genome, we developed a novel scaffolding algorithm dubbed BAMLINK (software available from the project website). It generates links between pre-assembled contigs by processing the short read alignments, or other *a priori* information, to guide the scaffolding process in a Bayesian Framework. Here, two main types of *a priori* knowledge can be used. First, BAMLINK infers the distribution of insert sizes, based on the quality of alignment of reads to the pre-assembled contigs. Second, and optional, *a priori* information can include, for example, knowledge about the chromosomes on which contigs are found. Generally, BAMLINK can make use of any kind of prior knowledge. In the *C. hirsuta* genome project both types of *a priori* information were used.

BAMLINK is similar to the widely used scaffolding algorithm SSPACE in that both generate links between pre-assembled contigs by processing the short read alignment to guide the scaffolding process (Supplementary Fig. 18). However, BAMLINK is based on the widely used BAM (binary version of SAM) format, which allows users to make use of any aligner.

There are two main advantages of this read alignment based scaffolding method. First, considering whole reads instead of just *k*-mer subsequences on which De Bruijn Graph methods are based, enables BAMLINK to overcome mapping ambiguities that the latter cannot resolve. In this project, we applied BAMLINK to pre-assembled contigs generated by the SOAPdenovo v1.05[15] software suite. The second advantage stems from the combination of sequencing platforms employed in this project. It is well known that different sequencing platforms have unique systematic shortcomings. For example, the Illumina platform is prone to base calling errors, while the 454 platform is known to introduce short deletions. Pooling data from different sequencing platforms into a de novo assembly, e.g. using SOAPdenovo, often produces worse results than

single platform based assemblies. In contrast, BAMLINK produces very robust results in a multi-platform scenario because it focuses on the read/BAC-end pairs, which are uniquely aligned to the preliminary assembled contigs with high mapping quality (allowing mismatches). In the *C. hirsuta* genome project, all of the 454 mate-pair sequence data, along with BAC-end and genetic marker information, was successfully used for scaffolding by BAMLINK.

3.2 Preliminary *De Novo* assembly using Illumina short reads

Since Illumina sequence data contains base calling errors, it can dramatically increase memory usage during assembly and generate artifacts in the resulting assembly. We used the *k*-mer correction algorithm to process all 4 libraries of Illumina short reads according to the manual of SOAPdenovo v1.05. The following commands were used to perform the assembly:

- KmerFreq –q 0 –i correction.input –o correction
- Corrector –i correction.input –r correction.freq –t 4
- SOAPdenovo-127mer pregraph –s chi.config –K 79 –p 8 –o chi-k79
- SOAPdenovo-127mer contig –g chi-k79
- SOAPdenovo-127mer map –p 8 –s chi-k79 –g chi-k79
- SOAPdenovo-127mer scaff –g chi-k79

In fact, we used the short-insert (<1000bp) library and 3kbp insert-size library only, and discarded the 10kbp insert-size library after some cautious evaluation. The quality measurements before and after adding the 10kbp library justified our omission (Supplementary table 10). We think that the dramatic decrease in both maximum size of scaffolds and N50 values was due to chimeric sequences within the 10kbp library sequence data (Supplementary Fig. 17). However, the 10kbp library was used in subsequent scaffolding by BAMLINK, which as explained above, is more robust to sequence errors in the input data.

3.3 Removal of organellar contamination

Mitochondrial and chloroplast genomes are often highly represented in plant genome sequence data. To investigate the organellar contamination, we aligned all the assembled scaffolds to the mitochondrial and choloroplast genomes of *A. thaliana* using BLAT v.34[36].

We aligned the Illumina short-insert library reads to our preliminary assembly. The median coverage for all scaffolds is 198×, which is consistent with our estimate based on the size of the *C. hirsuta* genome. By only looking into the scaffolds from organellar DNA that are well conserved in both species (90% match), we found that the mitochondrial genome coverage was ~880× and the chloroplast genome coverage was ~7330×. We then investigated all the scaffolds that had ~880× or ~7330× coverage. As expected, we observed very high synteny between the organellar genomes of *A. thaliana* and those of *C. hirsuta*.

Based on our observation, we filtered out all the scaffolds with 60% match to the organellar genomes of *A. thaliana*. The remaining *C. hirsuta* scaffolds were merged with the mitochondrial and chloroplast genomes of *A. thaliana* to generate an intermediate assembly for further analysis. Assembly of the organellar genomes of *C. hirsuta* was accomplished using IMR/DENOM v0.4.0[32] by combining the iterative reads, mapping information and de novo assembly in the subsequent analysis.

3.4 Scaffolding pre-assembled contigs using BAMLINK

3.4.1 Alignment based Scaffolding for Illumina and 454 short reads using BAMLINK

After removing organellar contamination, short reads from libraries 1-4 were aligned to the intermediate assembly using the Burrows-Wheeler Algorithm (BWA). These alignments were used as input to BAMLINK, which in its initial phase considered only those read pairs where both ends mapped with a mapping quality score >=20. For these reads, the following measurements were assessed for each library separately: the median and median absolute deviation (MAD) of insert size for FR aligned reads, the median and MAD of insert size for RF aligned reads (for the mate-pair library) and finally the coverage for each pre-assembled contigs. The insert size plots for each library are shown in Supplementary Fig. 17. Here, the normal mate-pair reads have FR orientation, while the pairs that fail to cover both ends (anomalous pairs) of the PCR fragments have RF orientation, indicating that the noise ratio of the library can be easily quantified using the plot. We found that the 10kbp library has a high ratio of short-insert size pairs, thus justifying its omission from the SOAPdenovo preliminary assembly.

This data was used to link contigs into scaffolds as follows. First, read pairs where each end maps to a different contig were identified. Of these, we considered those read pairs that enabled a reliable linkage of contigs into scaffolds. Assuming that the insert size of a pair of reads follows a Gaussian distribution $N(L, \sigma^2)$, reliable pairs should map to *terminal* contig regions not larger than $L+3\sigma^2$. For mate-pair libraries, anomalous pairs often seriously affect the scaffolding performance, because they generate artificial links. BAMLINK discarded any read pairs from the scaffolding process that mapped to the terminal 450 bp regions of contigs (adjustable BAMLINK parameter). These discarded links only accounted for 14% and 3% of overall links in the 3kbp and 10kbp libraries respectively.

After filtering, the scaffolding was executed using a similar algorithm to that implemented in SSAKE or SSPACE. Briefly, a candidate link between contigs, starting with the longest, is accepted if enough read pairs (default k=5) support a scaffold. In the case that a contig is linked to more than one contig from the same end, a scaffold is accepted only if the ratio (r) of supporting read pairs between the best and second best link is higher than the threshold (r>1.5).

3.4.2 BAC end sequence alignment for scaffolding by BAMLINK

We aligned all the BAC end sequences from library 5 to the intermediate assembly, created above using smalt (v0.5.5). The BAM file created was used as input for BAMLINK to generate a new assembly.

3.4.3 The integration of genetic map by BAMLINK

In a subsequent step, the sequences for each genetic marker were aligned to the assembly. The target scaffolds, which had at least a single marker reliably aligned to them, were used to form 8 pseudo-chromosome pools according to the respective markers' chromosomes. Each pool contained about ~30 scaffolds and was stored as a single fasta file. We then aligned three libraries of Illumina short reads to the same assembly (section 3.4.2) to create a new alignment file in BAM format. BAMLINK was then used to establish scaffolds in the same way as explained in section 3.4.1 except that each time only one pseudo-chromosome fasta file was used as a template to build scaffolds. BAMLINK only counted those read pairs where both ends aligned to scaffolds within the template. As a consequence, the interference cross-chromosome links now disappeared. In practice, we found that we could assemble the whole chromosome reliably by only using the chromosome number information of each marker. Using the relative genetic position information of each marker was not necessary. In fact, we found that our final assembly was consistent with the *C. hirsuta* genetic map.

3.5 Correcting the Integrated Assembly

BAMLINK usually inscribes a series of "N" characters into the assembly whenever there is a gap between two pre-assembled contigs. To further polish our assembly, we used IMR/DENOM[32], which is a hybrid algorithm combining iterative reads mapping and de novo assembly. Apart from filling in the gaps, IMR/DENOM also identified short INDELs or SNPs that were caused by inaccurate scaffolding.

4 GENOME ANNOTATION

4.1 Gene Modeling

Initial gene models were derived as statistically combined consensus models from both *ab initio* gene predictions and homologous evidences. Cross-species homologies were derived from optimal spliced alignments using the Genomethreader software[37] with an *Arabidopsis thaliana* splice model and the complete representative protein sets of *Brassica rapa* (v1.1)[12], *A. thaliana* (TAIR10)[38], *A. lyrata* (v6)[9], tomato (v3.6)[39] and poplar (v2)[40] as well as *A. thaliana* (version PUT-169) and *B. napus* (version PUT-172) EST assemblies of the PlantsGDB resource[41]. To obtain training models for de novo gene finders, we aligned the *C. hirsuta* RNA-Seq data generated in this study to the genome sequence following the cufflinks protocol[42]. Protein coding potential of the merged cufflink transcripts was predicted applying the ORFpredictor webtool[43] complemented by a blastx comparison to the *A. thaliana* TAIR10 proteome. For gene models selected for training, we required a complete coding frame with start and stop codons, a minimum of 90% sequence identity and a maximum of 5% size difference to an *A. thaliana* homolog. Models were clustered by their identity and genomic overlap, and highly similar paralogous sequences with a pairwise identity of ≥95% were removed from clusters to achieve a non-redundant final training set of 8,649 loci. To train a gene finder, we randomly selected 2000 of

these loci with a maximum of 20% single exon genes. *Ab initio* predictions by upstream training was performed with the software tools Augustus[44], GeneID[45], GlimmerHMM[46] and Snap[47]. In addition, we applied Fgenesh+[48] using the supplied Dicot-specific matrix. To derive consensus gene models, the decision tree based algorithm of JIGSAW[49] was trained using the evidences above and statistically weighted gene models were predicted by a second run of JIGSAW. Lastly, we merged and adjusted these predictions with our alignments of the *C. hirsuta* RNA-Seq data from seedling, leaf, floral and fruit tissues, applying the cufflinks suite and thereby retrieved alternative splicing models based on the transcriptome data. The gene models were annotated for Interpro domains, GO terms and a description line using the AHRD pipeline (https://github.com/groupschoof/AHRD/), and gene models with transposon signatures were removed.

In total, 29,458 protein coding gene loci and 37,997 transcripts were identified in the final annotation set. This set deliberately includes 9,382 partial transcripts with a missing start and/or stop codon, which had support by homology and/or expression data. Key figures of the gene models are somewhat higher compared to the *A. lyrata* annotation but very close to the *A. thaliana* and *Capsella rubella* gene models (Supplementary Table 3). The slightly smaller CDS sizes are likely due to the inclusion of partial gene models in the *C. hirsuta* gene set.

Occurrence of tRNA genes was surveyed by the software tRNAscan-SE using default eukaryotic parameters. We detected a total of 579 tRNA loci in the nuclear genome encoding 20 amino acids, 561 regular tRNAs, 15 predicted pseudo-tRNAs, one suppressor and 3 tRNAs with undetermined specificity. 50 tRNAs contained an intron in their gene sequence.

4.2 Tandem Genes

Tandemly repeated genes are homologous or paralogous genes that are in close proximity in a genome. To identify tandem genes, an all-against-all BLASTp comparison of the representative proteins, i.e. omitting splice variants, was performed and only pairwise hits with an e-value $\leq 10^{-30}$ were retained. Next, we constructed an undirected graph with genes as nodes and edges between pairwise hits if not more than 9 unrelated genes separated the two genes of a hit pair in the genome. Tandem clusters were then simply retrieved as connected components of the graph. Because parameters and definitions of tandem genes vary widely between different genome projects, we applied this method to all four species (*A. thaliana, A. lyrata, C. rubella* and *C. hirsuta*) in order to obtain consistent results. The high colinearity of the four genomes enabled us to search for syntenic tandem clusters. Firstly, we computed the pairwise gene-based synteny of all species combinations applying the DAGchainer tool with a minimum blocksize of A=50 genes and a maximal distance D between matches of 100kbp. Next, we identified a total of 13,142 4× syntenic anchor points which represent reciprocal 1:1 syntenic relations between all four genomes and defined a syntenic grid across all four species. For each tandem cluster in species A, which was flanked by an up- and downstream anchor point, we searched for homologous genes (e-value$\leq 10^{-30}$) in the respective syntenic block of the other three species and included matches in an initial syntenic cluster. Because new matches are frequently tandem genes, and the corresponding tandem cluster in this species could extend the initial anchor points up- and/or downstream, we recursively adjusted the anchor points by searches in species B, C etc etc until no

extension of the block was observed or – in the case of small scaffolds – the 5'- or 3'- end of the scaffold was reached. This approach also identifies orthologous genes that are singletons or are completely deleted in one species but are tandemly repeated in one or more other species. The detected final numbers of tandem genes and clusters are shown in Supplementary Table 3 for each species.

5 COMPARATIVE GENOME ANALYSES

5.1 Repetitive and syntenic region analysis

RepeatMasker analyses. RepeatModeler was used for do novo identification of repetitive sequences in the genomes *C. hirsuta*, *A. lyrata*, *B. rapa*, *C. rubella*, *E. halophile,* and *S. parvula*. The classified repeats from all species were appended to the *A. thaliana* repeat library obtained from the Repbase database, resulting in a final library called Brassicaceae repeat library. In a final step this Brassicaceae repeat library was used with RepeatMasker (version open-4.0.3) to annotate transposable elements and other repetitive sequences in the *C. hirsuta* genome.

LTR retrotransposons. LTR retrotransposon were identified de novo for each genome using LTRharvest from the Genome Tools v1.5.1[50]. The identified LTR were then annotated using a two-step procedure to reduce false positives. First, LTRdigest[51] was used to identify candidate retrotransposons by searching for known protein domains from the Pfam protein family database. The remaining unknown candidates were then classified using BLAST with Repbase as the searched database. Annotated elements were then aligned using MUSCLE (v3.8.31)[52] and the Kimura two-parameter distance K was calculated for each alignment using the function distmat from EMBOSS v.6.6[53]. Finally, insertion times (T) were calculated using the method described in Hu *et al*[9] as T=K/(2r), with r as the rate of nucleotide substitution and r=7×10^{-9} used.

Identification of syntenic regions. The *C. hirsuta* genome was aligned to the *A. lyrata* and the *C. rubella* genomes with LASTZ (v1.02.00) program using a method described in[54]. In brief, any soft-masked *C. hirsuta* sequences larger than 10,010,000 bases were split into chunks of 10,010,000 bases overlapping by 10,000 bases for subsequent alignment. A similar process was followed for other genomes and then used for comparison, with chunks of 20,000,000 overlapping by 0. Next, the LASTZ software was used to align the chunks with parameters E=150, M=254, O=600, T=2 and Y=15000. Following alignment, the coordinates of the chunk were corrected and multiple alignments were chained together. The best-chained alignments are then used for comparative mapping and CIRCOS plots.

5.2 Gene family analysis

Gene families were identified by clustering all representative protein coding sequences (The longest isoform is used if multiple isoforms exist for a gene.) of the eight crucifers *A. thaliana*, *C. rubella*, *A. lyrata*, *C. hirsuta*, *A. arabicum*, *B. rapa*, *E. salsugineum* and *T. parvula*. This clustering was based on pairwise Bit-Scores from an "all vs all" BLAT v.34[36], which were used with Markov Clustering[55], as implemented in MCL v14-137 (with inflation parameter I=2.0), to identify distinct family clusters. The table for the distribution of gene family clusters in the eight crucifers is available at:
http://chi.mpipz.mpg.de/download/spe8_cluster_k2.0_table.txt.

Subsequently, we investigated which family clusters are expanded or

contracted within each of the above genomes. This was done by testing the null hypothesis, that the observed numbers of species-specific members were generated by the most likely background gene birth and death rate[56]. Finally, gene family clusters were cross-referenced with expression data in order to identify families with differentially expressed genes.

In order to characterize the obtained gene family clusters, the families were assigned short human readable descriptions (HRDs). These are based on the respective families' gene functions, obtained in the form of InterPro annotations. In cases where an InterPro annotation was shared by at least half a family's genes, this InterPro annotation was assigned as the family's HRD. Otherwise the most frequent InterPro annotation of any type was used to describe the gene family. The R code to generate HRDs for gene families can be obtained here: github.com/groupschoof/AHRD_on_gene_clusters.

5.3 Phylogenetic analysis

The ultrametric species tree of the eight crucifers *A. thaliana*, *A. lyrata*, *C. hirsuta*, *C. rubella*, *A. arabicum*, *B. rapa, E. salsugineum*, and *T. parvula* was generated from 10,111 concatenated multiple sequence alignments (MSA) of orthologous genes. Subsequently, this MSA was submitted to maximum likelihood phylogenetic reconstruction with FastTree v2.1.7[33,57,58]. The maximum likelihood tree was then rescaled into an ultrametric tree using a penalized likelihood approach[58,59] and the following minimum age estimates: *A. thaliana, A. lyrata* (13 MY), *A. lyrata*, *A. thaliana*, *C. rubella*, *C. hirsuta* (35.6 MY), *B. rapa*, *E. salsugineum* (38.4 MY), *A. thaliana*, *E. salsugineum* (43.2 MY), *A. arabicum*, *A. thaliana* (45 MY), and a maximum age estimate of 60 MY[14,60].

Several sets of homologous genes were submitted to phylogenetic reconstruction. Among others, the defined set of Pectin Methyl Esterases and their inhibitors (PMEIs), as well as the *PLETHORA* gene family. All sets of homologs were obtained by sequence similarity searches carried out with BLAT v.34[36]. The resulting sets of homologs were, in some cases, filtered in order to only retain members from *C. hirsuta* and *A. thaliana*. Subsequently, these protein sets were submitted to phylogenetic reconstruction. In the first step, a MSA was generated based on the chemical similarities of the respective amino acid residues using the program MAFFT v6.851b[61] (with automatic parameter settings "--auto"). This MSA was then filtered for conserved regions using the GBlocks v0.91b[62], set to allow a maximum of half of the positions within a conserved block to be gaps. Finally, this filtered MSA was submitted to maximum likelihood phylogenetic reconstruction with FastTree. In the case of "PMEI Family Two" the un-filtered MSA was used for maximum likelihood tree generation because filtering had discarded all alignment positions. Some trees were "midpoint-rooted" by setting the root of the tree exactly half way between the two most distant leaves.

5.4 Gene family-based positive selection tests

We scanned for the presence of positive selection for genes in the genomes of *A. thaliana*, *A. lyrata, C. hirsute, C. rubella*, *A. arabicum*, *B. rapa, E. salsugineum*, and *T. parvula* with a random effects model (FUBAR)[63], and an unrestricted branch-site random effects model (BUSTED)[64].The following procedure was applied to detect families with sites showing traces of positive selection (using FUBAR) and branches (genes) that were subject to positive

selection (using BUSTED), respectively. First, in order to decide which genes were eligible for a priori hypotheses of positive selection in BUSTED, Ka/Ks ratios were estimated for all orthologous gene pairs using the modified version of the Yang-Nielsen algorithm (MYN) as implemented in the KaKs_Calculator program version 1.2[65]. In the case of those genes for which no ortholog had been identified, Ka/Ks ratios were estimated using the closest related homolog of maximum nucleotide sequence similarity. Next, the amino acid (AA) sequences of the family genes were aligned based on the chemical properties of their respective amino acid residues. These multiple AA sequence alignments (MSA) were subsequently used to guide the alignments of the respective codon sequences (CDS). Then, the so obtained CDS alignments were used to generate maximum likelihood phylogenetic trees. Finally, the codon sequence alignments as well as the phylogenetic trees were fed into FUBAR to detect positively selected homologous codons (or amino acids), and into BUSTED to detect branches (genes) subject to positive selection. In the case of BUSTED all genes reported to be subject to positive selection by pairwise Ka/Ks ratio estimations were considered a priori hypothesis of positive selection. To correct for multiple hypothesis testing and obtain only high confidence FUBAR results, the maximum of all per family reported expected false positive rate (max-FPR) was collected from the FUBAR results. Subsequently, only those sites were retained where FUBAR had reported a posterior probability (PP) for positive selection ≥ 0.9 and where these PPs did not fall into the lower quantile defined by the max-FPR. Subsequently, the site-specific results obtained from BUSTED were corrected for multiple hypothesis testing[66] and only those were retained where the adjusted P-Values did not exceed 0.05. In a final step, genes found in families with positively selected homologous amino acids, identified by FUBAR, and genes found to be subject to positive selection as confirmed by BUSTED, were unified to obtain a set of "positively selected genes". These were then submitted to subsequent enrichment analyses.

6 *S*-LOCUS ANALYSIS

The evolution of predominant selfing by the loss of self-incompatibility is one of the most frequent evolutionary transitions in flowering plants[67]. In the self-incompatibility system of Brassicaceae, *SRK* and *SCR* genes at the *S*-locus confer female and male specificity, respectively[20,68,69]. Disruptive mutations in *SRK* or *SCR* have been shown to be responsible for most of the evolution of self-compatibility[69]. Because *C. hirsuta* is self-compatible, we surveyed the structure of the *S*-locus and identified disruptive mutations in *SRK* and *SCR*. Theory of sexual conflict has suggested that, unless mate limitation is extremely strong, *SCR* is likely to be disrupted in natural self-compatible populations while *SRK* may or may not be disrupted by secondary mutation[70-72]. The indel mutations in *ChSCR* as well as in *ChSRK* are consistent with this theory. Despite these disruptive mutations in *SRK* and *SCR*, typical hallmarks of a functional *S*-locus, including repetitive elements and full-length *SRK* and *SCR* genes, are maintained.

6.1 Reconstruction of *SRK* and *SCR* gene models

The *S*-locus in Brassicaceae is typically flanked by *PUB8/B80* and *ARK3* genes. To identify the location of the *S*-locus in *C. hirsuta*, the corresponding *A. thaliana* genes (*AT4G21350* and *AT4G21380* respectively) were aligned against

the assembled genome using NCBI BLAST (v2.2.29). BLAST hits suggested that the *S*-locus was located on the right arm of the chromosome 7 between positions 15,985,438 and 16,003,606. Transposable elements (TE) in the *S*-locus region (15,960,001..16,050,000 on chromosome 7) were identified separately with RepeatMasker v4.0.5 (http://www.repeatmasker.org/) using NCBI RMBLAST v2.2.27+ and Censor v4.2.29[73]. Both applications relied on Repbase Update 19.02[74]. Discovered TE sequences were converted to "nonexonpart" hints and supplied to AUGUSTUS (v3.0.1)[44] for re-annotating the region. In addition to the hints file, AUGUSTUS custom settings included a higher penalty for "nonexonpart" malus (1.15) and specific training annotation file (--species=arabidopsis). The application was able to reconstruct a *SRK* gene model but failed to find a *SCR* gene. Due to a frame-shift caused by a 13 bp deletion in exon 1, AUGUSTUS predicted a *SRK* model structure with an extra intron to skip the deletion, but the Sanger sequence (section 6.2) confirmed the typical 7-exon structure. To locate *SCR*, we searched the region for homology to the SCRL gene family sequence (PF06876) using HMMER v3.1b1[75]. The search uncovered only the second exon of the typical two-exon *SCR* gene structure. This second exon sequence was used as a query to BLAST against all Brassicaceae genes in GenBank. The first exon from the closest match with a complete gene model (GU723952; *A. halleri SCR* haplogroup A) was aligned to the *C. hirsuta* genomic sequence between *SCR* exon 2 and *PUB8/B80*. The alignment results pointed to the location of the first exon. The final model was manually adjusted to include splice sites. *C. hirsuta SCR* has only 7 out of 8 conserved cysteines and although this is not typical, it occasionally occurs in other species[76].

6.2 Validation of the exon structure and loss-of-function mutations in *SRK* and *SCR* sequences with ABI 3730 DNA analyzer

To confirm the genomic and cDNA sequences of *ChSCR* and *ChSRK* we used PCR amplification and Sanger sequencing. DNA was extracted from leaf tissue of the Ox accession using the DNeasy Plant mini kit (Qiagen). For cDNA analysis, RNA was extracted from young flower buds of the Ox accession using the RNeasy plant mini kit (Qiagen) and reverse-transcribed to cDNA using Super SMART (Clontech) in combination with Super Script III (Invitrogen). Primers used are listed in Supplementary Table 13. We amplified the genomic sequence of the *ChSCR* coding region (approx. 2300 bp, from 10 bp before the start codon to 70 bp after the stop codon) using primer pair SCR-F1 and R2 and sequenced using primers SCR-F1, F2, R1 and R2. The resulting sequence perfectly matched the assembled genome. We amplified the *ChSCR* coding region (approx. 420 bp) from cDNA template and sequenced using the same primer pair (SCR-F1 and R2). Both genomic and cDNA sequences confirmed the frameshift mutation in the second exon of *ChSCR*. We amplified the genomic sequence of the *ChSRK* gene using primer pair SRK-F1 and R1 (3547 bp) and sequenced exon 1 using primers SRK-F1 and F2 (approx. 960 bp, from 30 bp before the start codon to 200 bp before the end of exon 1), which included the 13-bp deletion causing a potential frame shift. The Sanger read (946 bp) perfectly matched the assembled genome. We amplified the *ChSRK* coding region from cDNA template using the same primer pair (SRK-F1 and R1) but could not obtain a visible band with gel electrophoresis, suggesting very low expression of *SRK,* possibly due to mRNA decay mediated by nonsense-mutation[77]. Nested PCR with primers SRK-F1 and R2 produced sufficient template for Sanger sequencing of a region from the

middle of exon 1 to the middle of exon 2 (approx. 950 bp) using primers SRK-F2, R2 and F5. Both genomic and cDNA sequences confirmed the 13-bp deletion causing a frame shift in *ChSRK*. Genbank accession numbers of these sequences are KT777652-KT777655.

6.3 Synteny Analysis

Genomic sequences for *A. thaliana* Col-0*, Capsella rubella*[10]*,* and *Leavanworthia alabamica* a4[11] were downloaded. The location of the *S*-locus in each species was identified by aligning the flanking genes (*DYT1, B70, PUB8/B80, ARK3* and *B120)* to available coding sequences using NCBI BLAST v2.2.30. Annotation of the *S*-locus and flanking genes in *A. lyrata* was provided by Goubet *et al.*[78]. Custom perl scripts were used to extract the features from the annotation files and to generate the synteny plot in Supplementary Fig. 5B.

6.4 Phylogenetic Analysis

A broad search for *SRK* genes was performed in GenBank (Supplementary Table 11). In addition, highly similar *ARK3* genes from *C. hirsuta* and several related species were added to make sure that the identified *SRK* sequence is not a mislabeled *ARK3* copy. After aligning the sequences with MUSCLE v3.8.31[52] using default parameters, a phylogenetic tree was constructed with MrBayes v3.2.2[79] using GTR model (nst=6) and gamma-shaped substitution rate variation (rates=invgamma). We ran the program for 1e+6 generations discarding 250,000 generations as “burn-in” and sampling every 5,000 generations. *SCR* gene sequences were retrieved from GenBank by searching for 300-2000 bp sequences described as *SCR/SP11* or cysteine-rich proteins located in the *S*-locus. The results were filtered manually to include only *SCR* genes where both exons were annotated. In addition, we used *SCR* sequences from distinct *S*-locus haplotypes in *A. lyrata* and *A. halleri*[78] as well as *SCR* sequences from the Cvi-0 allele and a restored sequence from the Col-0 allele[69] (Supplementary Table 12). A phylogenetic tree was constructed in the same way as for *SRK.*

7 QUANTIFICATION OF GENE EXPRESSION AND FUNCTION ANALYSIS

7.1 Quantification of Gene Expression

Paired-end reads were aligned to the reference genome (tair10 for *A. thaliana* and CHIV1 for *C. hirsuta*) using tophat with default parameters. Raw read counts per gene were quantified with HTSeq v0.5.4p1(http://www-huber.embl.de/users/anders/HTSeq/) using the "--stranded=no --type=CDS" option. To facilitate cross-species comparisons, the reads within UTR regions were not taken into account since UTR regions are generally more divergent than CDS regions.

Differential expression between samples from the same species was determined using DESeq[80]. We found the most sensitive parameter settings for the function *estimateDispersions* were method="blind", and sharingMode="fit-only"[81].

The identification of differential expression between samples from different species is often difficult due to gene deletions and duplications. In this study, we used very strict parameters to lower the false positive rate. First, a reciprocal gene sequence similarity search was executed using BLAT v.34[36]. Sequence pairs were

interpreted as orthologous when each gene had the respective other as its highest scoring hit. Using this criterion, 20,284 orthologous pairs were selected for subsequent analysis. Next, the library normalization of the gene expression raw counts for each biological duplicate was done gene by gene, using a script modified from DEseq code (available on request). After normalization, the expression level was evaluated and differential expression was determined as a fold-change between species that was larger than 2.0 or smaller than 0.5.

7.2 Protein Function Annotation, Gene Ontology, and InterPro analysis

C. hirsuta proteins were annotated with Human Readable Descriptions (HRD) and Gene Ontology (GO) terms using the AHRD program. The GO term annotations for *A. thaliana* genes were obtained from TAIR10. Here, all types, including electronic GO annotations, were used for *A. thaliana* as well as to extend the AHRD-based GO annotations for orthologous *C. hirsuta* genes. Conserved protein domains in the form of InterPro annotations were obtained from TAIR10 for *A. thaliana* genes, but prepared anew for *C. hirsuta* genes from the results of running InterProScan v5.0[82] on the *C. hirsuta* proteome. Subsequently, GO terms associated with InterPro domains were added to the GO annotations of *C. hirsuta* proteins. In a final step, all GO terms parental to terms already annotated were added to the respective *C. hirsuta* protein annotations.

A. thaliana genes were classified as transcription factors if they were annotated as such by PlantTFDB v3.0[83] or by TAIR10. In total, 1903 *A. thaliana* genes were classified as TF. *C. hirsuta* transcription factors were classified in three ways: first, any gene with an *A. thaliana* ortholog annotated as TF; second, any gene with a protein InterProScan annotated as TF; third, any gene with a GO term annotated as TF. In total, 1940 *C. hirsuta* genes were classified as TF.

Differentially expressed genes (DEG) were submitted to a function enrichment analysis of GO term annotations and conserved protein domain (InterPro) annotations. For the fruit samples, three DEG sets were identified: the set of genes differentially expressed both in *C. hirsuta* and *A. thaliana*, as well as the two sets of genes differentially expressed *only* in one species. For the leaf samples, DEGs were identified by the comparison of expression levels between *C. hirsuta* and *A. thaliana*. No genomic region distribution bias has been observed for all DEG datasets. Function enrichment tests were implemented as exact Fisher Tests for each distinct annotated GO term or InterPro domain. The background used for these tests was the full annotation set available for all genes whose expression levels were measured in both experiments. The alternative hypotheses tested were a significantly greater number of GO annotations or overrepresentation of InterPro domains in any of the DEG sets. For the exact Fisher Tests computed for GO term annotations, a correction was performed to discard counts of descendant terms that were already found to be enriched[84]. The resulting P-values were corrected for multiple hypothesis testing[66]. For the fruit samples, a filtering step was carried out in order to identify functions that were *only* enriched in those genes differentially expressed *uniquely* in *C. hirsuta*, but not in *A. thaliana* (adjusted $p < 0.05$ in *C. hirsuta*, adjusted $p > 0.3$ in *A. thaliana*). Enriched Gene Ontology (GO) terms were summarized using the web based REVIGO tool[85].

8 DEVELOPMENTAL GENETIC ANALYSIS

8.1 Leaf development and *PLT* analysis

8.1.1 Plant material and RNA extraction for RNAseq and real time quantitative PCR:

A. thaliana Col-0 and *C. hirsuta* Ox were grown on soil in a growth chamber under short day conditions [8 h light (20°C) and 16 h dark (18°C)]. Total RNA from 3 biological replicates of microdissected young leaves (L5 and L6) of each species was isolated using the RNeasy Plus Micro Kit (Qiagen) and reverse transcription carried out using the Superscript VILO cDNA Synthesis Kit (Life technologies). Primer pairs for PCR were designed such that the annealing sites in *A. thaliana* and *C. hirsuta* homologs were identical. qPCR reactions were performed using Power SYBRgreen Master Mix on a Viia7 Real-time PCR machine (Life Technologies). Expression of *PLT5/7* [*AtPLT5* (At5g57390), *ChPLT5* (CARHR271190), *AtPLT7* (At5g65510), *ChPLT7* (CARHR279490)] was normalized using the reference genes *TIP41* (At4g34270, CARHR242510), *ADAPTOR PROTEIN-2 MU-ADAPTIN* (*AP2M*, AT5G46630, CARHR174880) and *GLYCERALDEHYDE-3-PHOSPHATE DEHYDROGENASE C SUBUNIT 1* (*GAPC1*, AT3G04120, CARHR078250)[86]. Primer pair efficiencies were determined for both the *A. thaliana* and *C. hirsuta* amplicons from dilution curves on cDNA. Subsequently, relative quantities were calculated using the formula $1/E^{Cq}$ [87]. qPCR primer sequences are listed in Supplementary table 13.

8.1.2 CUC2::PLT5/7 construction:

A. thaliana and *C. hirsuta PLT5/7* coding sequences were amplified by PCR from cDNA and cloned into the intermediate vector pPLV25[88] and subsequently inserted as an XhoI/XmaI fragment into pBJ36AtCUC2[89]. Finally, the *CUC2::PLT5/7:OCS* expression cassettes were transferred as *Not*I fragments into the binary vector pMLBART. The constructs were transformed into *Agrobacterium* GV3101 using electroporation. *A. thaliana* plants were transformed by floral dipping[90] and seeds collected. Primary transformants were selected by spraying with BASTA and grown in long day conditions. Multiple independent transgenic lines with non-wild type leaf phenotypes were analyzed. Primer sequences are listed in Supplementary table 13.

8.1.3 35S::amirPLT5,7 construction, phenotypic analysis and qPCR:

An artificial microRNA targeting PLT5 and PLT7 (TATAGATGCGCTTCGTGTCTC) was designed using Web MicroRNA Designer (wmd3.weigelworld.org tool) and constructed by targeted mutagenesis of *mir319a* (pRS300, wmd3.weigelworld.org) using four primers (1-IV) listed in Supplementary table 13. This PCR amplicon was TA-cloned in the pGemT-easy vector (Promega©), sequenced and subcloned as a BamHI/EcoRI fragment into the pART7 vector. This expression cassette was then transferred as a NotI fragment into the pMLBART binary vector. This construct was transformed into *Agrobacterium* and *C. hirsuta* Ox plants, and selected with BASTA as described above. 10 out of 28 independent T1 lines showed the phenotype described in Fig. 3. Lateral leaflet number was quantified in rosette leaves of 19 wild type and 16 *35S::amirPLT5,7* T1 plants grown in two experiments. Regression analysis was used to test whether the transgene significantly affected leaflet number and to determine that the effect of the transgene did not depend on experiment. Mean leaflet number was then calculated for wild-type and transgenic plants across both experiments. The standard errors of the mean leaflet numbers were

calculated from the residuals after regressing leaflet number on experiment to remove variation between the experiments. *PLT5/7* mRNA levels were analysed in wild type and *35S::amirPLT5,7* transformants by qPCR. Total RNA from leaves of 2 biological replicates was isolated and reverse transcribed as described above. qRT-PCR reactions were performed in triplicate on the 2 independent RNA extractions using the SYBR-Green PCR Master Mix (Applied Biosystems) and an ABI PRISM 7300 Sequence Detection System (Applied Biosystems). Expression levels were normalized to the reference gene *GLYCERALDEHYDE-3-PHOSPHATE DEHYDROGENASE* (*GAPDH*) and relative expression was analyzed by regression. ΔCt was calculated for each technical replicate by subtracting the mean Ct of *GAPDH* for the respective biological replicate. The effect of the amir transgene on *PLT5,7* expression was tested by linear mixed effects regression analysis using the LME4 package in R. The variance among technical replicates of biological replicates was accounted for by a random term. The significance of the amir effect on *PLT5,7* expression was tested by likelihood ratio test. ΔΔCt and standard deviations were calculated according to manufacturer's instructions (Applied Biosystems) as average ΔCt of both biological replicates of wild type, subtracted from average ΔCt of both biological replicates of *amirPLT5,7*, to give a single fold-change value for the introduction of the amir transgene. Primer sequences are listed in Supplementary table 13.

8.2 Fruit development and *PMEI* analysis

8.2.1 *Plant material and RNA extraction for RNAseq:*

A. thaliana Col-0 and *C. hirsuta* Ox were grown on soil in a greenhouse under long day conditions [16 h light (20°C) and 8 h dark (16°C)]. Total RNA from 2 biological replicates of whole fruits at 2 developmental stages (9 and 16[91]) was isolated from each species using the RNeasy Plant mini kit (Qiagen) and DNaseI treated.

8.2.2 *Plant material and RNA extraction for real time quantitative PCR:*

C. hirsuta Ox was grown on soil in a greenhouse under long day conditions [16 h light (20°C) and 8 h dark (16°C)]. Total RNA from 3 biological replicates of whole fruits at stages 9, 15, 16 and 17, and tissue samples dissected from stage 17 fruits (seeds, valves and rest of the fruit) was isolated using the Spectrum Plant total RNA kit and On-Column DNaseI Digestion Set (Sigma). RNA integrity was examined using an Agilent 2100 Bioanalyzer II and first strand cDNA was synthesized in duplicate for the 3 biological replicates per sample using Superscript III reverse transcriptase and Oligo (dT) (Life Technologies). qPCR reactions were performed twice for each cDNA sample using Power SYBR Green Master Mix and a ViiA™ 7 Real-Time PCR System (Life Technologies). Expression levels were normalized to the reference gene *AP2M* (CARHR174880). Primer sequences are listed in Supplementary table 13.

8.2.3 *PME activity assay*:

Dry seeds (50-100 mg) of *A. thaliana* Col-0 and *35S::PMEI6*[92] and *C. hirsuta* Ox were ground in 250 μl of cold extraction buffer (4°C, 1M NaCl, 12.5 mM citric acid, 50mM Na_2HPO_4, pH 6.5 in dH_2O) with a motorized tissue grinder and left at 4°C for 4h. Samples were centrifuged at 14.2 k rpm for 15 minutes and supernatants were collected. Protein concentrations were determined by Bradford assay and 80 μl of extracts containing 15, 10, 5 and 2.5 μg of protein were loaded into 0.5 cm wells in a 1% agar plate supplemented with 0.1% of ≥85% esterified citrus fruit pectin (Sigma, cat. Nr. P9561), 50mM Na_2HPO_4 pH 6.5, and 12.5 mM citric acid. Plates were incubated overnight at room temperature and subsequently

stained with 500 μg/ml Ruthenium Red for 45 minutes. Background stain was reduced by destaining with dH_2O for 8 h and 48 h at room temperature and 4°C, respectively. Plates were imaged with a scanner, converted into gray scale (8bits) and inverted so that brighter signals had higher pixel intensity values. Average pixel intensity and staining area was quantified in ImageJ V 1.46R. Mean PME activity was calculated for each genotype from 3 biological and 2 technical replicates and error calculated as standard error of the mean. PME activity in *A. thaliana* wild-type seeds was set to 1 and the activity level of other genotypes were expressed relative to this.

8.2.4 *Immunocytochemistry and microscopy:*

A. thaliana Col-0 and *C. hirsuta* Ox fruits were cut in 2-3 mm segments with a scalpel and fixed in 4% paraformaldehyde and 0.5% glutaraldehyde in 0.1M sodium cacodylate buffer (pH 6.9, 4h at room temperature then overnight at 4°C)[93]. After thorough washing and dehydration in ethanol, the samples were embedded in medium-grade LR White resin (Plano GmbH, Wetzlar, Germany) for 6 d at room temperature, and then polymerized at 100°C for 90 min[94]. For bright field observation, 1 μm transverse sections were stained with 1% aqueous toluidine blue[95] supplemented with 1% sodium tetraborate, and mounted permanently in Entellan® (107960 Merck Millipore).

To detect methyl-esterified pectic homogalacturonan we used the LM20 monoclonal antibody (Plant Probes), which does not bind to un-esterified homogalacturonan[96]. Transverse 1 μm sections were dried down on diagnostic adhesion slides (Thermo Fisher Scientific X2XER202W# AD CE) and incubated overnight at 4 °C in 5% goat normal serum in TRIS buffer (20 mM TRIS, 225 mM NaCl, pH 6.9) supplemented with 1% (w/v) BSA (TRIS-BSA). After three washes for 10 min in TRIS-BSA, sections were incubated in a 1:50 dilution of LM20 for 1 h at room temperature, and then washed again in TRIS-BSA (3x 10 min). Subsequently, sections were incubated with a 1:20 dilution of goat anti-rat IgG secondary antibodies conjugated to Alexa Fluor 488® (Life Technologies, A-11006) for 1 h. As negative controls for immunocytochemical labelling, the primary antibody was replaced by TRIS-BSA. Sections were washed in TRIS-BSA (3x 10 min) and then mounted in anti-fade reagent Citifluor AF1 (Agar Scientific, UK). Slides were viewed on a Zeiss Axio imager.M2 compound microscope and images captured with an AxioCam HRc.

A. thaliana Col-0 and *C. hirsuta* Ox seeds were imaged with a Zeiss Supra 40VP scanning electron microscope after sputter-coating with palladium (Polaron Sputter Coater SC 7600, Quorum Technologies.)

Reference

33. Price, M.N., Dehal, P.S. & Arkin, A.P. FastTree 2--approximately maximum-likelihood trees for large alignments. *PLoS One* **5**, e9490 (2010).
34. Grotewold, E. *Plant functional genomics*, (Humana Press, Totowa, N.J., 2003).
35. Robinson, J.T. *et al.* Integrative genomics viewer. *Nat Biotechnol* **29**, 24-6 (2011).
36. Kent, W.J. BLAT--the BLAST-like alignment tool. *Genome Res* **12**, 656-64 (2002).
37. Gremme, G., Brendel, V., Sparks, M.E. & Kurtz, S. Engineering a software tool for gene structure prediction in higher organisms. *Information and Software Technology* **47**, 965-978 (2005).
38. Lamesch, P. *et al.* The Arabidopsis Information Resource (TAIR): improved gene annotation and new tools. *Nucleic Acids Res* **40**, D1202-10 (2012).

39. Tomato Genome, C. The tomato genome sequence provides insights into fleshy fruit evolution. *Nature* **485**, 635-41 (2012).
40. Tuskan, G.A. *et al.* The genome of black cottonwood, Populus trichocarpa (Torr. & Gray). *Science* **313**, 1596-604 (2006).
41. Dong, Q., Schlueter, S.D. & Brendel, V. PlantGDB, plant genome database and analysis tools. *Nucleic Acids Res* **32**, D354-9 (2004).
42. Trapnell, C. *et al.* Differential gene and transcript expression analysis of RNA-seq experiments with TopHat and Cufflinks. *Nat Protoc* **7**, 562-78 (2012).
43. Min, X.J., Butler, G., Storms, R. & Tsang, A. OrfPredictor: predicting protein-coding regions in EST-derived sequences. *Nucleic Acids Res* **33**, W677-80 (2005).
44. Keller, O., Kollmar, M., Stanke, M. & Waack, S. A novel hybrid gene prediction method employing protein multiple sequence alignments. *Bioinformatics* **27**, 757-63 (2011).
45. Blanco, E., Parra, G. & Guigo, R. Using geneid to identify genes. *Curr Protoc Bioinformatics* **4**, Unit 4.3. (2007).
46. Majoros, W.H., Pertea, M. & Salzberg, S.L. TigrScan and GlimmerHMM: two open source ab initio eukaryotic gene-finders. *Bioinformatics* **20**, 2878-9 (2004).
47. Korf, I. Gene finding in novel genomes. *BMC Bioinformatics* **5**, 59 (2004).
48. Solovyev, V., Kosarev, P., Seledsov, I. & Vorobyev, D. Automatic annotation of eukaryotic genes, pseudogenes and promoters. *Genome Biol* **7 Suppl 1**, S10 1-12 (2006).
49. Allen, J.E. & Salzberg, S.L. JIGSAW: integration of multiple sources of evidence for gene prediction. *Bioinformatics* **21**, 3596-603 (2005).
50. Ellinghaus, D., Kurtz, S. & Willhoeft, U. LTRharvest, an efficient and flexible software for de novo detection of LTR retrotransposons. *BMC Bioinformatics* **9**, 18 (2008).
51. Steinbiss, S., Willhoeft, U., Gremme, G. & Kurtz, S. Fine-grained annotation and classification of de novo predicted LTR retrotransposons. *Nucleic Acids Res* **37**, 7002-13 (2009).
52. Edgar, R.C. MUSCLE: multiple sequence alignment with high accuracy and high throughput. *Nucleic Acids Res* **32**, 1792-7 (2004).
53. Rice, P., Longden, I. & Bleasby, A. EMBOSS: the European Molecular Biology Open Software Suite. *Trends Genet* **16**, 276-7 (2000).
54. Schwartz, S. *et al.* Human-mouse alignments with BLASTZ. *Genome Res* **13**, 103-7 (2003).
55. Van Dongen, S. Graph clustering via a discrete uncoupling process. *SIAM Journal on Matrix Analysis and Applications* **30**, 121-141 (2008).
56. Hahn, M.W., De Bie, T., Stajich, J.E., Nguyen, C. & Cristianini, N. Estimating the tempo and mode of gene family evolution from comparative genomic data. *Genome Res* **15**, 1153-60 (2005).
57. Liu, K., Linder, C.R. & Warnow, T. RAxML and FastTree: comparing two methods for large-scale maximum likelihood phylogeny estimation. *PLoS One* **6**, e27731 (2011).
58. Paradis, E., Claude, J. & Strimmer, K. APE: Analyses of Phylogenetics and Evolution in R language. *Bioinformatics* **20**, 289-90 (2004).
59. Sanderson, M.J. Estimating absolute rates of molecular evolution and divergence times: a penalized likelihood approach. *Mol Biol Evol* **19**, 101-9 (2002).
60. Franzke, A., Koch, M.A. & Mummenhoff, K. Turnip Time Travels: Age Estimates in Brassicaceae. *Trends Plant Sci* (2016).
61. Katoh, K., Misawa, K., Kuma, K. & Miyata, T. MAFFT: a novel method for rapid multiple sequence alignment based on fast Fourier transform. *Nucleic Acids Res* **30**, 3059-66 (2002).
62. Talavera, G. & Castresana, J. Improvement of phylogenies after removing divergent and ambiguously aligned blocks from protein sequence alignments. *Syst Biol* **56**, 564-77 (2007).
63. Murrell, B. *et al.* FUBAR: a fast, unconstrained bayesian approximation for inferring selection. *Mol Biol Evol* **30**, 1196-205 (2013).
64. Murrell, B. *et al.* Gene-wide identification of episodic selection. *Mol Biol Evol* **32**, 1365-71 (2015).
65. Zhang, Z., Li, J. & Yu, J. Computing Ka and Ks with a consideration of unequal transitional substitutions. *BMC Evol Biol* **6**, 44 (2006).
66. Benjamini, Y. & Yekutieli, D. The control of the false discovery rate in multiple testing under dependency. 1165-1188 (2001).

67. Darwin, C. *The effects of cross and self fertilisation in the vegetable kingdom*, viii, 482 p. (John Murray, London, 1876).
68. Takayama, S. & Isogai, A. Self-incompatibility in plants. *Annu Rev Plant Biol* **56**, 467-89 (2005).
69. Tsuchimatsu, T. *et al.* Evolution of self-compatibility in Arabidopsis by a mutation in the male specificity gene. *Nature* **464**, 1342-6 (2010).
70. Tsuchimatsu, T. & Shimizu, K.K. Effects of pollen availability and the mutation bias on the fixation of mutations disabling the male specificity of self-incompatibility. *J Evol Biol* **26**, 2221-32 (2013).
71. Uyenoyama, M.K., Zhang, Y. & Newbigin, E. On the origin of self-incompatibility haplotypes: transition through self-compatible intermediates. *Genetics* **157**, 1805-17 (2001).
72. Tsuchimatsu, T., Kaiser, P., Yew, C.L., Bachelier, J.B. & Shimizu, K.K. Recent loss of self-incompatibility by degradation of the male component in allotetraploid Arabidopsis kamchatica. *PLoS Genet* **8**, e1002838 (2012).
73. Kohany, O., Gentles, A.J., Hankus, L. & Jurka, J. Annotation, submission and screening of repetitive elements in Repbase: RepbaseSubmitter and Censor. *BMC Bioinformatics* **7**, 474 (2006).
74. Jurka, J. *et al.* Repbase Update, a database of eukaryotic repetitive elements. *Cytogenet Genome Res* **110**, 462-7 (2005).
75. Mistry, J., Finn, R.D., Eddy, S.R., Bateman, A. & Punta, M. Challenges in homology search: HMMER3 and convergent evolution of coiled-coil regions. *Nucleic Acids Res* **41**, e121 (2013).
76. Watanabe, M. *et al.* Highly divergent sequences of the pollen self-incompatibility (S) gene in class-I S haplotypes of Brassica campestris (syn. rapa) L. *FEBS Lett* **473**, 139-44 (2000).
77. Maquat, L.E. Nonsense-mediated mRNA decay: splicing, translation and mRNP dynamics. *Nat Rev Mol Cell Biol* **5**, 89-99 (2004).
78. Goubet, P.M. *et al.* Contrasted patterns of molecular evolution in dominant and recessive self-incompatibility haplotypes in Arabidopsis. *PLoS Genet* **8**, e1002495 (2012).
79. Ronquist, F. *et al.* MrBayes 3.2: efficient Bayesian phylogenetic inference and model choice across a large model space. *Syst Biol* **61**, 539-42 (2012).
80. Anders, S. & Huber, W. Differential expression analysis for sequence count data. *Genome Biol* **11**, R106 (2010).
81. Mott, R. *et al.* The Architecture of Parent-of-Origin Effects in Mice. *Cell* **156**, 332-42 (2014).
82. Zdobnov, E.M. & Apweiler, R. InterProScan--an integration platform for the signature-recognition methods in InterPro. *Bioinformatics* **17**, 847-8 (2001).
83. Guo, A. *et al.* DATF: a database of Arabidopsis transcription factors. *Bioinformatics* **21**, 2568-9 (2005).
84. Falcon, S. & Gentleman, R. Using GOstats to test gene lists for GO term association. *Bioinformatics* **23**, 257-8 (2007).
85. Supek, F., Bosnjak, M., Skunca, N. & Smuc, T. REVIGO summarizes and visualizes long lists of gene ontology terms. *PLoS One* **6**, e21800 (2011).
86. Czechowski, T., Stitt, M., Altmann, T., Udvardi, M.K. & Scheible, W.R. Genome-wide identification and testing of superior reference genes for transcript normalization in Arabidopsis. *Plant Physiol* **139**, 5-17 (2005).
87. Pfaffl, M.W. A new mathematical model for relative quantification in real-time RT-PCR. *Nucleic Acids Res* **29**, e45 (2001).
88. De Rybel, B. *et al.* A versatile set of ligation-independent cloning vectors for functional studies in plants. *Plant Physiol* **156**, 1292-9 (2011).
89. Rast-Somssich, M.I. *et al.* Alternate wiring of a KNOXI genetic network underlies differences in leaf development of A. thaliana and C. hirsuta. *Genes Dev* **29**, 2391-404 (2015).
90. Clough, S.J. & Bent, A.F. Floral dip: a simplified method for Agrobacterium-mediated transformation of Arabidopsis thaliana. *Plant J* **16**, 735-43 (1998).
91. American Society of Plant Biologists. The Arabidopsis Book. (American Society of Plant Biologists., Rockville, MD).

92. Saez-Aguayo, S. *et al.* PECTIN METHYLESTERASE INHIBITOR6 promotes Arabidopsis mucilage release by limiting methylesterification of homogalacturonan in seed coat epidermal cells. *Plant Cell* **25**, 308-23 (2013).
93. Hawes, C.R. & Satiat-Jeunemaitre, B. Plant cell biology : a practical approach. in *Practical approach series* (Oxford University Press, Oxford, 2001).
94. McDonald, K.L. Out with the old and in with the new: rapid specimen preparation procedures for electron microscopy of sectioned biological material. *Protoplasma* **251**, 429-48 (2014).
95. O'Brien, T.P., Feder, N. & McCully, M.E. Polychromatic staining of plant cell walls by toluidine blue O. *Protoplasma* **59**, 368-373 (1964).
96. Verhertbruggen, Y., Marcus, S.E., Haeger, A., Ordaz-Ortiz, J.J. & Knox, J.P. An extended set of monoclonal antibodies to pectic homogalacturonan. *Carbohydr Res* **344**, 1858-62 (2009).

Supplementary Table 1. The raw sequence data used for *C. hirsuta* assembly.

Library ID	type	Platform	Centre	Insert size	Number of reads	read length (bp)	No. of bases	Coverage (X)
**library 1**	paired-end	ILLUMINA	WTCHG	450	390M	100	39G	196.98
**library 2**	mate pair	ILLUMINA	BGI	3000	76M	90	6.9G	34.70
**library 3**	mate pair	Roche 454	LIVERPOOL	3000	0.46M	[20, 622]	0.16G	0.82
**library 4**	mate pair	ILLUMINA	BGI	10000	69M	90	6G	31.66
**library 5**	BAC end	454	JPG	120K	8249		6M	
						**sum**	~52G	264

Supplementary Table 2. The 358 pairs of oligo-sequences used for PCR validation.

primer_left	primer_right	amplicon length	primer_left	primer_right	amplicon length
GCATGGGACCTTGAAAATTGCA	TAACAAATGCGCAATGACCAGT	801	ACACACTGGCTTAGATCACTGG	TCTTTCGTCCTGGGAAATTGGA	790
GTTATCGCCGCAAAACCTAGAC	GGACATCGGATTCTGATTTGCG	816	AACCTAATCGAACTGAACCGGT	CTAGACCCTAAAGCCGATCCAC	766
GCCATCATCACATGCTTCTTCC	CCGACAGTCCCAATTACGACAT	848	ACACTGTGACGTGTGCTAGATT	GATAGTCAGCGAAGTGGTCCAT	777
AATTCGCCATTTTCCTGAGCTG	TGGCGATTTTGAGTAGGGAACA	795	AAAAGTGATAGGGTGGGGATGG	CGGGAGAAGGAAAAGAGGACAA	888
GCTGCAACATAAGAGCCCTAGA	ATAGGGGATGCATCAGGTGAAC	791	CACAGATGCATAGAAATTCTTGGGT	AGCCTGCAATGAATTCAACACC	809
CAGAACGGGTTACAGATAGGCA	AACTCACCATCACTATGCACGT	823	AGAGATCACCGGAATAACGTCG	AGGGGATCTTAAGCAACAACAGA	864
CGTTATTGGTTGGTATGCGTGA	TGGCTTAGGGACCTTGAAAACA	755	GTCTTTCGGTCGTATTTATTGTCCA	CGTAAGGGTGCAGAACTAGAGT	782
TTCTGCAAGCTTTTCACAGAGC	TGCTGATGTACTTGTTGTGGTTG	848	GGCAGTAGTTGTACCTCTTTGC	ACCAAACTTTAGACACCGGGTT	793
TAATCTTCCGGCCGAAAGACAA	TACTGTCTCCGGAGCGTAAATG	801	CAGGACGGGACAGAATATGCTT	GGTTTTCTGGAAAGGTGTAACAGC	794
ACACCAAACTCAAATCCAAACACA	TCGCCTGGTGTATACGATATGC	847	GGCAATGACATACCGGAAACAC	GCTTATGGAACTCACAACCGC	805
ACGTAACAAAGAAGAAGATGTGGC	ATCCGTTGAAATCACCACACCA	773	ACCTTTGTCTTGCGTACTCCAT	CAATGCATGCAAGAGACTGGTT	854
CTCCTTCGTGTACTGGAAGCTT	CCATTTCGATGCCTGGGTAGTA	801	GAAAACACGACCGATTCTAGCG	AACTCCGATCACTCCAACCAAA	796
TGCCTCCCTTGGTTCATAACAA	CCTCCCTGACTCTACATGAAGC	770	CGCTATCGGGGATGATTCGTC	TGCTTCTTCGGTCTAAAGTCGT	753
GGTGGTGTCAAGTTTCAAACCC	AGGATGCTTGGGAATTAGACGT	751	CGCTGACAAACTTAAGACGTCG	TCACGGTTTGCCTCAGACATTA	773
AGGTTTAAAATATGACCCCTCGCT	CCCAACCATTCCCAAATCACAC	798	AGCTATCACAACCACCCAAAGT	GCACCTGTCAAGTACCATGGTA	835
ATCAGCAACAGCCTCATCGTAA	AAAGAAACTGACCTTGCCACCT	767	TTAGCAGGAGAAGAAGGAACCG	GTTTGTACTTGTGGGCGTTGTG	767
TGATGAAGAGGCAGAGCAATGT	CATCGTCGTCAGCACAAACAAT	897	AATTGACACTCCTAGCTCTGCC	AATGGGTCTTTGATCGTCCTCC	851
AGCATACCAGAAGCAACCAGAT	GCAGTTCCGGATGCTAAAAGAC	774	AAGTGCATGTTTTCCACCAGTG	TGAAATGTGCTCGGGACAAATG	864
GAAGTGACCCTCTTGACAAGCT	TGTTTCGTGATGAGCTGTGTCA	838	TGCAGGTATCAAACTGGTTCGA	ACCACATACCCAGAAGTTTTGGT	868
TGTACCGAACCAAACCAAAATGT	TCCACGAGCAAAACTTTAAGCA	841	ACAGCTCAAGTTCTTCCCACAA	CAAAGCTGGTTCAACGTTGGAT	804
AGAGATTTGAGCGTCGAGACAG	TGCTACAACGAGCACTAACCAT	838	AGAAACGTTGAGAGTGCGAGAT	TCTCAACAATCTGGCTGCTCAT	765
TAGACCCTTTTCTGCAGCAACC	GCTAACAGAAACTCCATCCCCA	782	GGTACAGTTTGTTGGGTTGAGC	GCATTATACTCCCGCGCAAAAT	887
CCAATCATCGGGAAGCCTACTA	CTGCGAGTTTTCCTTCGTCTTC	755	TAAAGCAGAGCACATGAAGGCA	CCTCTTTTGGGTCGCAATTTCT	897
CGTATCCCATTTTCTTTCGACCA	TGACTATGAAATCTTACCGCCTGA	809	CCTGCCGCCATAAATTAATCCG	CTTTTGTTGCCCTCCCATGTTT	778
TGAGTTACCCTGAGCTTTATTCGT	TCTCGTTCACCAAAATCGGACT	871	AAAATGGTAAGTGACGTGTTGGT	CTGGATCCATTTGTGTGCCTTT	893
TGATCTGCTCTTCTTTCTTGCG	ACTGTTCGGTCTGGTAAGTGAC	828	TGGTTTAGCAAGTGGGAGAGAC	TCCACCAACCTGTAACACCAAA	826
CGAGCAGAGAGGTTGGCTAAAA	TCGATTTCTCAACCCACGTGAT	750	TGGGAATCAGATGGAGAGGCTA	GAAGATTTACGAGTGATGTGCGA	896
ATCCTGTGACAAGATCCGGATG	TTACCCCTGCAATGTCAGGTAC	885	TCATGCCTACACTTGTCTTCCC	GAAGCCGTCGGTCTATTGGTAT	793
CAATCCGGTCTTCTTTTGCCTT	CAAGAAGCAAAAGAAGAACAAAGCA	757	AAGGAAGAAGGAGGACGTTGTC	TTTTCGGATCGGATTTGCTTCG	824
TGTAACAGGACCACAATCACCC	GCTTGCGCTTTGGTTGATGATA	824	AAGAGCCATCCGAACATTAGCA	AAAGTTGCCCGTTTGTTACTACA	800
CTATGGTTCCAGTCAAGCCACT	TCACTATGATCCACCGCAAGTC	771	GTGACCAGTTCTTCGTGTACCA	TTCATGTATCCGCTGCTCCATT	797

TGCAAGAACTCCACCAACATTG	AACCATTAAACCACCATTGCCG	797
CCAACTAAAGGAAGAAAGGCAAGT	TTTCATTCAGTTCCACCGTCCA	786
TTACCCTTTTCTTCCCAACGGT	GGATGAGATTCTCTGCCACACA	841
ACCTGATTCCGAAGATGCATCA	CTTACCAAGACTCGCCCAGAAA	871
GCTTCTCCTCCTCCATGAAACA	AATTCATCTGGCCGGATCTGAA	776
GCCTCTGCCTCTCTATTCTTACT	GTGACTTTTGGTTCGGTTTGGT	848
TCTCGCCCATAAGTTTCCATCT	CATCCGGTTCCTCATGTGCATA	862
TCTGGTTCTGCTGTGGTTTGAT	GACGATCTTTTGCACGGTATGG	820
TTGTCCTGCGTATAAACCCGAA	CAGATCTGAAGAACAACAAGGAGAA	780
ATCTTCTTCCTGGGCAATGTGT	TGTATCCGGACGTGTTGATTGT	802
TCCACAGATGACTCGGAACAAG	GTGGTCTCGTCTTCATCAACCT	770
CTCCACGCATCGACTGAAAATG	TGCTGAAAGAGTTGTGAGAAAGC	755
TCAGGTCGTTTTAGGGTGTCTG	CCTTTCAGAGCTCTCCCAAACT	779
GGAGGAGATGAGACAAGAAGCA	TACTACAGCTTCCCCTCCTCAA	765
GTTACTGGCAAGCGGATTCAAG	CCTTTCCCTAACTGCAGAACCA	774
TTACAGGGAACAAGAGCGAACA	TCAGTTCGTAGCAGCCTTTCTT	809
ACATCCCACAAATTAGTCTTTGGTT	CATGGAGCACAAATCGAGAAGC	832
GACAGACTCCAACATCTGACGG	TGGAGGCATCTTTTAGGTGAGAG	770
ATTTGTTGCAGGGTTCCCAAGA	CGGTCTTCTTCTGGTGGTTGTA	799
TGTTTCCACACTTGTCAATGGC	CGTCCTCGTAATTGTTCCCATC	795
CTGATCAAACCTCGCTGACTCT	TAAAAGGAATGGGGCCAATCGT	788
TGATACACACACACCTTGAGCA	AGGAGTAAGTGTCGTTGGTTCA	814
ATTGCCGCGAGTATGAACATTG	AGAGACAAAGGAGGCGGAGATA	881
GATAATTTAACCTCACGCGCCG	GATGCTTTTCACACAGACCACC	798
AACTGCTTTGAGGCTTTGTCTG	AGCGCTTAGGGTTGTTAGAAGT	803
AAGAGAGTAATGTCGTCGGCTC	TTGAGGTACAGGCAATGGTTGT	834
GCTAGTGTTTGCCAAGCTTCAA	TCTAGTCTCGTCTCGTCTCATCT	809
CTTTCACACCGCAAACCCAAAA	TGATGTTGGGAACTTGCTACGT	771
CCTCTGTTGTAGCCACCTTGAT	ACAACTTTGAAATCCGTGGCAG	771
CCGGCCATAGGATGTGTATTGA	GCGGCATAGTCTGAAGCATTAC	776
AAACAAACGCAATGGGGCAATA	CCAGTTCCTCCAAAGCCGTATA	753
ACTCTGGACACTTGCCACAATT	AACACGTAATGAGCTGTGAAAGG	786
TTGCCGGTGATAAACAGTAGCT	TGATGGATTGTTGAATTGGGCAT	768
GTGATGCTTTTGATGTCGGCTT	GGCATTCCATCGTTTTCATCGT	825
GGTTGATATGGACTTGGGAGGG	AGGGTGGAATCGATGATGAAGG	787
TTCTCCTAAACAGTTGGCACCA	CACCACATGATCTTTCCCCGTA	884
GCTAGCTAGTTGTCTTGAAAATGGT	ACGTTTGTGAAATTGACTCGCT	759
GTGATACTAGACGCAAAATTTCCCA	TCCTACAGATTAAACCACACACACA	788
ATCCCTAACACAGCTAGCATGG	ACCAAACATGTTACGCCTGAGA	804
ATGCCCATAAATTTCAGCGCAA	GGCTTCTTCCCCATAGCTTTCT	870
GTAAAGACCAAGCCGACCAGTA	ATATGCGTTGAGTCACCTGGAG	851
AAAAGGAACAGACGAGTGCAGT	TTGAGGAATGTCTTAGGGCCAC	857
GGAGATAAGCCACCACTGTTCA	GAAAATTCAACCCAGAACCGGG	790
GTGACGTCATAACCTTCACCCT	GTTGGGAGATTCTGGTCATCGT	751
GTGATTCTTCCGGCCTCTAGTT	TAGTCCATACCCTTCGACCCTT	806
GATCTCTACGCTGCTCAAGTCA	TTCTTGAGGTATTGGTGGGTCG	803

TTGATGCTTCCTCTCAACGGTC	AAAGGCATTGCACCAGATTGAA	894
AGCACAACAAGTTCACAACCAA	ACGACATCATTTTGCCCAATTCA	782
TGGTGAAGCTTGGTGTATCATCT	TTCATTCACCGCAAGTCTCACT	763
GGAACTCACAACGAAACCCAAG	CGCGTGTTGGTAATGGTGTATC	787
TCCTAACTACTGGGTGGAGTGT	TTCATCCCAACCAACCTACACC	780
TCTCACAACGTTCCTGTAATCACA	ATCCTCCACGTGTCATAGCATC	784
CAGAGTTCTCACACTTACCACCA	TACCAGTCCACGAACTCAACTG	768
AATTTGGCTGAACTTGCTGTCG	ATCCCAACTTCATTCTCCACGG	769
CTCCTCCGGTCAGTTGAAACAT	TCTCTCTACAGCTCTACTTGTCTCT	851
ACAAAATGGAATGGCGGAAAGG	AAGAGAGCAAGGGAAGACACAG	825
TGAATCTTGACCTTCGTGACGT	GATTTGGCTTGATCCCGACATG	833
GGTTATTTGTTGGATGAGGGCC	AGCTATACATGCTTCCTGAACCA	823
CCATGCTCCAAACGACCATAAT	CGGCCTGCTTATCATGGTAGTA	888
AGGAAAAGGCCATGATACCCAA	TGAGATGTGGCCACTCTTTCAA	777
TGTGGACCATAAGCCCAGAAAA	AGAGTGGCTTCATATGATCGATGA	795
ATATGAATCCCAGGCTCGACAC	TAATGAACAATGGTCGCGGAAC	857
AATGCATGTTGGTCCAAATCGC	CAACAACCACCATTTGACCATCA	750
ACCACAGTGTCGAGCAATCTAG	CCGTCGCTCTCAGATTCTTCTT	899
ACCAACATCTATCCATCACCCTAA	CCACCTCCGGATTTATTGGGAA	766
TCTGATTCCAAGGCCAGTCATC	ACTTCATCGCCCAATGTAAGGT	776
CTTGATGAGTGCGGTAAGAGGT	CGTCAATCCGAAAGAAGCCAAA	763
TGCATTTACTGATTTGTGCGCA	GGGTTCGGTTGGTTTGAATCTG	841
TCAGTTGACATTCCACGAGTTTG	GTTGTTGTGGTATTGCGCAGTA	802
GCTGTGGTGAAAGTGGAGAATG	TTCACTTGCAACACGAACAGTC	767
TGATGAGCTCGAAGGTTGTTGA	ACCACGATTTCTAGACTGCCAA	851
CACTGATGGCCATTGATGCAAA	CTTGACTTGAGAACAGCCAACA	772
CCAATGCATACTTCCTTTGACACA	ACCAATCTCCTTTGCTCAATCCA	757
AACCCCAGCGAACCTTTGAATA	CAAGAACGCGTGGTCTTTCTTT	810
GCCGAAAATCTCACATACACGG	TGCCATCACTTTCTCCTGATCG	854
GTAAGAACATGCTCGCGGAATT	CACTAGGAGGAGAAGGGTGAGA	802
CTTCCCTTCTTTGCACAACACA	CCGAAAATGCCCAATCCCAATT	810
TACTGGCCGTAAGTTTCGGTC	AAGCTCAAATATCAAACCAGTCCAT	790
TGTGGTGAAGGTGATTGGTTCA	GGGATTCGAGTTATACCGACGT	774
TGCATCAAATCTTGTGACACTCTT	GTTCGACAATCACCAGCCAAAA	831
TCGACAAATGATTACCTGGCCA	CAGTTTCCCAGCAAACCACAAA	752
GTCAAACCCTCCTCCTCATCTG	TTCTATTTCTGGCAAAAGGAGGATG	789
ATTGTGTAGTTGGCCTTCGAGT	GCTTGAATCTTGACAGCTGCAA	879
GTTGTTTACCAGCTTCGCCATT	AGCATGAAGTTCCAACCTTCCA	760
ACTGACCGTGCCTTAATGAGAG	AGACAAACTCCTTCCCGCTTTA	783
CGGGACTCTAAGGCAATTACCA	TGATGGCAGCAAAAGTTTTCAAC	751
GCGCAGGTCACATCAAGATTAC	TCGTTAAACAGTCACACCAGCT	806
AGAGGTTGGGCGATAATCTTTCA	ACACAAGTTAACCAAATGAGCCA	805
GGTGAAGCCCTAGTGAGTGATC	CTTTAGCAGTAGTCTCCTCGGC	766
ATCTTCATCAATCTCGCACCGA	AGCCAAACATAGAGGAACAGAACA	894
GTTTCGTTTCCACTTTCCACCG	AAATTCGATTCTGTAGGCGGGA	774
ACCCCAATGCACAGATTCACAT	TCCTCTGTGTCTGATGATTCCG	770

ATGCATCGGTGTACAAGAACCA	CTGACGGTGAGCAAAACATCTG	846
AAACGTTCCACAACCAAAAGCA	CCTTCATTCCAGTCTCGAGCTT	751
TGGCGTCATTTCAGTTCCCATA	TGTGTCTTTGCTCTGTTCTGGT	842
TCTCCAACTTGTTGCGGTTTTC	GCCAGATGAATAGATCGGAGCA	760
ACGAATAGCAACAAGACCAAGTC	GCTGAGATTCTTGAGCTGACCT	820
TCGTGTGTGAAATCCGAATTGC	TGGAGACTCGAGTTCAACATCG	763
TCTGGACTGTGAGAGAATTCGC	TTTTCGGATGTGAGAGATGGGT	753
GAACGAGTGAAGCCTTGACAAC	TCCCATGGTTTTAGGTGAGATTTCA	782
CAGAGATTAGTGCCATCCCATGA	TGGGTTTGAAGTTCGAGGTTCA	771
TTCCCTAGAAATGTGCGTCCTT	ATGGAATGGAGGCTGTAGTTCC	769
CCTCACGGCACTGATTGGTTAT	AATAATCTGTTGCATGGCTGGC	787
TGTCGCATATCCAATCCATCACA	CTTTGACTGTATGACTTTGCTGGT	796
ATCGAAACTTTAAAGGACCAACACT	CCCTCCGACACCATATTGCTTA	812
ATGCAAAACAGTCTTCCTCCGA	AGCTTCCATTTCCCTTCCTCTG	763
TGCATCACTCTCTCCTTCTTCAA	CAACGGCTCTTTCCCTTTTCAG	761
CTAGATGGTCGAGTGGCTCTTG	ACGCAAAACTCTCCAACTTCTC	769
AGAGAGAGTGAGTGATGTCGAGA	TGGGAGATGGGAAGAGAAGAGA	751
CGGTCATGGTACTCGATTCGTA	CTCGGACAGCTTACTCAGTCTC	751
AGTGACCAAGGTTTTCTGAGCA	TGGATCCGAGATTTGGATGTCC	764
CGAATACCCGATCTGAACCGAT	TAGAGGCAGCTTGGGTTCTAAC	768
ACCATTCACTCGAGATGATGCT	ACACGGGTGAATCAACAGACTA	856
AGAGAAGCTATTTGGTGTGGCA	AATCTCTTGCCTTGTTCACGTC	883
AGTGTACTCAAGGACGAAGCTG	GGCGAGAAGGAGAAGAGACAAA	765
TGCAGGTCTGAAGTTGATCACA	AAAACCCGTAGAATCATGGCCT	751
TGCCCTCATTGTTGATCTAAGGT	TGGAAGTATTTGGGCTCGTAGG	814
ACCAGGGTTGTCAATCATTGGA	TCCAATTCTCTGTCAAGCACGA	874
TGAAACGGTAACAAAGTCGTGC	GGGAGAGTTGGAGTCTTGAAGG	770
ACGAAAGAGAGACCAAATCGCA	AGTTATGGCTTCAGATGTGGCA	759
TGTGGGATGTTGTTGTTGTTGG	TAAACCAAGGCATCACGGAGAA	770

CGTCTCTTTCGAACATTGCTCC	TTCTTGTGATCGAAGTCCCCAG	757
GGATCCAGTGGTTGAGGCAATA	ACCAAATAGAAGAGCCAAGCCA	761
CACGTCTACTCTCACCACTTCC	ACGTTCTGTTTCGGGATGTGTA	883
GATAGGCTCTGCTTCCACATCA	TCAATGGACACACATATGACCATCT	861
GCTTCTGCGTCATCTTAAACCG	ATCCTATAAAGCATCTCCGCCG	751
ACTCCAGAACGATTTGTACCCC	AGTTTGGTAAAAGCTCGGTCCT	859
AGAGAGCAATGGTTGTACACGA	AAGCTTGGGTGTGGAATAGAGT	774
TGGGCCCAATTTGAAATGTTCC	GTTGCAGGATGAGGAGACAGAT	875
AGGAGGGTGAAACATTTGCCTA	TGGCAAAACTGAGCTAAGGGAA	771
GGACACTGAAGGGGCTTGAATA	TCGCTTCCTTCTTCCATCTCTG	839
ACCAAATCACTGAAACTGAAAATGC	CTCAAACGCAAAAGCTGGAGAT	898
CCAGCAATACCAGCAGCAGTAT	CCTGGTGTTGTTGAGAGCCTTA	843
TATGCAAAGCTGACTGATGGGT	TCGGAACATAGACCGTAAACTACA	879
CTCTCTGCACATCCACTCTAGC	GCGAACTCTCTATCTTCTCCGG	788
CTCGTTTCCTGCTTCTCCTTCT	GGTAACTGTGAGTGCTGCTTTG	785
CTGGTGTCGGATTGTTATGCAC	TTGTGATGTTTGGGGTCCGTAT	769
TGATCTGCATTCTCAACACACCT	TGACTGGATGACATGTTTAGCGA	849
TCTGTAAATGGGGTTTGACTCGA	ACGCTAAAACTTACGATTCAGACAC	754
CGGATGAGTTGCCACAAGAATT	ACCATGAAAGCTGCGTACCTTA	780
GGCTTTGTGTTTTCCATGGTGA	GCGGCATTAACAGCTTCCTATG	776
TTTTGTTGACCCCGCCAATTAT	ATGGCCGATCATCATCATCATCA	750
GGGCAGGTTCGGATCAGATATC	CATGTGCTCGGTTACTCTCACT	876
ATCACTTTGAACTCGACGGTCA	TGTGGAAACTCGTGACTTGGAA	826
GCTTCCTTCTGTGGTGTTGTTG	CATCGTAGTAGCCAGCAGAGAG	759
TATTGACTCGGTGCATCTCTCG	CCACTCTAACTCAGCAGCTTCA	763
TTTCGGCTAACCTTGCTACCTT	AGTATAGTAAGGATTTGTCCCGGAC	761
TGGGGTGGTTTTGTTCTTGAGA	TGGCAGAGCGTACCATATGATC	796
CAATTCGTGGATGATGAGATTGGT	GCATCTTAGCTCTTTTGTTGCCT	796
GCTCTTAAACGAAGCGTTACG	CAACTCTATATTGGATTGGATTCC	410

Supplementary Table 3. Comparison of gene statistics of *A. thaliana*, *A. lyrata*, *C. rubella*, *C. hirsuta*, *B.rapa, T.parvula, E. salsugineum,* and *A. arabicum.*

	A. thaliana	***A. lyrata***	***C. rubella***	***C. hirsuta***	***B. rapa***	***T. parvula***	***E. salsugineum***	***A. arabicum***
#gene loci	27416	32670	26521	29458	41020	27132	29284	23167
#transcripts	35386	32670	28447	37997	41020	27132	29284	23167
mean CDS size [bp]	1230.6	1084.1	1253.6	1148.7	1173.2	1185.3	1233.8	1345.6
median CDS size [bp]	1050	888	1080	951	981	999	1053	1089
mean intron size [bp]	156.9	202.9	169.5	163,8	210.4	189.8	173.7	238.4
median intron size [bp]	99	100	102	96	96	106	104	103
mean exon size [bp]	220.9	223.1	240	223	232.9	226.8	225.8	241
median exon size [bp]	127	132	135	129	137	133	130	135
#exons/transcript, mean	5.6	4.9	5.2	5.2	5.0	5.2	5.5	5.6
#exons/transcript, median	4	3	4	4	3	4	4	4

# tandem cluster	1727	2010	1732	1708	2189	1169	1553	843
#tandem loci	4792	5488	5041	5270	5474	2907	4265	2151

Supplementary Table 4. Enriched InterPro terms in the unique gene families of *C. hirsuta*.

InterPro ID	P-Value	InterPro Name
IPR012337	0	Ribonuclease H-like domain
IPR003653	0	Ulp1 protease family, C-terminal catalytic domain
IPR015410	0	Domain of unknown function DUF1985
IPR021704	0	Protein of unknown function DUF3287
IPR001584	0	Integrase, catalytic core
IPR010285	0	DNA helicase Pif1-like
IPR021109	0	Aspartic peptidase domain
IPR010666	0	Zinc finger, GRF-type
IPR021139	0	NYN domain, limkain-b1-type
IPR003871	0	Domain of unknown function DUF223
IPR005162	0.000001	Retrotransposon gag domain
IPR002156	0.000009	Ribonuclease H domain
IPR010851	0.000084	S locus-related glycoprotein 1 binding pollen coat protein
IPR006941	0.000202	Ribonuclease CAF1
IPR016027	0.003149	NA
IPR012436	0.003149	Protein of unknown function DUF1633
IPR013242	0.008667	Retroviral aspartyl protease
IPR003656	0.009079	Zinc finger, BED-type
IPR016897	0.010525	S-phase kinase-associated protein 1
IPR016073	0.012071	SKP1 component, POZ domain
IPR022618	0.01396	Defensin-like protein
IPR001232	0.014168	S-phase kinase-associated protein 1-like
IPR016072	0.014168	SKP1 component, dimerisation
IPR000668	0.014168	Peptidase C1A, papain C-terminal
IPR008906	0.016396	HAT, C-terminal dimerisation domain
IPR000403	0.020425	Phosphatidylinositol 3-/4-kinase, catalytic domain
IPR008801	0.022488	Rapid ALkalinization Factor
IPR001878	0.02316	Zinc finger, CCHC-type

Supplementary Table 5. Enriched InterPro terms in the expanded gene families of *C. hirsuta*.

InterPro ID	P-Value	InterPro NAME
IPR001611	0	Leucine-rich repeat
IPR000719	0	Protein kinase domain
IPR008271	0	Serine/threonine-protein kinase, active site
IPR011009	0	Protein kinase-like domain
IPR017441	0	Protein kinase, ATP binding site
IPR017442	0	NA
IPR001245	0	Serine-threonine/tyrosine-protein kinase catalytic domain
IPR000767	0	NA

IPR002182	0	NB-ARC
IPR000858	0	S-locus glycoprotein domain
IPR001480	0	Bulb-type lectin domain
IPR003609	0	PAN/Apple domain
IPR013227	0	NA
IPR013101	0	Leucine-rich repeat 2
IPR013596	0	NA
IPR022364	0	NA
IPR006912	0	Harbinger transposase-derived protein
IPR001810	0	F-box domain
IPR006527	0	F-box associated domain, type 1
IPR017451	0	F-box associated interaction domain
IPR005174	0	Domain unknown function DUF295
IPR022052	0	Histone-binding protein RBBP4, N-terminal
IPR013187	0	F-box associated domain, type 3
IPR011713	0	Leucine-rich repeat 3
IPR010285	0	DNA helicase Pif1-like
IPR013320	0	Concanavalin A-like lectin/glucanase domain
IPR011043	0	Galactose oxidase/kelch, beta-propeller
IPR000157	0	Toll/interleukin-1 receptor homology (TIR) domain
IPR021820	0	S-locus receptor kinase, C-terminal
IPR007053	0	LRAT-like domain
IPR002902	0	Gnk2-homologous domain
IPR001525	0	C-5 cytosine methyltransferase
IPR005314	0	Peptidase C50, separase
IPR001220	0	Legume lectin domain
IPR008985	0	NA
IPR003480	0	Transferase
IPR007259	0.000004	Gamma-tubulin complex component protein
IPR023213	0.000004	Chloramphenicol acetyltransferase-like domain
IPR002885	0.000006	Pentatricopeptide repeat
IPR004129	0.000006	Glycerophosphoryl diester phosphodiesterase
IPR008591	0.000016	GINS complex subunit Sld5
IPR004993	0.000105	GH3 family
IPR022702	0.000276	DNA (cytosine-5)-methyltransferase 1, replication foci domain
IPR017946	0.000276	PLC-like phosphodiesterase, TIM beta/alpha-barrel domain
IPR000742	0.000884	EGF-like domain
IPR005379	0.00163	Uncharacterised domain XH
IPR001360	0.001816	Glycoside hydrolase family 1
IPR018117	0.003062	DNA methylase, C-5 cytosine-specific, active site
IPR008906	0.00645	HAT, C-terminal dimerisation domain
IPR013210	0.007293	Leucine-rich repeat-containing N-terminal, plant-type
IPR001320	0.023317	Ionotropic glutamate receptor
IPR001638	0.023317	Solute-binding protein family 3/N-terminal domain of MltF
IPR001828	0.023317	Receptor, ligand binding region

IPR008808	0.047089	Powdery mildew resistance protein, RPW8 domain

Supplementary Table 6. Enriched InterPro terms in the unique or expanded families of *C. hirsuta*.

InterPro ID	P-Value	InterPro Name
IPR012337	0	Ribonuclease H-like domain
IPR001611	0	Leucine-rich repeat
IPR000719	0	Protein kinase domain
IPR008271	0	Serine/threonine-protein kinase, active site
IPR011009	0	Protein kinase-like domain
IPR017441	0	Protein kinase, ATP binding site
IPR001245	0	Serine-threonine/tyrosine-protein kinase catalytic domain
IPR000767	0	NA
IPR002182	0	NB-ARC
IPR000858	0	S-locus glycoprotein domain
IPR001480	0	Bulb-type lectin domain
IPR003609	0	PAN/Apple domain
IPR013227	0	NA
IPR013101	0	Leucine-rich repeat 2
IPR013596	0	NA
IPR022364	0	NA
IPR006912	0	Harbinger transposase-derived protein
IPR001810	0	F-box domain
IPR006527	0	F-box associated domain, type 1
IPR017451	0	F-box associated interaction domain
IPR005174	0	Domain unknown function DUF295
IPR022052	0	Histone-binding protein RBBP4, N-terminal
IPR013187	0	F-box associated domain, type 3
IPR011713	0	Leucine-rich repeat 3
IPR003653	0	Ulp1 protease family, C-terminal catalytic domain
IPR015410	0	Domain of unknown function DUF1985
IPR021704	0	Protein of unknown function DUF3287
IPR001584	0	Integrase, catalytic core
IPR010285	0	DNA helicase Pif1-like
IPR013320	0	Concanavalin A-like lectin/glucanase domain
IPR011043	0	Galactose oxidase/kelch, beta-propeller
IPR000157	0	Toll/interleukin-1 receptor homology (TIR) domain
IPR021820	0	S-locus receptor kinase, C-terminal
IPR007053	0	LRAT-like domain
IPR002902	0	Gnk2-homologous domain
IPR001525	0	C-5 cytosine methyltransferase
IPR005314	0	Peptidase C50, separase
IPR010666	0	Zinc finger, GRF-type
IPR001220	0	Legume lectin domain
IPR021139	0.000003	NYN domain, limkain-b1-type
IPR008985	0.000004	NA

IPR003480	0.000005	Transferase
IPR023213	0.000012	Chloramphenicol acetyltransferase-like domain
IPR007259	0.000014	Gamma-tubulin complex component protein
IPR017442	0.000016	NA
IPR004129	0.000027	Glycerophosphoryl diester phosphodiesterase
IPR005379	0.00003	Uncharacterised domain XH
IPR008591	0.000047	GINS complex subunit Sld5
IPR008906	0.000051	HAT, C-terminal dimerisation domain
IPR021109	0.000227	Aspartic peptidase domain
IPR002885	0.000447	Pentatricopeptide repeat
IPR004993	0.000559	GH3 family
IPR003871	0.00063	Domain of unknown function DUF223
IPR022702	0.000776	DNA (cytosine-5)-methyltransferase 1, replication foci domain
IPR017946	0.001209	PLC-like phosphodiesterase, TIM beta/alpha-barrel domain
IPR005162	0.002824	Retrotransposon gag domain
IPR000742	0.002897	EGF-like domain
IPR018117	0.007741	DNA methylase, C-5 cytosine-specific, active site
IPR001360	0.009262	Glycoside hydrolase family 1
IPR002156	0.016529	Ribonuclease H domain

Supplementary Table 7. Enriched InterPro terms in up-regulated *C. hirsuta* meristem genes.

Interpro ID	p.value	description
IPR018422	0,000848823	Cation/H+ exchanger, CPA1 family
IPR013775	0,007145166	Alpha-amylase, plant
IPR006046	0,006156608	Alpha amylase
IPR024593	0,0071466	Domain of unknown function DUF3444
IPR007942	0,003007124	Phospholipase-like
IPR007125	0,009539114	Histone core
IPR014811	0,0071466	Domain of unknown function DUF1785
IPR003165	0,008385627	Stem cell self-renewal protein Piwi
IPR001019	0,003007124	Guanine nucleotide binding protein (G-protein), alpha subunit
IPR011025	0,003007124	G protein alpha subunit, helical insertion
IPR003140	0,0071466	Phospholipase/carboxylesterase/thioesterase
IPR001471	0,003020799	AP2/ERF domain
IPR016177	0,004466336	DNA-binding domain
IPR013899	0,003961402	Domain of unknown function DUF1771
IPR025836	0,009549559	Zinc knuckle CX2CX4HX4C
IPR007290	0,009056722	Arv1 protein
IPR022648	0,007145166	Proliferating cell nuclear antigen, PCNA, N-terminal
IPR000730	0,009568302	Proliferating cell nuclear antigen, PCNA
IPR022659	0,006329155	Proliferating cell nuclear antigen, PCNA, conserved site
IPR020708	0,006329155	DNA-directed RNA polymerase, 14-18kDa subunit, conserved site
IPR012293	0,006329155	RNA polymerase subunit, RPB6/omega
IPR006110	0,006329155	RNA polymerase, subunit omega/K/RPB6
IPR006111	0,006329155	Archaeal RpoK/eukaryotic RPB6 RNA polymerase subunit
IPR026851	4,77631E-18	Dna2
IPR009686	0,004092719	Senescence/spartin-associated
IPR011893	0,001055307	Selenoprotein, Rdx type
IPR008395	0,009539114	Agenet-like domain
IPR004518	0,003961402	NTP pyrophosphohydrolase MazG, putative catalytic core

IPR015216	0,000394526	SANT associated
IPR024326	8,30151E-05	Ribosomal RNA-processing protein 7
IPR022765	9,22023E-08	Dna2/Cas4, domain of unknown function DUF83
IPR014808	5,10249E-09	DNA replication factor Dna2, N-terminal
IPR002755	0,006156608	DNA primase, small subunit
IPR014052	0,007145166	DNA primase, small subunit, eukaryotic/archaeal

Supplementary Table 8. DEseq results for enriched transcription factors up-regulated *C. hirsuta* meristem genes.

gene id	A.thaliana	C.hirsuta	foldChange	Gene Name	Description
AT2G23340	336.63	770.89	2.29	DEAR3	ethylene-responsive transcription factor ERF008
AT4G16610	41.56	151.80	3.65	AT4G16610	C2H2-like zinc finger protein
AT1G26260	62.80	162.01	2.58	CIB5	transcription factor bHLH76
AT5G04840	143.62	461.98	3.22	AT5G04840	bZIP protein
AT5G66350	42.91	131.64	3.07	SHI	Lateral root primordium-related protein
AT5G65510	40.84	1166.91	28.57	AIL7	AINTEGUMENTA-like 7 protein
AT5G57390	191.57	756.34	3.95	AIL5	AP2-like ethylene-responsive transcription factor AIL5
AT5G56270	235.78	515.99	2.19	ATWRKY2	putative WRKY transcription factor 2
AT5G51990	0.00	5.45	Inf	CBF4	dehydration-responsive element-binding protein 1D
AT5G46880	651.22	1337.28	2.05	HB-7	homeobox-leucine zipper protein HDG5
AT5G03720	23.89	122.14	5.11	AT-HSFA3	heat shock transcription factor A3
AT3G61250	237.80	1017.27	4.28	AtMYB17	myb domain protein 17
AT3G50870	63.75	220.26	3.46	MNP	GATA transcription factor 18
AT4G37750	2347.05	6714.33	2.86	ANT	AP2-like ethylene-responsive transcription factor ANT
AT4G34000	179.59	407.40	2.27	ABF3	abscisic acid-insensitive 5-like protein 6
AT4G32890	165.42	454.55	2.75	GATA9	GATA transcription factor 9
AT4G31630	0.00	1.28	Inf	AT4G31630	putative B3 domain-containing protein REM4
AT4G28140	7.38	21.88	2.96	AT4G28140	ethylene-responsive transcription factor ERF054
AT4G21550	68.38	168.78	2.47	VAL3	B3 domain-containing transcription factor VAL3
AT4G08150	1.85	24.76	13.36	KNAT1	homeobox protein knotted-1-like 1
AT4G02670	22.28	120.15	5.39	AtIDD12	indeterminate-domain 12 protein
AT4G02590	704.88	1595.32	2.26	UNE12	transcription factor UNE12
AT1G75240	1879.77	4697.19	2.50	HB33	homeobox protein 33
AT2G34140	10.46	56.81	5.43	AT2G34140	Dof zinc finger protein DOF2.3
AT3G15170	1.33	18.09	13.63	CUC1	protein CUP-SHAPED COTYLEDON 1
AT3G19184	2.38	229.11	96.16	AT3G19184	AP2/B3 domain-containing transcription factor
AT3G25730	22.83	113.55	4.97	EDF3	AP2/ERF and B3 domain-containing transcription factor ARF14
AT3G21810	154.11	474.81	3.08	AT3G21810	zinc finger CCCH domain-containing protein 40
AT3G25990	8.98	53.62	5.97	AT3G25990	DNA-binding protein GT-1-related protein
AT3G14230	1503.61	8964.60	5.96	RAP2.2	ethylene-responsive transcription factor RAP2-2
AT3G10500	95.67	365.95	3.83	anac053	NAC domain containing protein 53
AT1G68120	129.05	411.31	3.19	BPC3	basic pentacysteine 3
AT1G80580	0.00	1.57	Inf	AT1G80580	ethylene-responsive transcription factor ERF084
AT1G62360	1.75	12.14	6.94	BUM	homeobox protein SHOOT MERISTEMLESS
AT1G04880	10.18	79.14	7.77	AT1G04880	high mobility group-box and ARID domain-binding domain-containing protein
AT1G64620	39.45	138.21	3.50	AT1G64620	Dof zinc finger protein DOF1.8
AT1G54160	11.37	35.49	3.12	NFYA5	nuclear transcription factor Y subunit A-5
AT1G36060	4.10	53.47	13.04	AT1G36060	ethylene-responsive transcription factor ERF055
AT2G17770	11.95	196.41	16.44	ATBZIP27	basic region/leucine zipper motif 27-containing protein
AT2G22760	0.00	1.70	Inf	AT2G22760	transcription factor bHLH19
AT2G22770	154.40	553.90	3.59	NAI1	transcription factor NAI1
AT2G34710	760.16	1684.58	2.22	PHB	homeobox-leucine zipper protein ATHB-14

AT2G32930	94.16	257.68	2.74	ZFN2	zinc finger CCCH domain-containing protein 26
AT2G42150	29.73	250.77	8.43	AT2G42150	DNA-binding bromodomain-containing protein

Supplementary Table 9. DEseq results for differentially expressed PME(I) genes in *C. hirsuta*.

chi.gene	chi.stage9	chi.stage16	chi.foldChange	chi.padj	ath.gene	ath.stage9	ath.stage16	ath.foldChange	ath.padj
CARHR143060	4.90	143.76	29.32	4.13E-04	AT2G47670	1.60	5.29	3.31	0.90
CARHR085300	229.62	1730.60	7.54	2.49E-02	AT3G10720	280.87	255.63	0.91	1.00
CARHR118350	13.51	265.95	19.68	7.01E-04	AT2G26440	640.40	702.79	1.10	1.00
CARHR173850	682.48	48.41	0.07	1.98E-03	AT5G47500	1621.01	1831.29	1.13	1.00
CARHR043880	0.00	9058.78	Inf	1.55E-18	AT4G00872	0.00	0.59	Inf	1.00
CARHR214060	0.00	34.50	Inf	1.45E-03	AT5G38610	4.01	9.38	2.34	0.90
CARHR089480	700.32	6075.53	8.68	1.22E-02	AT3G14310	950.92	1070.50	1.13	1.00
CARHR276140	115.84	806.03	6.96	3.49E-02	AT5G62360	258.72	446.94	1.73	0.80
CARHR004800	0.00	36.02	Inf	1.37E-03	AT1G05310	0.40	0.58	1.46	1.00
CARHR156040	5.70	66.06	11.58	2.63E-02	AT3G47400	23.32	43.98	1.89	0.82
CARHR045850	1.33	19915.54	15022.14	1.05E-18					
CARHR044320	0.00	22229.74	Inf	1.10E-20					
CARHR089500	0.00	197.29	Inf	2.83E-08					
CARHR213450	0.00	42.74	Inf	3.36E-04					
CARHR213460	0.00	17.56	Inf	2.74E-02					
CARHR265360	0.00	24.21	Inf	6.81E-03					
CARHR265370	0.00	16.05	Inf	4.01E-02					

Supplementary Table 10. Comparison of the assembly performance by SOAPdenovo with or without using the 10kbp library.

	SoapDenovo without 10K library	SoapDenovo with 10K library
Number of scaffolds	103,158	103,592
Total length	209,779,505	212,726,400
max size	3,192,244	1,481,499
min size	100	100
average size	2,033	2,053
N50	509,531	266,184
N90	34,762	41,053

Supplementary Table 11. The list of sequences in NCBI Genbank used for the phylogenetic analysis of *SRK* gene

Species	Subspecies	Accession	Short Name	Description
Arabidopsis arenosa		JX464613	AareSRK06	Arabidopsis arenosa S-receptor kinase SRK06 (SRK) gene, partial cds.
Arabidopsis arenosa		JX464617	AareSRK14	Arabidopsis arenosa S-receptor kinase SRK14 (SRK) gene, partial cds.
Arabidopsis arenosa		JX464633	AareSRK74	Arabidopsis arenosa S-receptor kinase SRK74 (SRK) gene, partial cds.
Arabidopsis arenosa		JX464634	AareSRK75	Arabidopsis arenosa S-receptor kinase SRK75 (SRK) gene, partial cds.
Arabidopsis		DQ520278	AhalSRK04	Arabidopsis halleri S receptor kinase SRK04 gene, exons 1

halleri				through 7 and partial cds.
Arabidopsis halleri		DQ520279	AhalSRK10	Arabidopsis halleri S receptor kinase SRK10 gene, exons 1 through 6 and partial cds.
Arabidopsis halleri		EU075125	AhalSRK02	Arabidopsis halleri S-receptor kinase (SRK) gene, SRK-AhSRK02 allele, exon 1 and partial cds.
Arabidopsis halleri		EU075126	AhalSRK03	Arabidopsis halleri S-receptor kinase (SRK) gene, SRK-AhSRK03 allele, exons 1 through 5 and partial cds.
Arabidopsis halleri		EU075127	AhalSRK05	Arabidopsis halleri S-receptor kinase (SRK) gene, SRK-AhSRK05 allele, exon 1 and partial cds.
Arabidopsis halleri		EU075128	AhalSRK06	Arabidopsis halleri S-receptor kinase (SRK) gene, SRK-AhSRK06 allele, exons 1 through 4 and partial cds.
Arabidopsis halleri		EU075129	AhalSRK07	Arabidopsis halleri S-receptor kinase (SRK) gene, SRK-AhSRK07 allele, exons 2 through 4 and partial cds.
Arabidopsis halleri		EU075130	AhalSRK08	Arabidopsis halleri S-receptor kinase (SRK) gene, SRK-AhSRK08 allele, exon 1 and partial cds.
Arabidopsis halleri		EU075131	AhalSRK09	Arabidopsis halleri S-receptor kinase (SRK) gene, SRK-AhSRK09 allele, exon 1 and partial cds.
Arabidopsis halleri		EU075132	AhalSRK11	Arabidopsis halleri S-receptor kinase (SRK) gene, SRK-AhSRK11 allele, exon 1 and partial cds.
Arabidopsis halleri		EU075133	AhalSRK12	Arabidopsis halleri S-receptor kinase (SRK) gene, SRK-AhSRK12 allele, exon 1 and partial cds.
Arabidopsis halleri		EU075134	AhalSRK13	Arabidopsis halleri S-receptor kinase (SRK) gene, SRK-AhSRK13 allele, exon 1 and partial cds.
Arabidopsis halleri		EU075135	AhalSRK14	Arabidopsis halleri S-receptor kinase (SRK) gene, SRK-AhSRK14 allele, exon 1 and partial cds.
Arabidopsis halleri		EU075136	AhalSRK15	Arabidopsis halleri S-receptor kinase (SRK) gene, SRK-AhSRK15 allele, exon 1 and partial cds.
Arabidopsis halleri		EU075137	AhalSRK16	Arabidopsis halleri S-receptor kinase (SRK) gene, SRK-AhSRK16 allele, exon 1 and partial cds.
Arabidopsis halleri		EU075138	AhalSRK17	Arabidopsis halleri S-receptor kinase (SRK) gene, SRK-AhSRK17 allele, exon 1 and partial cds.
Arabidopsis halleri		EU075139	AhalSRK18	Arabidopsis halleri S-receptor kinase (SRK) gene, SRK-AhSRK18 allele, exons 2 through 4 and partial cds.
Arabidopsis halleri		EU075140	AhalSRK19	Arabidopsis halleri S-receptor kinase (SRK) gene, SRK-AhSRK19 allele, exon 1 and partial cds.
Arabidopsis halleri		EU075141	AhalSRK20	Arabidopsis halleri S-receptor kinase (SRK) gene, SRK-AhSRK20 allele, exon 1 and partial cds.
Arabidopsis halleri		EU075142	AhalSRK21	Arabidopsis halleri S-receptor kinase (SRK) gene, SRK-AhSRK21 allele, exon 1 and partial cds.
Arabidopsis halleri		EU075143	AhalSRK22	Arabidopsis halleri S-receptor kinase (SRK) gene, SRK-AhSRK22 allele, exon 1 and partial cds.
Arabidopsis halleri		EU273968	AhalARK3-B	Arabidopsis halleri isolate AL13.10 ARK3 (ARK3) gene, ARK3-B allele, partial cds.
Arabidopsis halleri		EU273976	AhalARK3-A	Arabidopsis halleri isolate AU2 ARK3 (ARK3) gene, ARK3-A allele, partial cds.
Arabidopsis halleri		EU274005	AhalARK3-C	Arabidopsis halleri isolate TC8.2 ARK3 (ARK3) gene, ARK3-C allele, partial cds.
Arabidopsis halleri		EU878008	AhalSRK23	Arabidopsis halleri isolate AhSRK23 S receptor kinase (SRK) gene, partial cds.
Arabidopsis halleri		EU878009	AhalSRK24	Arabidopsis halleri isolate AhSRK24 S receptor kinase (SRK) gene, partial cds.
Arabidopsis halleri		EU878010	AhalSRK25	Arabidopsis halleri isolate AhSRK25 S receptor kinase (SRK) gene, partial cds.
Arabidopsis halleri		EU878011	AhalSRK26	Arabidopsis halleri isolate AhSRK26 S receptor kinase (SRK) gene, partial cds.
Arabidopsis halleri		EU878012	AhalSRK27	Arabidopsis halleri isolate AhSRK27 S receptor kinase (SRK) gene, partial cds.
Arabidopsis halleri		EU878014	AhalSRK29	Arabidopsis halleri isolate AhSRK29 S receptor kinase (SRK) gene, partial cds.
Arabidopsis halleri		EU878015	AhalSRK30	Arabidopsis halleri isolate AhSRK30 S receptor kinase (SRK) gene, partial cds.
Arabidopsis halleri		GQ915343	AhalSRK28b	Arabidopsis halleri haplotype AhSRK28b S-receptor kinase (SRK) gene, partial cds.
Arabidopsis halleri		GQ915352	AhalSRK01b	Arabidopsis halleri haplotype AhSRK01b S-receptor kinase (SRK) gene, partial cds.

Arabidopsis halleri	gemmifera	JX114759	AhalgemSRK62-A	Arabidopsis halleri subsp. gemmifera isolate 62 haplogroup A SRK (SRK) gene, complete cds.
Arabidopsis halleri	gemmifera	JX114763	AhalgemSRK61-B	Arabidopsis halleri subsp. gemmifera isolate 61 haplogroup B SRK (SRK) gene, partial cds.
Arabidopsis halleri	gemmifera	JX114765	AhalgemSRK63-C	Arabidopsis halleri subsp. gemmifera isolate 63 haplogroup C SRK (SRK) gene, complete cds.
Arabidopsis kamchatica	kawasakiana	JX114756	AkamkawSRK51-A	Arabidopsis kamchatica subsp. kawasakiana isolate 51 haplogroup A SRK pseudogene, complete sequence.
Arabidopsis kamchatica	kamchatica	JX114757	AkamkamSRK55-A	Arabidopsis kamchatica subsp. kamchatica isolate 55 haplogroup A SRK (SRK) gene, complete cds.
Arabidopsis kamchatica	kamchatica	JX114758	AkamkamSRK50-A	Arabidopsis kamchatica subsp. kamchatica isolate 50 haplogroup A SRK (SRK) gene, complete cds.
Arabidopsis kamchatica	kamchatica	JX114760	AkamkamSRK48-B	Arabidopsis kamchatica subsp. kamchatica isolate 48 haplogroup B SRK (SRK) gene, partial cds.
Arabidopsis kamchatica	kamchatica	JX114761	AkamkamSRK49-B	Arabidopsis kamchatica subsp. kamchatica isolate 49 haplogroup B SRK (SRK) gene, partial cds.
Arabidopsis kamchatica	kamchatica	JX114762	AkamkamSRK41-B	Arabidopsis kamchatica subsp. kamchatica isolate 41 haplogroup B SRK (SRK) gene, partial cds.
Arabidopsis kamchatica	kamchatica	JX114764	AkamkamSRK58-C	Arabidopsis kamchatica subsp. kamchatica isolate 58 haplogroup C SRK pseudogene, complete sequence.
Arabidopsis kamchatica	kamchatica	JX114766	AkamkamSRK50-D	Arabidopsis kamchatica subsp. kamchatica isolate 50 haplogroup D SRK (SRK) gene, complete cds.
Arabidopsis kamchatica	kawasakiana	JX114767	AkamkawSRK51-D	Arabidopsis kamchatica subsp. kawasakiana isolate 51 haplogroup D SRK (SRK) gene, complete cds.
Arabidopsis kamchatica	kamchatica	JX114768	AkamkamSRK52-D	Arabidopsis kamchatica subsp. kamchatica isolate 52 haplogroup D SRK (SRK) gene, complete cds.
Arabidopsis kamchatica	kamchatica	JX114769	AkamkamSRK55-D	Arabidopsis kamchatica subsp. kamchatica isolate 55 haplogroup D SRK (SRK) gene, complete cds.
Arabidopsis kamchatica	kamchatica	JX114770	AkamkamSRK41-D	Arabidopsis kamchatica subsp. kamchatica isolate 41 haplogroup D SRK (SRK) gene, complete cds.
Arabidopsis kamchatica	kamchatica	JX114771	AkamkamSRK59-E	Arabidopsis kamchatica subsp. kamchatica isolate 59 haplogroup E SRK (SRK) gene, complete cds.
Arabidopsis lyrata		AF328990	AlyrSRK05	Arabidopsis lyrata S-receptor kinase (SRK) gene, SRK-13-5 allele, exon 1 and partial cds.
Arabidopsis lyrata		AF328993	AlyrSRK13	Arabidopsis lyrata S-receptor kinase (SRK) gene, SRK-13-13 allele, exon 1 and partial cds.
Arabidopsis lyrata		AF328994	AlyrSRK04	Arabidopsis lyrata S-receptor kinase (SRK) gene, SRK-13-4 allele, exon 1 and partial cds.
Arabidopsis lyrata		AF328995	AlyrSRK20	Arabidopsis lyrata S-receptor kinase (SRK) gene, SRK-13-20 allele, exon 1 and partial cds.
Arabidopsis lyrata		AF328997	AlyrSRK23	Arabidopsis lyrata S-receptor kinase (SRK) gene, SRK-13-23 allele, exon 1 and partial cds.
Arabidopsis lyrata		AF328998	AlyrSRK19	Arabidopsis lyrata S-receptor kinase (SRK) gene, SRK-13-19 allele, exon 1 and partial cds.
Arabidopsis lyrata		AY186763	AlyrSRK02	Arabidopsis lyrata S-receptor kinase 13-2 gene, exon 1 and partial cds.
Arabidopsis lyrata		AY186765	AlyrSRK07	Arabidopsis lyrata S-receptor kinase 13-7 gene, exon 1 and partial cds.
Arabidopsis lyrata		AY186766	AlyrSRK08	Arabidopsis lyrata S-receptor kinase 13-8 gene, exon 1 and partial cds.
Arabidopsis lyrata		AY186767	AlyrSRK10	Arabidopsis lyrata S-receptor kinase 13-10 gene, exon 1 and partial cds.
Arabidopsis lyrata		AY186768	AlyrSRK11	Arabidopsis lyrata S-receptor kinase 13-11 gene, exon 1 and partial cds.
Arabidopsis lyrata		AY186769	AlyrSRK12	Arabidopsis lyrata S-receptor kinase 13-12 gene, exon 1 and partial cds.
Arabidopsis lyrata		AY186770	AlyrSRK14	Arabidopsis lyrata S-receptor kinase 13-14 gene, exon 1 and partial cds.
Arabidopsis lyrata		AY186772	AlyrSRK17	Arabidopsis lyrata S-receptor kinase 13-17 gene, exon 1 and partial cds.
Arabidopsis lyrata		AY186775	AlyrSRK28	Arabidopsis lyrata S-receptor kinase 13-28 gene, exon 1 and partial cds.
Arabidopsis lyrata		AY186776	AlyrSRK29	Arabidopsis lyrata S-receptor kinase 13-29 gene, exon 1 and partial cds.
Arabidopsis		DQ520282	AlyrSRK09	Arabidopsis lyrata S receptor kinase SRK09 gene, exon 1 and

lyrata				partial cds.
Arabidopsis lyrata		DQ520283	AlyrSRK16	Arabidopsis lyrata S receptor kinase SRK16 gene, exons 1 through 7 and partial cds.
Arabidopsis lyrata		DQ520284	AlyrSRK18	Arabidopsis lyrata S receptor kinase SRK18 gene, exon 1 and partial cds.
Arabidopsis lyrata		DQ520285	AlyrSRK22	Arabidopsis lyrata S receptor kinase SRK22 gene, exon 1 and partial cds.
Arabidopsis lyrata		DQ520287	AlyrSRK31	Arabidopsis lyrata S receptor kinase SRK31 gene, exon 1 and partial cds.
Arabidopsis lyrata		DQ520289	AlyrSRK37	Arabidopsis lyrata S receptor kinase SRK37 gene, exons 1 through 7 and partial cds.
Arabidopsis lyrata		EU878017	AlyrSRK27	Arabidopsis lyrata isolate AlSRK27 S receptor kinase (SRK) gene, partial cds.
Arabidopsis lyrata		EU878019	AlyrSRK33	Arabidopsis lyrata isolate AlSRK33 S receptor kinase (SRK) gene, partial cds.
Arabidopsis lyrata		EU878020	AlyrSRK34	Arabidopsis lyrata isolate AlSRK34 S receptor kinase (SRK) gene, partial cds.
Arabidopsis lyrata		EU878021	AlyrSRK35	Arabidopsis lyrata isolate AlSRK35 S receptor kinase (SRK) gene, partial cds.
Arabidopsis lyrata		EU878022	AlyrSRK39	Arabidopsis lyrata isolate AlSRK39 S receptor kinase (SRK) gene, partial cds.
Arabidopsis lyrata		EU878023	AlyrSRK42	Arabidopsis lyrata isolate AlSRK42 S receptor kinase (SRK) gene, partial cds.
Arabidopsis lyrata		EU878024	AlyrSRK43	Arabidopsis lyrata isolate AlSRK43 S receptor kinase (SRK) gene, partial cds.
Arabidopsis lyrata		EU878025	AlyrSRK44	Arabidopsis lyrata isolate AlSRK44 S receptor kinase (SRK) gene, partial cds.
Arabidopsis lyrata		FJ867321	AlyrSRK30	Arabidopsis lyrata SRK (SRK) gene, SRK30 allele, partial cds.
Arabidopsis lyrata		GQ351354	AlyrSRK06	Arabidopsis lyrata S-locus receptor kinase 6 (SRK6) gene, complete cds.
Arabidopsis lyrata		GQ351355	AlyrSRK25	Arabidopsis lyrata S-locus receptor kinase 25 (SRK25) gene, complete cds.
Arabidopsis lyrata		GQ915332	AlyrSRK03a	Arabidopsis lyrata haplotype AlSRK03a S-receptor kinase (SRK) gene, partial cds.
Arabidopsis lyrata		GQ915349	AlyrSRK37a	Arabidopsis lyrata haplotype AlSRK37a S-receptor kinase (SRK) gene, partial cds.
Arabidopsis lyrata		GQ915366	AlyrSRK01a	Arabidopsis lyrata haplotype AlSRK01a S-receptor kinase (SRK) gene, partial cds.
Arabidopsis lyrata	petraea	JX464644	AlyrpetSRK60	Arabidopsis lyrata subsp. petraea S-receptor kinase SRK60 (SRK) gene, partial cds.
Arabidopsis lyrata	petraea	JX464646	AlyrpetSRK62	Arabidopsis lyrata subsp. petraea S-receptor kinase SRK62 (SRK) gene, partial cds.
Arabidopsis lyrata	petraea	JX464647	AlyrpetSRK63	Arabidopsis lyrata subsp. petraea S-receptor kinase SRK63 (SRK) gene, partial cds.
Arabidopsis lyrata	petraea	JX464648	AlyrpetSRK64	Arabidopsis lyrata subsp. petraea S-receptor kinase SRK64 (SRK) gene, partial cds.
Arabidopsis lyrata	petraea	JX464649	AlyrpetSRK74b	Arabidopsis lyrata subsp. petraea S-receptor kinase SRK74 isoform b (SRK) gene, partial cds.
Arabidopsis lyrata	petraea	JX464650	AlyrpetSRK65	Arabidopsis lyrata subsp. petraea S-receptor kinase SRK65 (SRK) gene, partial cds.
Arabidopsis lyrata	petraea	JX464653	AlyrpetSRK67	Arabidopsis lyrata subsp. petraea S-receptor kinase SRK67 (SRK) gene, partial cds.
Arabidopsis lyrata	petraea	JX464655	AlyrpetSRK69	Arabidopsis lyrata subsp. petraea S-receptor kinase SRK69 (SRK) gene, partial cds.
Arabidopsis lyrata		KC207413	AlyrSRK15	Arabidopsis lyrata clone 1b S-locus receptor kinase 13-15 (SRK 13-15) gene, partial cds.
Arabidopsis lyrata		KC207416	AlyrSRK36	Arabidopsis lyrata S-receptor kinase (SRK36) gene, partial cds.
Arabidopsis lyrata	petraea	KF418159	AlyrpetSRK05	Arabidopsis lyrata subsp. petraea S-locus receptor kinase s-haplotype 1 (SRK1) mRNA, 1 allele, complete cds.
Arabidopsis thaliana		EF692486	AthaSRK-Chi-1	Arabidopsis thaliana ecotype Chi-1 (CS6665) SRK (SRK) pseudogene, partial sequence.
Arabidopsis thaliana		EF692490	AthaSRK-Col-0	Arabidopsis thaliana ecotype Col-0 (CS1092) SRK (SRK) pseudogene, complete sequence.

Arabidopsis thaliana		EU083397	AthaSRK-Bu-21	Arabidopsis thaliana ecotype Bu-21 (CS6652) SRK (SRK) pseudogene, complete sequence.
Arabidopsis thaliana		GU723860	AthaARK3-Wei-0	Arabidopsis thaliana ecotype Wei-0 (CS22622) ARK3 (ARK3) gene, partial cds.
Arabidopsis thaliana		GU723861	AthaARK3-Ws-0	Arabidopsis thaliana ecotype Ws-0 (CS22623) ARK3 (ARK3) gene, partial cds.
Arabidopsis thaliana		GU723873	AthaSRK-Wei-1	Arabidopsis thaliana ecotype Wei-1 (CS6925) SRK (SRK) gene, complete cds.
Arabidopsis thaliana		GU723874	AthaSRK-Ws-0	Arabidopsis thaliana ecotype Ws-0 (CS1602) SRK (SRK) gene, complete cds.
Capsella grandiflora		DQ530637	CgraSRK1	Capsella grandiflora S-receptor kinase (SRK) gene, SRK-1 allele, exon 1 and partial cds.
Capsella grandiflora		DQ530638	CgraSRK2	Capsella grandiflora S-receptor kinase (SRK) gene, SRK-2 allele, exon 1 and partial cds.
Capsella grandiflora		DQ530639	CgraSRK3	Capsella grandiflora S-receptor kinase (SRK) gene, SRK-3 allele, exon 1 and partial cds.
Capsella grandiflora		DQ530640	CgraSRK4	Capsella grandiflora S-receptor kinase (SRK) gene, SRK-4 allele, exon 1 and partial cds.
Capsella grandiflora		DQ530641	CgraSRK5	Capsella grandiflora S-receptor kinase (SRK) gene, SRK-5 allele, exon 1 and partial cds.
Capsella grandiflora		DQ530642	CgraSRK6	Capsella grandiflora S-receptor kinase (SRK) gene, SRK-6 allele, exon 1 and partial cds.
Capsella grandiflora		EF530735	CgraSRK07	Capsella grandiflora S-locus receptor kinase SRK7 (SRK7) gene, complete cds.
Capsella grandiflora		FJ613331	CgraSRK35	Capsella grandiflora putative S-receptor kinase (SRK) gene, SRK-35 allele, exon 1 and partial cds.
Capsella rubella		FJ649926	CrubSRK-1204	Capsella rubella ecotype 1204 SRK (SRK) pseudogene, partial sequence.
Capsella rubella		FJ649937	CrubSRK-690	Capsella rubella ecotype 690 SRK (SRK) pseudogene, partial sequence.
Capsella rubella		FJ649941	CrubSRK-844	Capsella rubella ecotype 844 SRK (SRK) gene, partial cds.
Capsella rubella		FJ649944	CrubSRK-907	Capsella rubella ecotype 907 SRK (SRK) gene, partial cds.
Capsella rubella		FJ649948	CrubSRK-MTE	Capsella rubella ecotype MTE SRK (SRK) pseudogene, partial sequence.
Ipomoea trifida		U20948	ItriIRK1	Ipomoea trifida receptor protein kinase (IRK1) mRNA, complete cds.
Leavenworthia alabamica		JQ397439	LalaSRK28	Leavenworthia alabamica isolate LaSRK28 S-locus receptor kinase (SRK) gene, partial cds.
Leavenworthia alabamica		JQ397443	LalaSRK39	Leavenworthia alabamica isolate LaSRK39 S-locus receptor kinase (SRK) gene, partial cds.
Leavenworthia alabamica		JQ397448	LalaSRKr	Leavenworthia alabamica isolate LaSRKr S-locus receptor kinase (SRK) gene, partial cds.

Supplementary Table 12. The list of sequences in NCBI Genbank used for the phylogenetic analysis of *SCR* gene.

Species	Subspecie s	Accession	Short Name	Description
Arabidopsi s halleri	gemmifera	GU723952	AhalgemSCR -A	Arabidopsis halleri subsp. gemmifera haplogroup A SCR (SCR) gene, complete cds.
Arabidopsi s halleri		KJ772374	AlyrSCR-S12	Arabidopsis halleri haplotype S12 clone 13p19 S-locus region genomic sequence.
Arabidopsi s halleri		KJ772397	AhalSCR-S20	Arabidopsis halleri haplotype S20 clone 18P18 S-locus region genomic sequence.
Arabidopsi s lyrata		FJ752546	AlyrSCR37	Arabidopsis lyrata S-locus cysteine-rich protein SCR37 (SCR) gene, SCR-37 allele, complete cds.
Arabidopsi s lyrata		GQ351357	AlyrSCR25	Arabidopsis lyrata S-locus cysteine-rich protein 25 (SCR25) gene, complete cds.
Arabidopsi s lyrata		KJ772401	AlyrSCR-S1	Arabidopsis lyrata haplotype S1 clone 29O9 S-locus region genomic sequence.
Arabidopsi		KJ772412	AlyrSCR-S18	Arabidopsis lyrata haplotype S18 clone 04A13 S-locus region genomic

s lyrata				sequence.
Arabidopsis thaliana		AY772638	AthaSCR-Cvi-0	Arabidopsis thaliana SCR-B (SCR-B at S-locus of haplogroup B) gene, complete cds; and SRK (SRK-B pseudogene at S-locus of haplogroup B) pseudogene, complete sequence.
Arabidopsis thaliana		EF692449	AthaSCR-Col-0-res	Arabidopsis thaliana Col-0 SCR (restored cds per Tsuchimatsu et al., 2012).
Brassica oleracea		AF195625	BoleSCR-S6	Brassica oleracea haplotype S6 S-locus cysteine-rich protein (SCR) mRNA, complete cds.
Brassica oleracea		AF195626	BoleSCR-S13	Brassica oleracea haplotype S13 S-locus cysteine-rich protein (SCR) mRNA, complete cds.
Brassica oleracea		AJ278643	BoleSCR3	Brassica oleracea mRNA for SCR3 protein (scr3 gene).
Brassica rapa	oleifera	AF195627	BrapSCR-S8	Brassica rapa haplotype S8 S-locus cysteine-rich protein (SCR) mRNA, complete cds.
Capsella grandiflora		EF530736	CgraSCR7	Capsella grandiflora S-locus cysteine-rich protein SCR7 (SCR7) gene, complete cds.
Raphanus sativus		AY422009	RsatSP11-1	Raphanus sativus S-locus pollen protein 11-1 (SP11-1) gene, complete cds.
Raphanus sativus		AY422010	RsatSP11-2	Raphanus sativus S-locus pollen protein 11-2 (SP11-2) gene, complete cds.
Raphanus sativus		AY422011	RsatSP11-3	Raphanus sativus S-locus pollen protein 11-3 (SP11-3) gene, complete cds.
Raphanus sativus		AY422012	RsatSP11-4	Raphanus sativus S-locus pollen protein 11-4 (SP11-4) gene, complete cds.
Raphanus sativus		AY422013	RsatSP11-5	Raphanus sativus S-locus pollen protein 11-5 (SP11-5) gene, complete cds.

Supplementary Table 13. Additional oligo-sequences used in this study.

Detailed name	Sequence 5' -> 3'	Reference
qPCR PLT5 FW	ACATCATCAGCATGGTCGATGG	
qPCR PLT5 RV	GCGGAATTTGATCGCTGCTATATCATAAG	
qPCR PLT7 FW	AGCAGCTAGAGCCTATGACTTG	
qPCR PLT7 RV	CCGCTACTTTTCCTCCTAAGAGATG	
qPCR TIP41 FW	GATGGTGTGCTTATGAGATTGAGAG	
qPCR TIP41 RV	TCAACTGGATACCCTTTCGCA	
qPCR AP2M FW	TCGATTGCTTGGTTTGGAAGATAAGA	
qPCR AP2M RV	TTCTCTCCCATTGTTGAGATCAACTC	
qPCR GAPC1 FW	TTCATCACTACTGAGTACATGAC	
qPCR GAPC1 RX	CGAAAACAGTGACTGGCTTC	
pPLV25 AtPLT5 FW	TAGTTGGAATAGGTTCATGAAGAACAATAACAACAAATCTTCTTCTTC	
pPLV25 AtPLT5 RV	AGTATGGAGTTGGGTTCTCATTCCAACCCAAAAACCGG	
pPLV25 AtPLT7 FW	TAGTTGGAATAGGTTC**ATG**GCTCCTCCAATGACGAATTG	
pPLV25 AtPLT7 RV	AGTATGGAGTTGGGTTC**TTA**GTAAGACTGGTTAGGCCACAAGA	
pPLV25 ChPLT5 FW	**TAGTTGGAATAGGTTC**ATGAACAACAACAATAACAACAAATCTTCTTCATC	
pPLV25 ChPLT5 RV	**AGTATGGAGTTGGGTTC**TCATTCCAACCCAAAAACCGGTG	
pPLV25 ChPLT7 FW	**TAGTTGGAATAGGTTC**ATGGTTCCAACGACGAATTGGTTAAC	
pPLV25 ChPLT7 RV	**AGTATGGAGTTGGGTTC**TTAGTAAGACTGGTTAGGCCACAAGAAAAAC	
pBJ36 AtCUC2 FW	GAGAGAATGCAT**TAGAGGAAGAGTTAAGAGATGAAGAAGAAGAAAG**	
pBJ36 AtCUC2 RV	TCTCTCGTCGAC**TAAGAAGAAAGATCTAAAGCTTTTGTTTGAGAGAG**	
CARHR043880 FW	AGGCAACTACAAAACTGTCG	
CARHR043880 RV	TTTGACTAAATAATGATTCAGATT	
CARHR044320 FW	GATTGCGTTTGGCGTTACTGTGAGA	
CARHR044320 RV	AAGCAGCGGCAGAGAGTTGGAG	

CARHR045840 FW	CTACAAAACTGTCAGAAGCCAAAACTG	
CARHR045840 RV	AGCAGCGTTAGAGAGTTGGTCAAAGC	
CARHR045850 FW	CCTCCATTAGTGCCAACGCTCT	
CARHR045850 RV	AATTCATCATACGGTGCCTTGCT	
Clathrin FW	TCGATTGCTTGGTTTGGAAGATAAGA	
Clathrin RV	TTCTCTCCCATTGTTGAGATCAACTC	
PLT5,7ImiR-s	gaTATAGATGCGCTTCGTGTCTCtctctcttttgtattcc	
PLT5,7IImiR-a	gaGAGACACGAAGCGCATCTATAtcaaagagaatcaatga	
PLT5,7IIImiR*s	gaGAAACACGAAGCGGATCTATTtcacaggtcgtgatatg	
PLT5,7IVmiR*a	gaAATAGATCCGCTTCGTGTTTCtctacatatatattcct	
ChGAPDH qRT F	TGACCACCGTCCACTCCATCAC	*
ChGAPDH qRT R	GCTCTTCCACCTCTCCAGTCCTTC	*
SCR F1	ACACAACATCTCATAGTAAGAACG	
SCR R1	TTATGAGGCCTATAGTAAAG	
SCR F2	ATTTTCTTAAGGTGTGAGAGAGT	
SCR R2	ACCACTAGACTGTAACGACTAT	
SRK F1	TCTAATTCTGTCTCGTCGCAC	
SRK R1	TCTTTCACATGATTTCCCTATTACT	
SRK F2	AGAGTCCTTAGATCTTGGCGA	
SRK R2	GAGAAATTGTCAGTGGCCCT	
SRK F5	GATTCGTTGGACATGACACG	

* *Kougioumoutzi E, Cartolano M, Canales C, Dupré M, Bramsiepe J, Vlad D, Rast M, Dello Ioio R, Tattersall A, Schnittger A, Hay A and Tsiantis M. SIMPLE LEAF3* encodes a ribosome-associated protein required for leaflet development in *Cardamine hirsuta*. Plant Journal **73(4)**, 533-45 (2013).

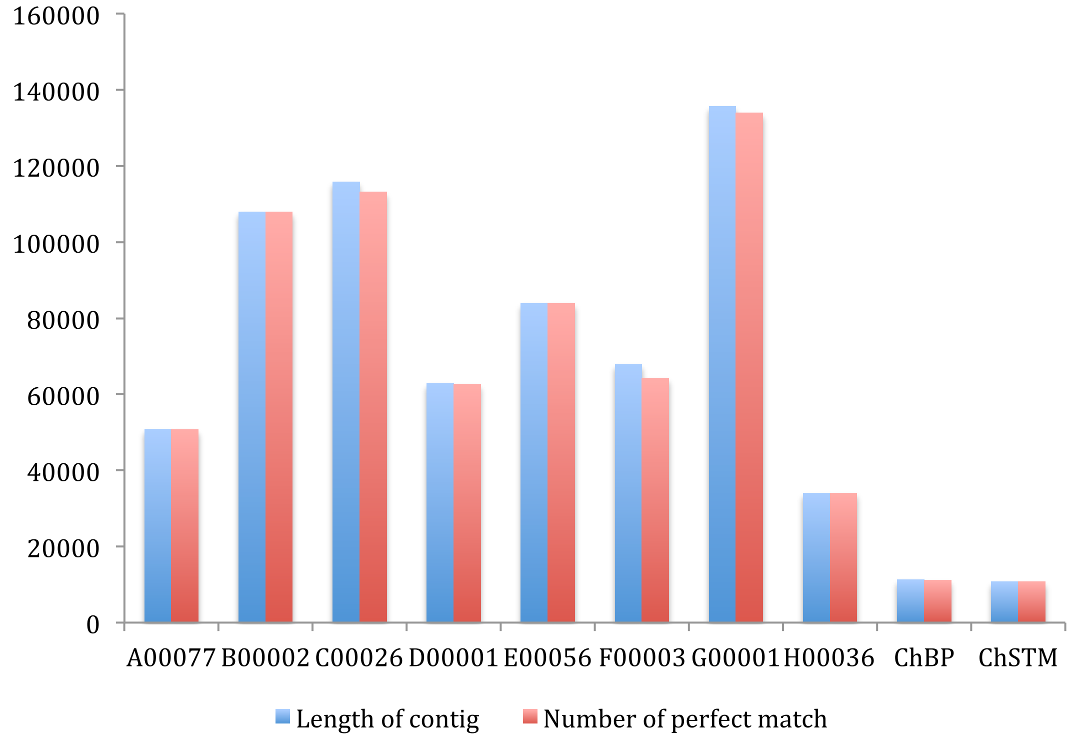

Fig. S1. Alignment ratio of 10 long pre-assembled regions to our *C. hirsuta* genome assembly.

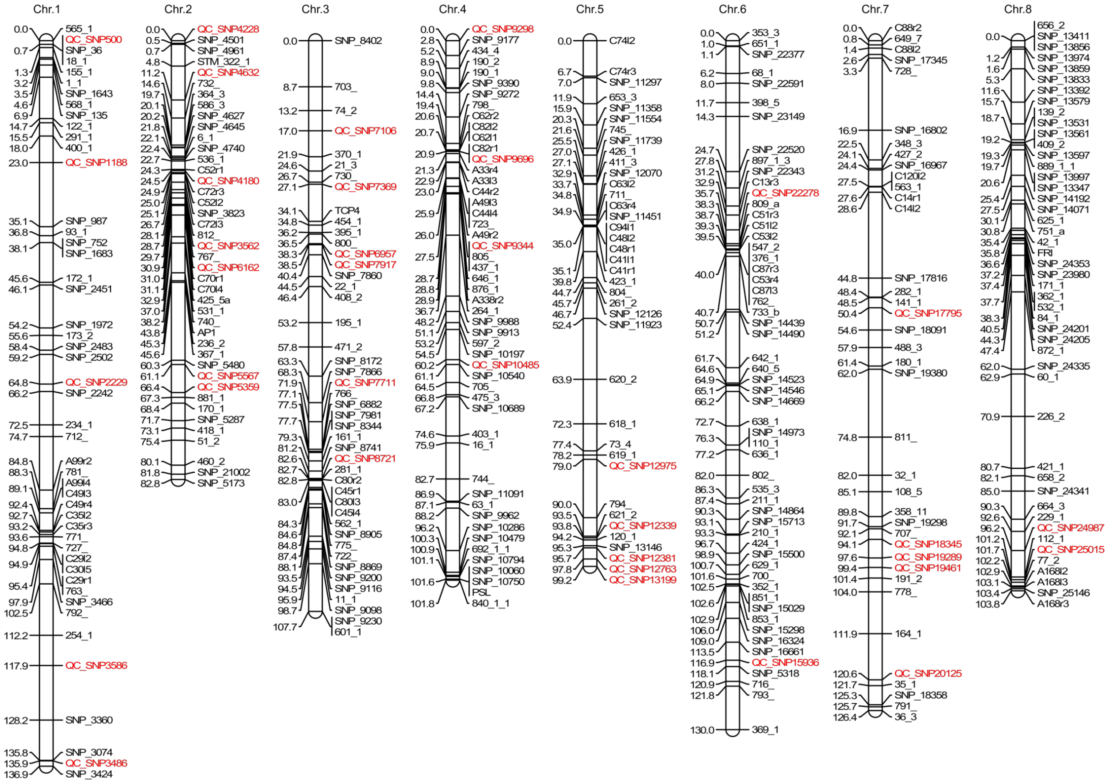

Fig. S2. Distribution of 328 genetic map markers and 36 additional quality control markers on the *C. hirsuta* genome.

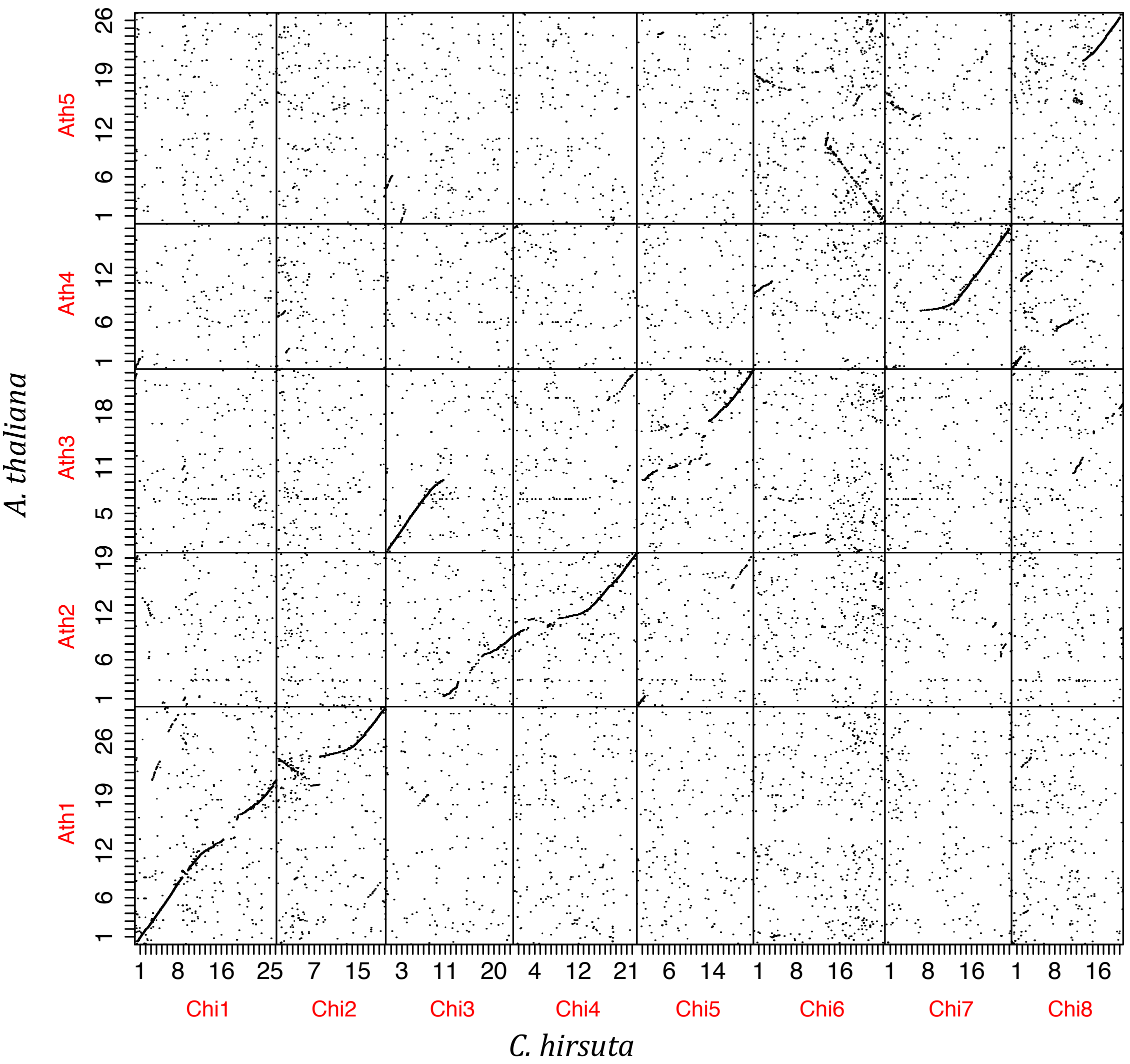
A. thaliana
Ath5
Ath4
Ath3
Ath2
Ath1
26
19
12
6
1
12
6
1
18
11
5
19
12
6
1
26
19
12
6
1
1 8 16 25
7 15
3 11 20
4 12 21
6 14
1 8 16
1 8 16
1 8 16
Chi1
Chi2
Chi3
Chi4
Chi5
Chi6
Chi7
Chi8
C. hirsuta

(**a**)

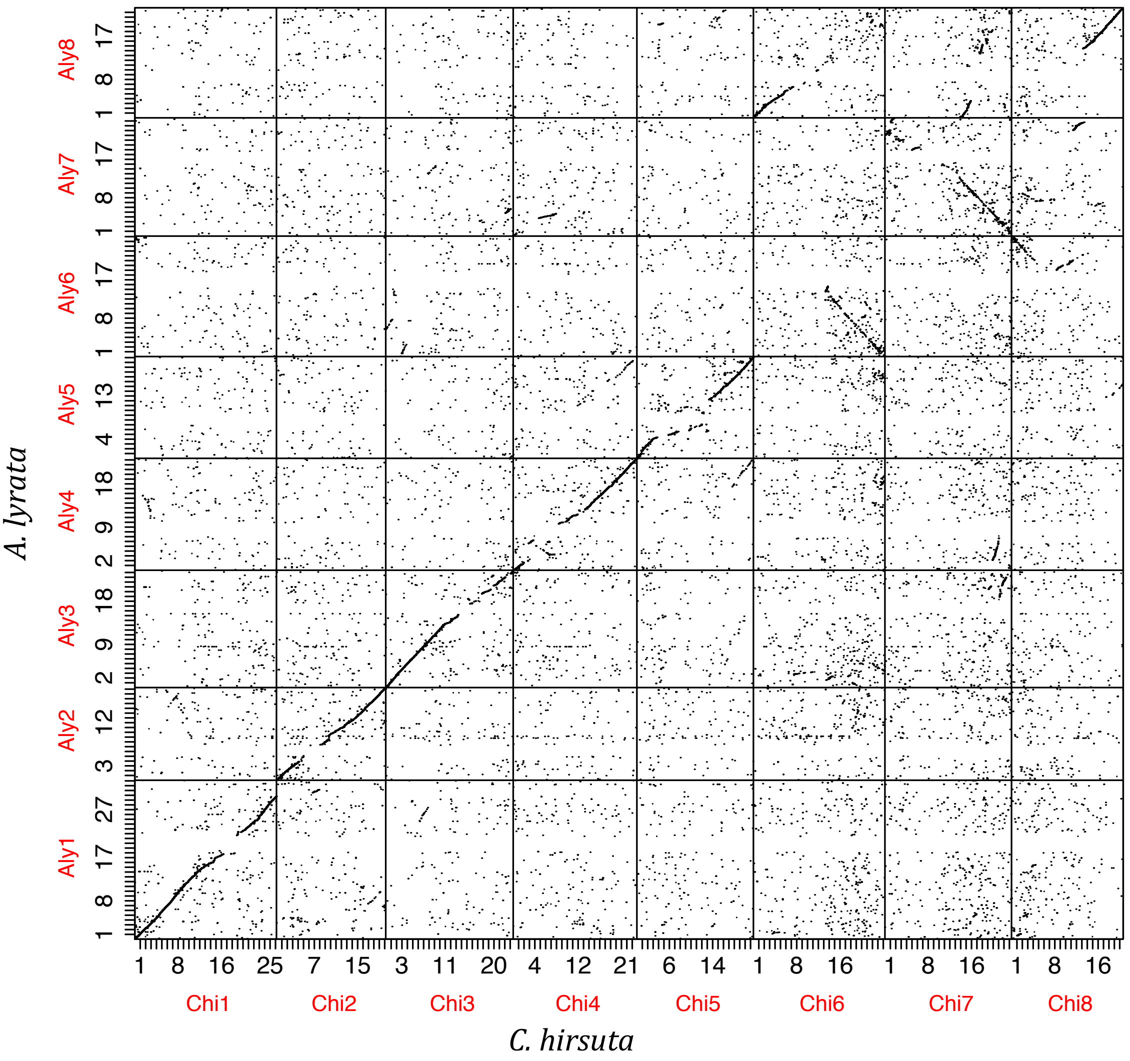
A. lyrata
Aly1
Aly2
Aly3
Aly4
Aly5
Aly6
Aly7
Aly8
C. hirsuta
Chi1
Chi2
Chi3
Chi4
Chi5
Chi6
Chi7
Chi8

(**b**)

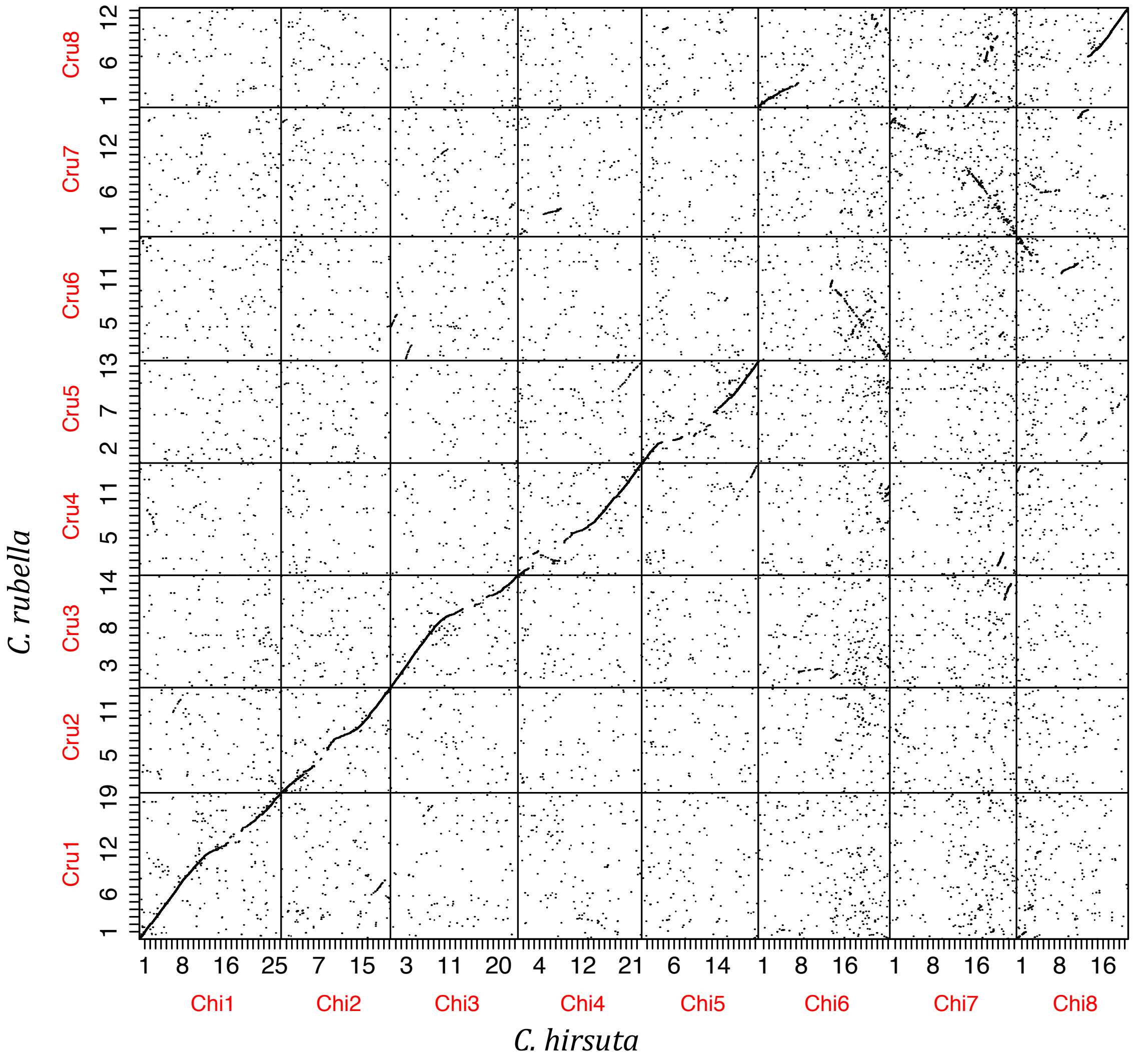

(**c**)

Fig. S3. Syntenic block scatter-plot of *C. hirsuta* against *A. thaliana* (**a**), *A. lyrata* (**b**) and *C. rubella* (**c**).

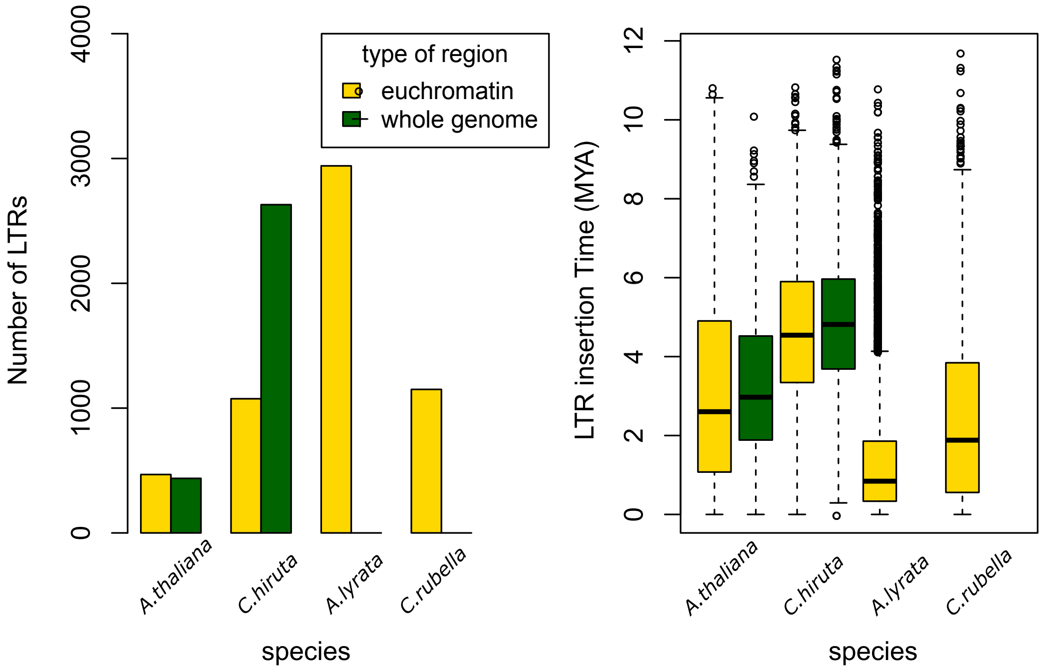

Fig. S4. LTR distribution in the genomes of *A. thaliana*, *C. hirsuta*, *A. lyrata* and *C. rubella*.

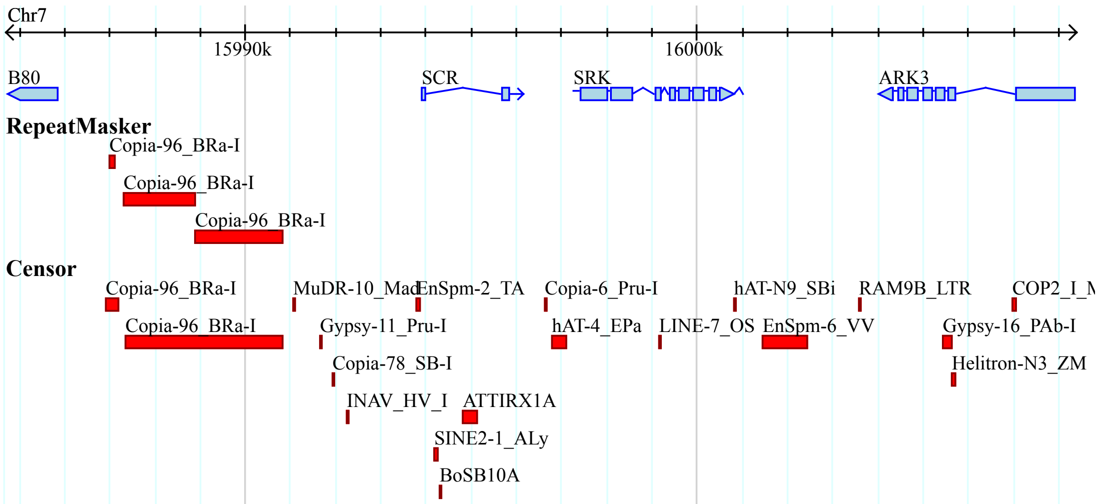

(a)

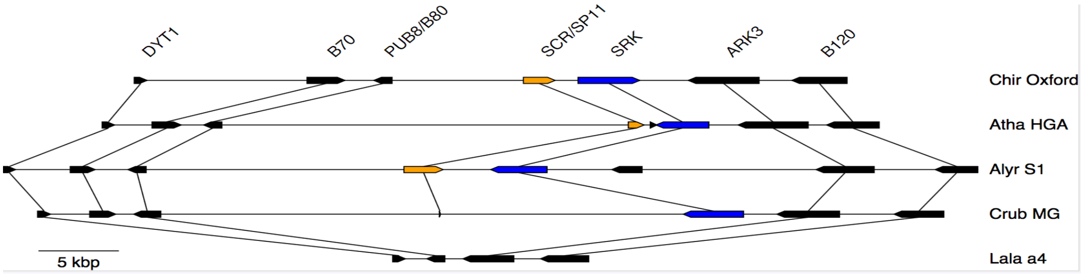

(b)

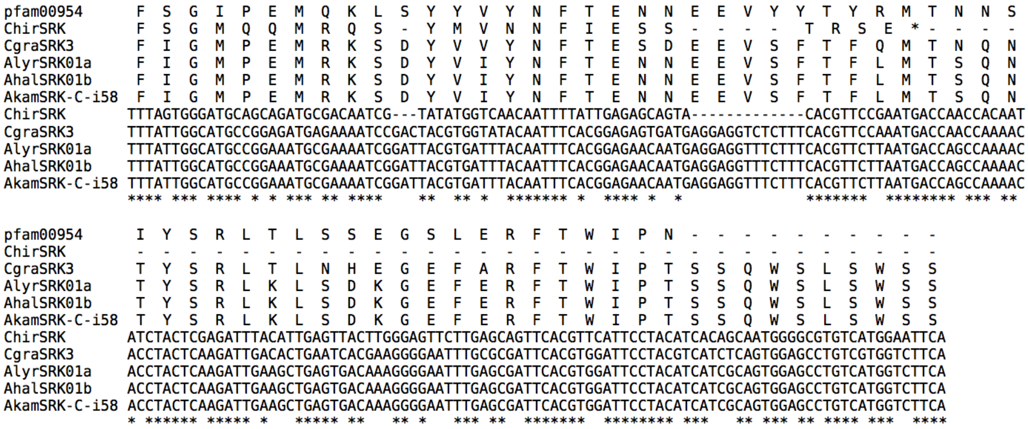

(c)

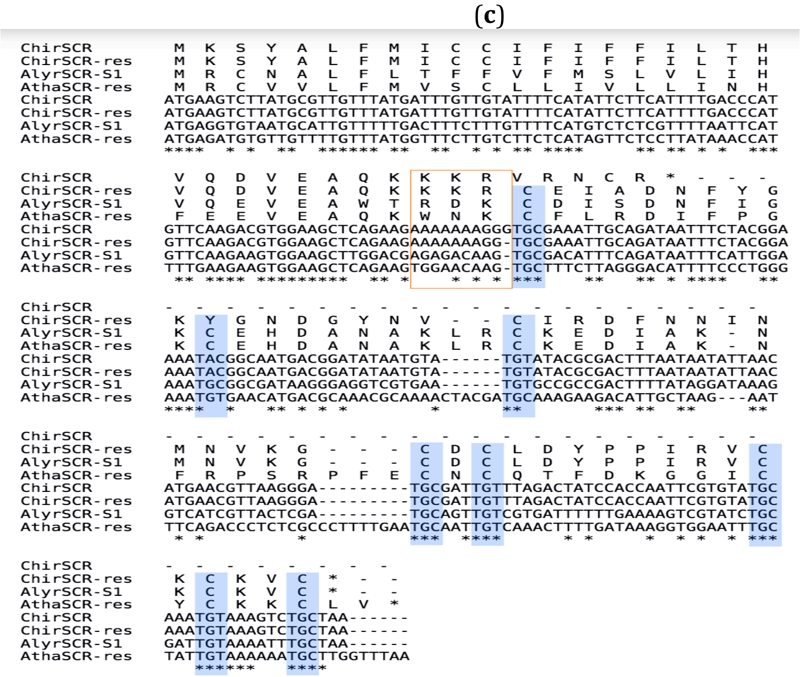

(d)

Fig. S5. Characterization of *SRK/SCR S*-locus in *C. hirsuta.* (**a**) The *S*-locus encompassing *ChSRK* and *ChSCR* resides on chromosome 7 in *C. hirsuta*, flanked by *ARK3* and *B80/PUB8* genes. The *SRK* gene model was obtained by removing erroneous splicing sites from the model predicted by Augustus. The *SCR* gene model was reconstructed based on alignment to *SCR* genes from other species. The *S*-locus of *C. hirsuta* encompassed many repetitive elements that are typical among functional S-haplotypes and spanned about 18 kb, which is in the range of non-functional S-haplotypes of self-compatible *A. thaliana* (about 15-31 kb)[15,58] but smaller than functional S-haplotypes of *Arabidopsis* and *Brassica* (about 28-110 kb)[59]. (**b**) The *C. hirsuta S*-locus is syntenic to the *S*-locus of *A. thaliana, A. lyrata* and *Capsella rubella* and is located at the ancestral position of the *S*-locus in the Brassicaceae. *Leavenworthia alabamica*, a close relative of *Cardamine*, has evolved a secondary *S*-locus[16]. (**c**) Alignment of *SRK* sequences from *C. hirsuta, Capsella grandiflora*, *A. lyrata*, *A. thaliana* and *A. kamchatica* with the conserved *S*-locus glycoprotein domain sequence pfam00954. *ChSRK* is disrupted by a frameshift caused by a 13 bp deletion in the first exon that introduces a stop codon. *ChSRK* amino acid sequence corresponds to the coding sequence that lacks splicing predicted by Augustus. (**d**) Alignment of *SCR* sequences from *C. hirsuta, A. lyrata*, and *A. thaliana. ChSCR* is disrupted by a frameshift caused by an insertion mutation (1 bp or 4 bp depending on alignment) in the second exon, indicated by an orange box. ChirSCR-res and AthaSCR-res sequences are hypothetical alignments that restore amino acid sequence in order to assess conservation of eight cysteine residues highlighted in blue. The second cysteine residue is not conserved in *C. hirsuta*, although this mutation might have occurred after protein function was disrupted**.** Loss-of-function mutations in *ChSRK* and *ChSCR* were confirmed by cDNA Sanger sequencing.

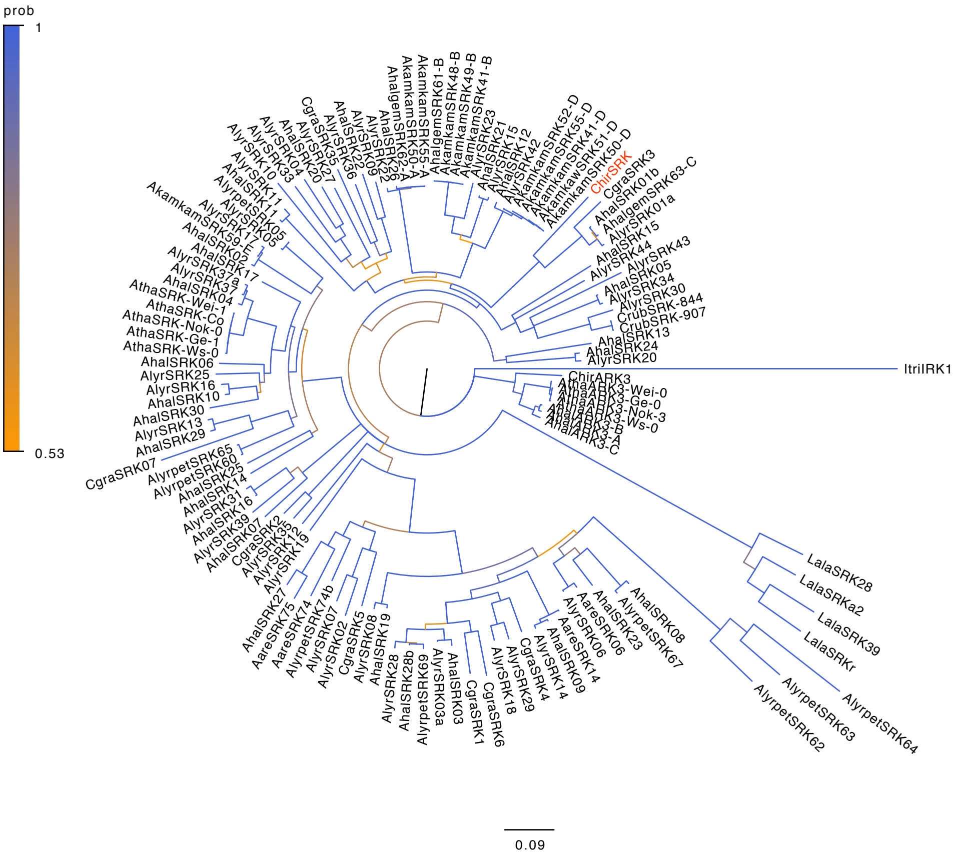

(a)

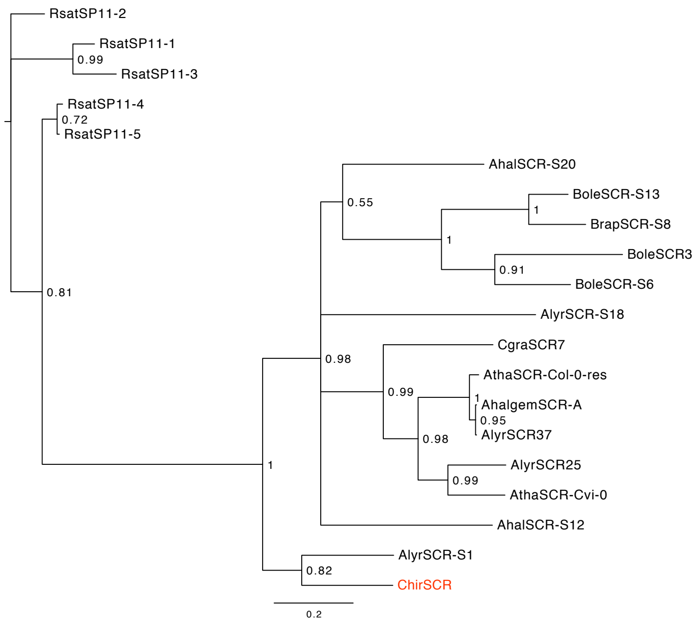

(b)

Fig. S6. Phylogenetic trees of *SRK* and *SCR* constructed from a group of close and distant relatives of *C. hirsuta* using CDS sequences. (**a**) The *SRK* gene of *C. hirsuta* is nested in the clade of trans-specifically segregating *SRK* haplogroups of many Brassicaceae species including those of *Arabidopsis* and *Capsella,* and is closest to *CgSRK3* and the *SRK* haplogroup 1 alleles in *A. halleri* and *A. lyrata*, which are the most recessive *S*-haplogroups with the highest frequency in outcrossing populations of *A. lyrata* and *A. halleri*[59]. *Lal2* haplotypes of *Leavenworthia* (shown as *LalaSRK*), another member of the tribe *Cardamineae*[69], did not cluster with *ChSRK*. Taken together with the synteny analysis in Fig. S6, this confirms that *Leavenworthia* evolved a secondary *S*-locus[16] and shows that *C. hirsuta* retained the ancestral *S*-locus. (**b**) The *SCR* gene of *C. hirsuta* is nested in the *SCR* clade and is closest to the S1 haplotype of *A. lyrata*.

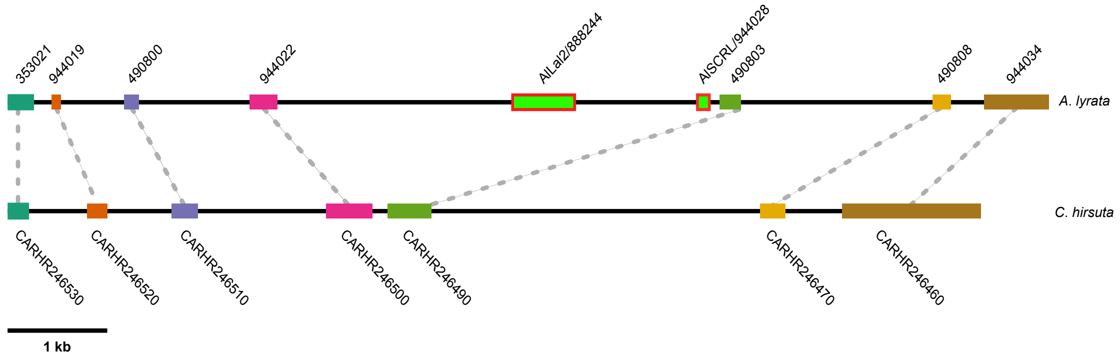

Fig. S7. Chromosomal organization and gene structure in a syntenic region of chromosome 7 in *A. lyrata* and *C. hirsuta*. This genomic region on chromosome 7 of *A. lyrata* was shown to be syntenic with the *Lal2/SCRL S*-locus region of Leavenworthia. Loss of *Lal2/SCRL* in *C. hirsuta* is caused by a long deletion.

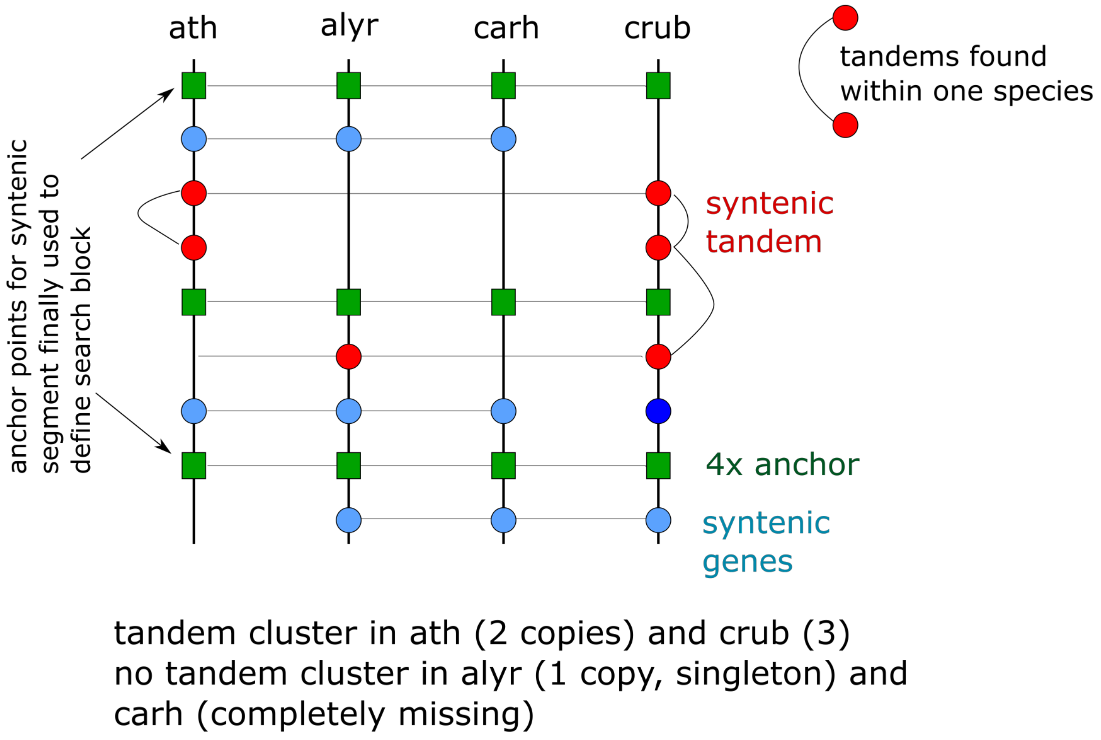

Fig. S8. Synteny-based approach to identify tandem gene duplications.

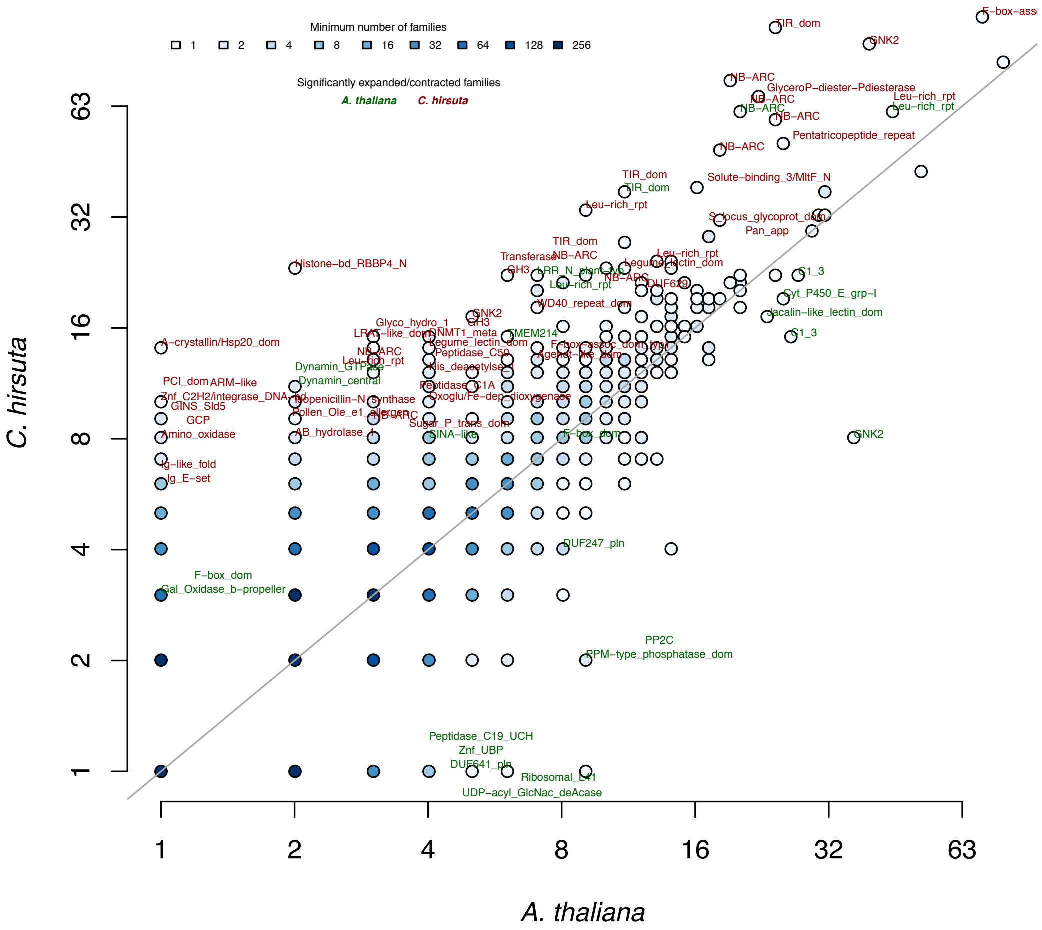

Fig. S9. Species-specific expansion and contraction of gene family clusters. Gene families are plotted on a logarithmic scale to show the number of family members in *A. thaliana* (x-axis) and *C. hirsuta* (y-axis).

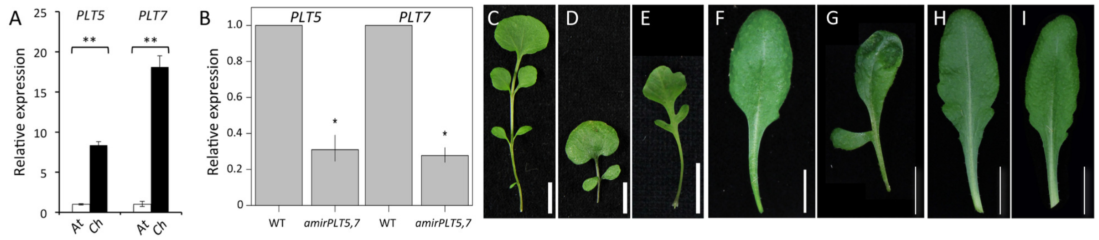

Fig. S10. Role for PLT5/7 in *C. hirsuta* leaf shape. (A, B) Relative expression of *PLT5* and *PLT7* in young leaves of *A. thaliana* and *C. hirsuta* (A) and in leaves of *C. hirsuta* wild type and *35S::amirPLT5,7* (B). Error bars show standard error (A) and standard deviation (B); ** Student's t-test p < 0.01 (A); * likelihood ratio test p < 0.05 (B). (C-E) Rosette leaf 5 of *C. hirsuta* wild type (C) and additional *35S::aMirPLT5,7* lines (D, E) to those shown in Fig. 3 of the main text. (F-I) Representative rosette leaves of *A. thaliana* vector-only control (F), *CUC2::AtPLT7* (G), *CUC2::ChPLT5* (H) and *CUC2::AtPLT5* (I). N = 20 (F), 4/14 (G), 50 (H) and 48 (I) independent transgenic lines with similar phenotype. Scale bar: 1 cm.

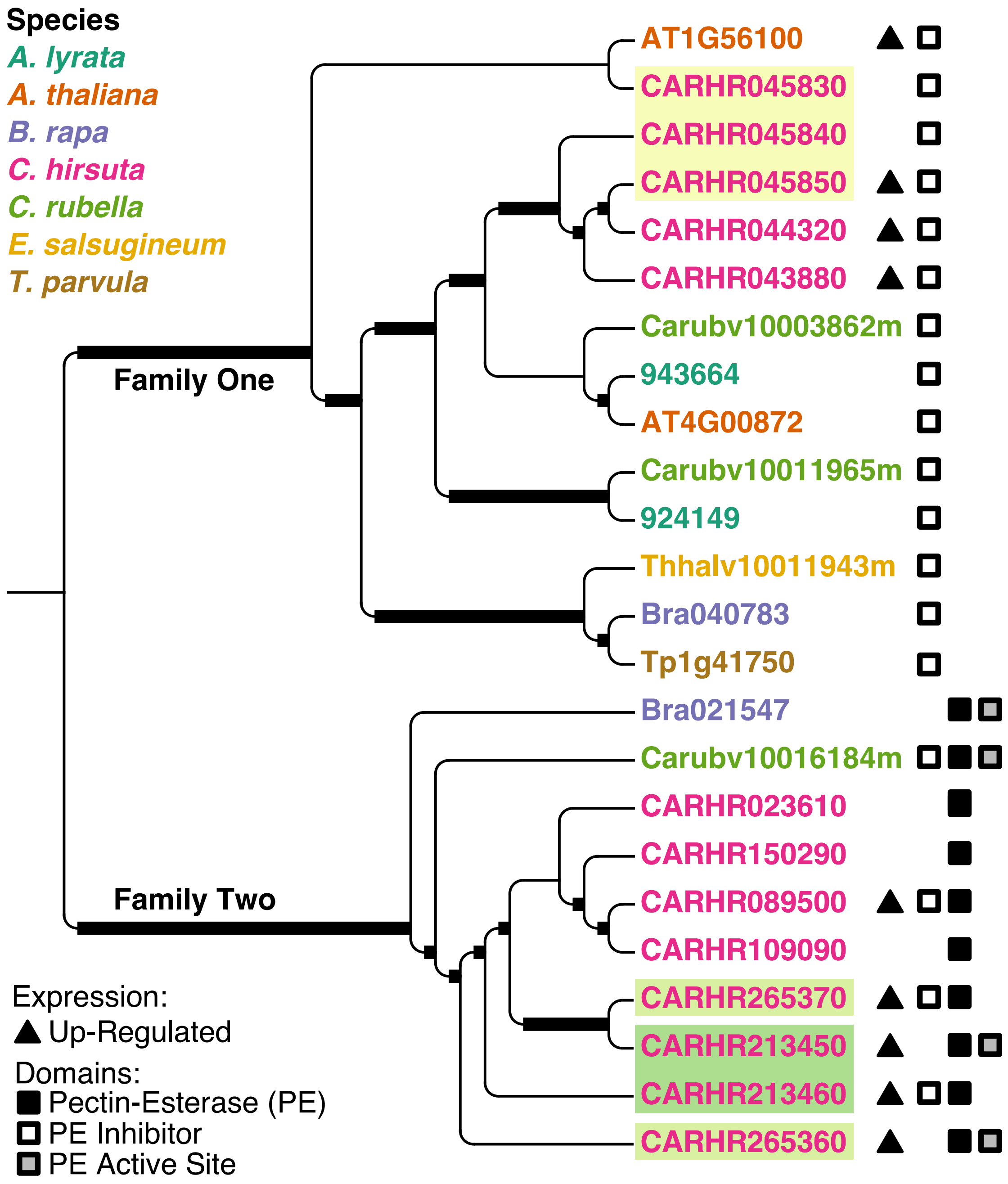

Fig. S11. Phylogenetic maximum likelihood tree[19] of expanded *PME(I)* gene families in *C. hirsuta*. Gene identifiers are color-coded to indicate species; tandem gene duplicates are highlighted in yellow, light green and dark green; triangles indicate significant up-regulation during fruit development; boxes indicate which conserved protein domains[21] are found in each gene: white, PMEI (IPR006501); black, PME catalytic (IPR000070) and pectin lyase (IPR011050, IPR012334); grey, PME active site (IPR018040). Bold branches have maximum confidence[31].

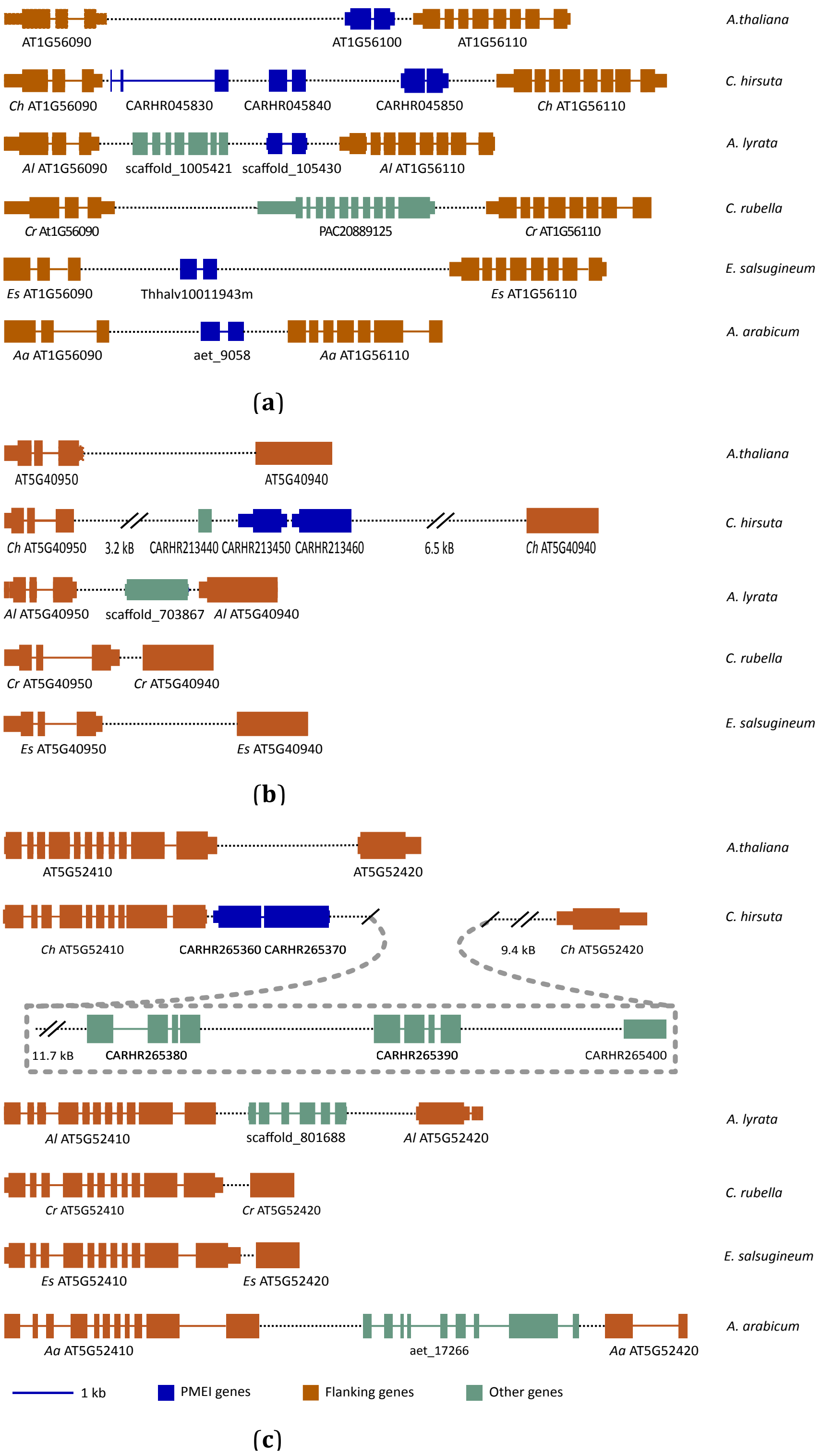

Fig. S12. Chromosomal organization and gene structure of three *PMEI* tandem gene duplications in crucifers. (**a**) CARHR045840-CARHR045850-CARHR045860 region. This region in *C. hirsuta* contains 3 *PMEI* genes while in *A. thaliana*, *A. lyrata, E. salsugineum* and *A. arabicum* this region contains a single *PMEI* gene and this gene is lost in *C. rubella*. (**b**, **c**) These regions in *C. hirsuta* contain *PMEI* genes, which are completely absent in the other species. (**b**) CARHR233450-CARHR233460 region; this region cannot be identified in the current assembly of *A. arabicum*. (**c**) CRHR265360-CRHR265370 region. In summary, all three tandem duplications events for *PME(I)* genes in *C. hirsuta* are species-specific.

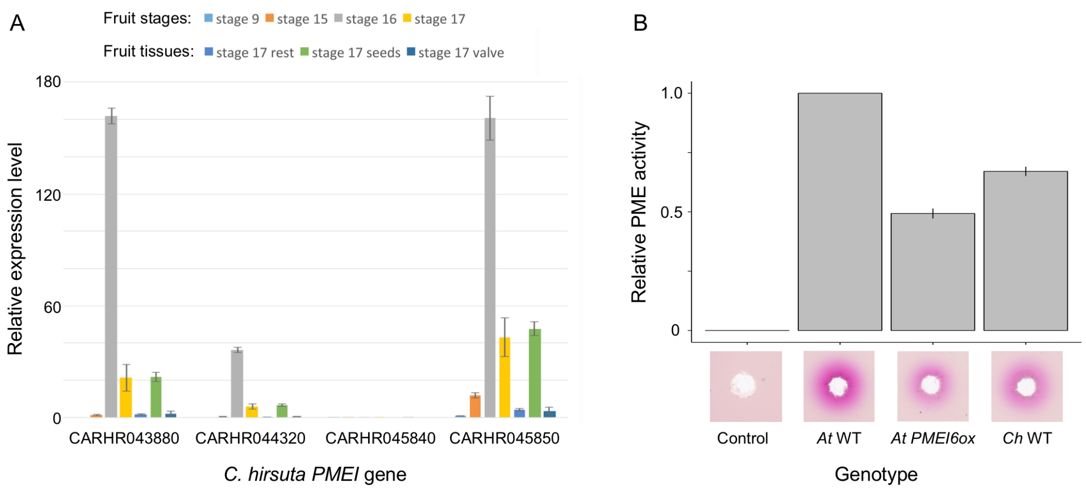

Fig. S13. (**A**) *PMEI* gene expression is significantly up-regulated during *C. hirsuta* fruit development. Relative expression levels of the *C. hirsuta PMEI* genes CARHR043880, CARHR044320, CARHR045840 and CARHR045850 determined by qRT-PCR. Three genes shown to be up-regulated during *C. hirsuta* fruit development by RNAseq: CARHR043880, CARHR044320 and CARHR045850, were significantly up-regulated at stage 16 (grey), and the expression observed in stage 17 fruit (yellow) was localised to seeds (green) rather than valve (light blue) or other fruit tissues (dark blue). Error bars show standard error of the mean. (**B**) Relative PME activity in 15 μg protein extracts from seeds of *A. thaliana* wild type, *A. thaliana PMEI6ox,* and *C. hirsuta* wild type, quantified from ruthenium red-stained gel assays (representative assays shown below graph); control assay contains no protein.

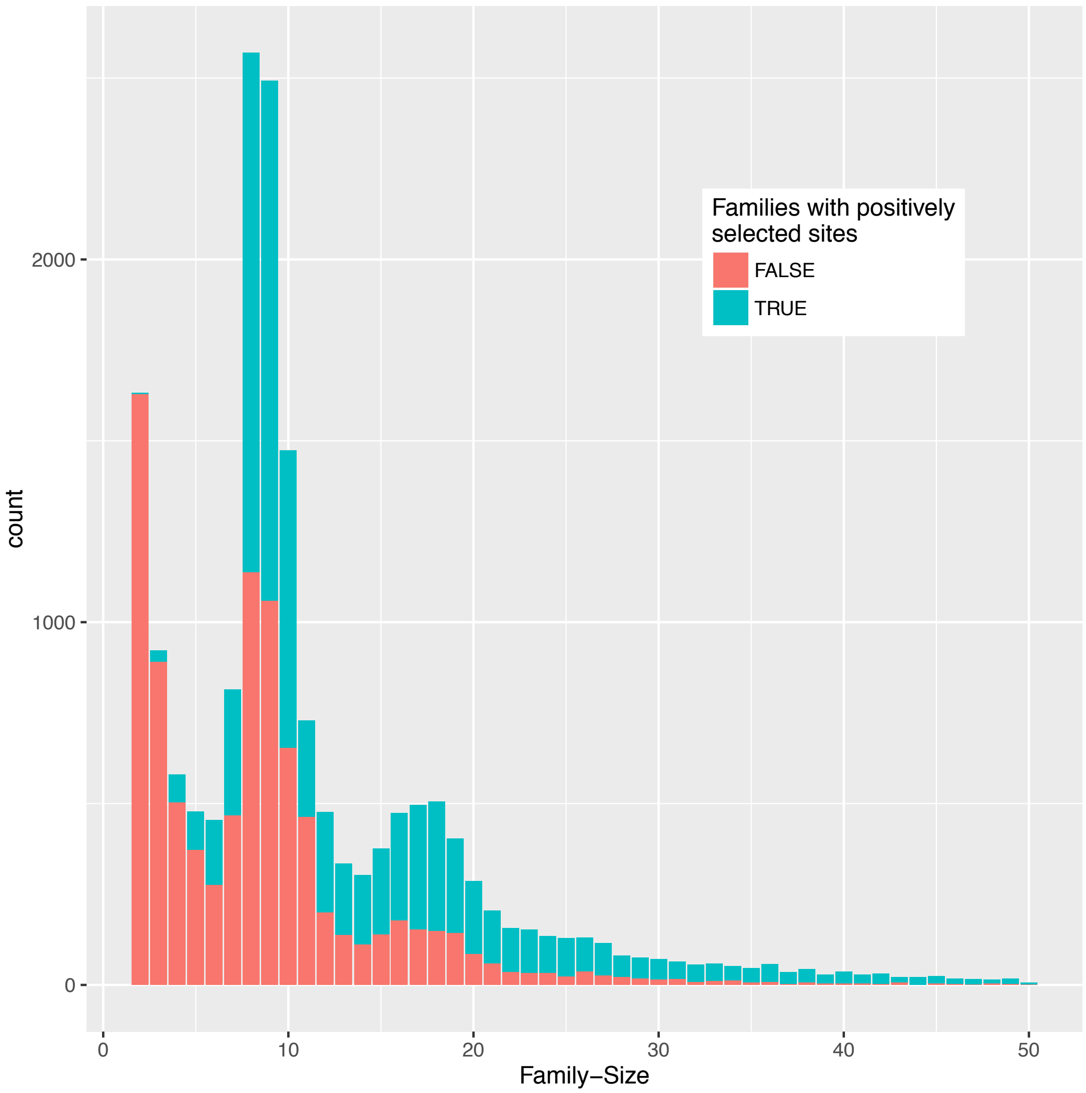

Fig. S14. The size distribution of gene families with sites subject to positive selection. Analysis of 17,827 non-singleton gene families in *A. arabicum*, *A. thaliana*, *A. lyrata*, *B. rapa*, *C. rubella*, *C. hirsuta, E. salsugineum,* and *S. parvula* identified 8866 families containing at least one selected homologous codon subject to positive selection, including the *PLT5/7* family, as well as pectin methylesterase inhibitor (PMEI) family I (Supplementary Methods). These families are significantly enriched in tandemly duplicated genes ($p < 7.04e-189$) and genes found in expanded families ($p < 3.7e-11$). Among those genes found in expanded families with positively selected sites, transcription factors are significantly overrepresented ($p < 3.41e-3$), as well as DEGs identified during fruit ($p < 3.7e-3$) and leaf ($p < 1.8e-2$) development. The histogram shown here indicates that gene family size has a moderate, but not a dominant, influence on the statistical power to detect positive selection, when the size of the family is larger than 3.

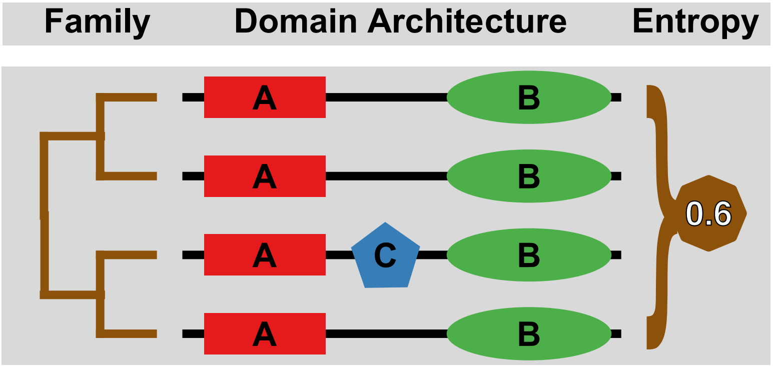

Fig. S15: Cartoon illustrating the method of Shannon Entropy for quantifying domain architecture diversity in gene families. In this example, two distinct domain architectures are found in the gene family: A, B, and A, B, C with a frequency 0.25 and 0.75 respectively. The Shannon Entropy = -0.25* log(0.25)- 0.75*log(0.75) ≈ 0.6.

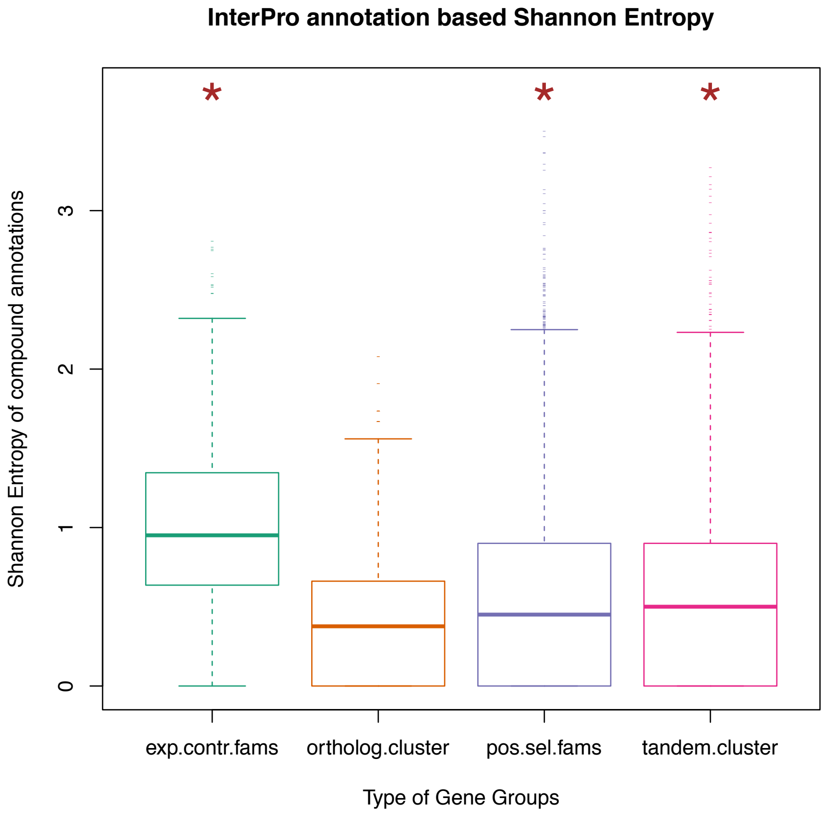

Fig. S16: Functional diversity distributions measured separately for different types of gene clusters: “exp.contr.fams” refers to expanded or contracted gene families, “ortholog.cluster” to orthologous gene clusters, “pos.sel.fams” to gene families showing signs of positive selection, and “tandem. cluster” to clusters of tandemly duplicated genes. Asterisk (*) indicates where the functional diversity distributions were significantly greater than the orthologous gene clusters (Kolmogorov-Smirnov test, $p < 0.05$). In addition, the expanded/contracted gene families show significantly greater diversity than the tandemly duplicated gene clusters, indicating greater domain conservation in tandemly duplicated genes (Kolmogorov-Smirnov test, $p < 2.2e-16$).

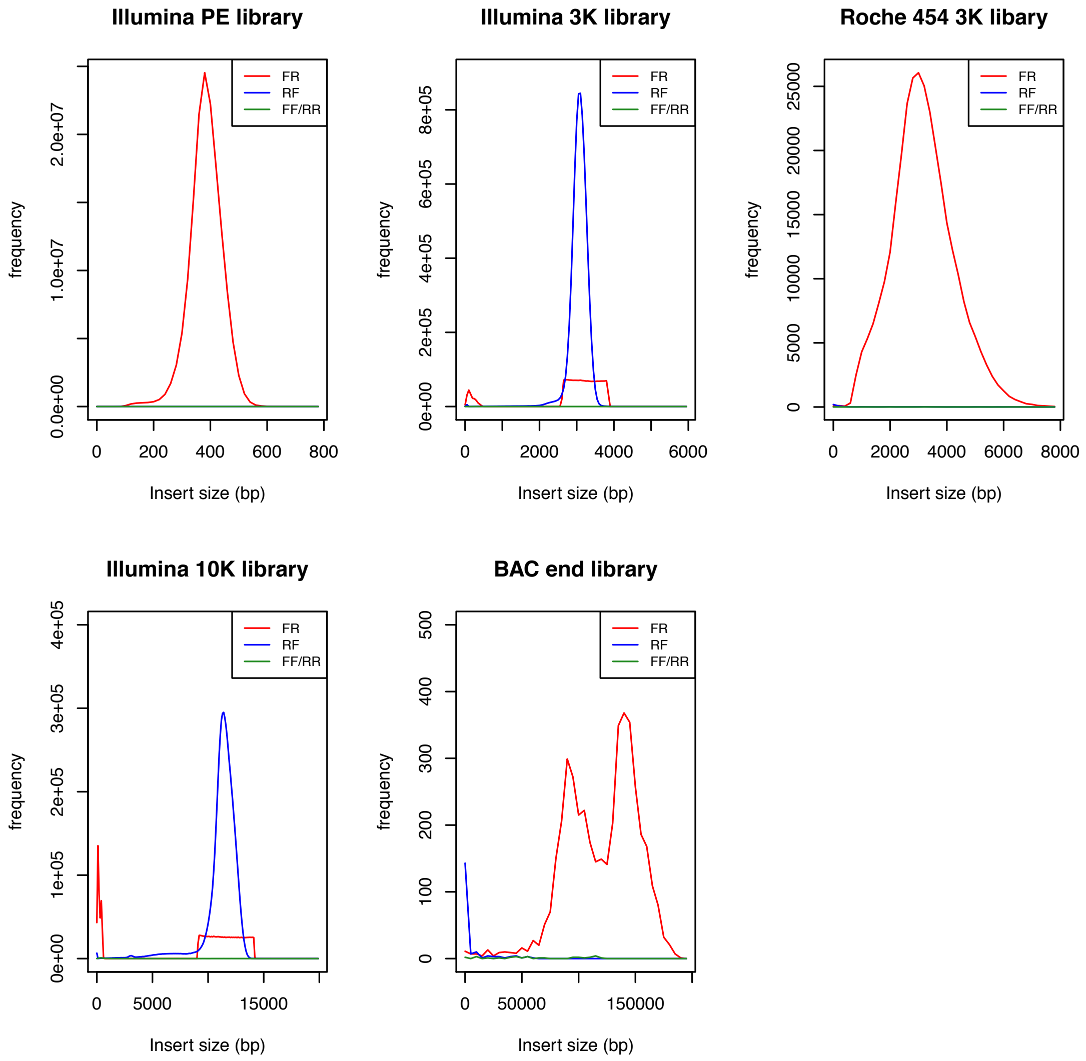

Fig. S17. The distributions of the insert size and orientation for multiple libraries of sequence data used for the assembly.

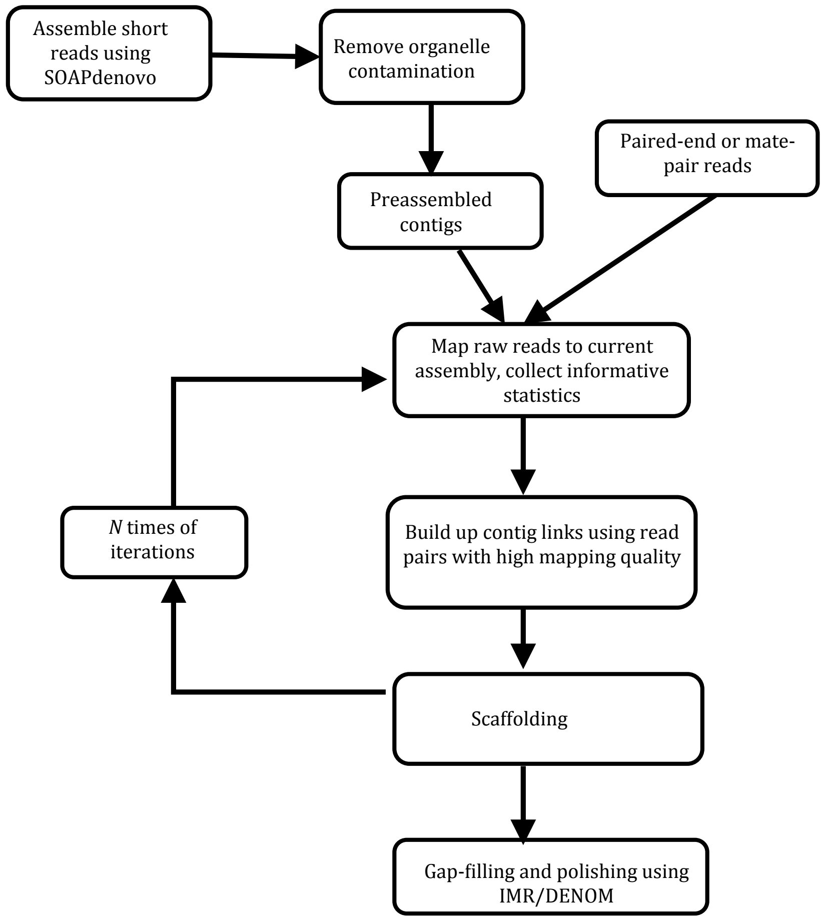

Fig. S18. Overview of genome assembly workflow.
